# Dynamic θ Frequency Coordination within and between the Prefrontal Cortex-Hippocampus Circuit during Learning of a Spatial Avoidance Task

**DOI:** 10.1523/ENEURO.0414-21.2022

**Published:** 2022-04-21

**Authors:** Conor R. Dickson, Gregory L. Holmes, Jeremy M. Barry

**Affiliations:** Epilepsy Development and Cognition Group, Department of Neurological Sciences, University of Vermont, Larner College of Medicine, Burlington 05405, VT

**Keywords:** active avoidance, coherence, hippocampus, prefrontal cortex, spatial cognition

## Abstract

θ-Scale coordination of prelimbic medial prefrontal cortex (mPFC) local field potentials (LFPs) and its influence via direct or indirect projections to the ventral hippocampus (vHC) and dorsal hippocampus (dHC) during spatial learning remains poorly understood. We hypothesized that θ frequency coordination dynamics within and between the mPFC, dHC, and vHC would be predetermined by the level of connectivity rather than reflecting differing circuit throughput relationships depending on cognitive demands. Moreover, we hypothesized that coherence levels would not change during learning of a complex spatial avoidance task. Adult male rats were bilaterally implanted with EEG electrodes and LFPs recorded in each structure. Contrary to predictions, θ coherence averaged across “Early” or “Late” training sessions in the mPFC-HC, mPFC-mPFC, and HC-HC increased as a function of task learning. Coherence levels were also highest between the indirectly connected mPFC-dHC circuit, particularly during early training. Although mPFC postacquisition coherence remained higher with dHC than vHC, dynamic mPFC coherence patterns with both hippocampal poles across avoidance epochs were similar. In the 3 s before avoidance, a regional temporal sequence of transitory coherence peaks emerged between the mPFC-mPFC, the mPFC-HC, and then dHC-dHC. During this sequence, coherence within θ bandwidth fluctuated between epochs at distinct subfrequencies, suggesting frequency-specific roles for the propagation of task-relevant processing. On a second timescale, coherence frequency within and between the mPFC and hippocampal septotemporal axis change as a function of avoidance learning and cognitive demand. The results support a role for θ coherence subbandwidths, and specifically an 8- to 9-Hz mPFC θ signal, for generating and processing qualitatively different types of information in the organization of spatial avoidance behavior in the mPFC-HC circuit.

## Visual Abstract

**Figure:**
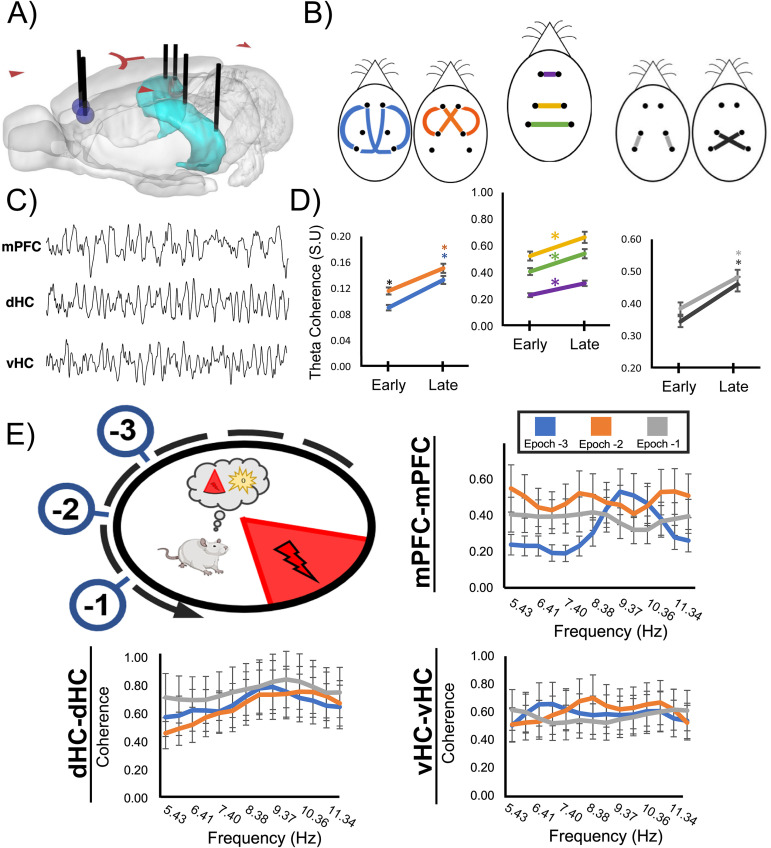

## Significance Statement

Whether the degree of signal coordination in neural circuits is superseded by levels of physiological connectivity or is also influenced by phases of learning and cognitive demand remains poorly understood. We aimed to determine whether θ oscillations in the medial prefrontal cortex (mPFC) were more coherent with the directly connected ventral hippocampus (vHC) rather than the indirectly connected dorsal hippocampus (dHC). Surprisingly, mPFC coherence with the dHC tended to be higher than the vHC, particularly in the early stages of learning an active avoidance task. We also found unique θ subfrequency coherence signals between mPFC hemispheres that were related to the generation of the avoidance response. While these signals were coherent with both hippocampal poles, they were not seen in intrahippocampal coherence. The results support other studies claiming that θ subfrequencies are important for avoidance behavior and further illustrate how narrow frequency signals represent dynamic throughput in neural circuits during cognition.

## Introduction

θ-Scale coordination within and between the medial prefrontal cortex (mPFC) and hippocampus (HC) has been implicated in spatial cognitive outcomes in neurodevelopmental disease models such as schizophrenia (Lee et al., 2012), early-life seizures (Kleen et al., 2011; Holmes et al., 2015; Niedecker et al., 2021), and cortical dysplasia (Hernan et al., 2020). The mPFC has long been directly connected to working memory, behavioral flexibility, and planning; all of which are critical faculties for goal-oriented behavior (Vinken et al., 1969; Fuster, 1989; Passingham, 1993; Spiers, 2008; O’Neill et al., 2013; Spellman et al., 2015; Tamura et al., 2017). In this vein, cell activity in the mPFC has also been proposed to have an important role in avoidance learning in aversive contexts (Adhikari et al., 2010; Padilla-Coreano et al., 2016, 2019) and the extinction-learning of aversive fear conditioning stimuli (Santini et al., 2004; Quirk et al., 2006; Peters et al., 2009; Rosas-Vidal et al., 2014, 2018; Martínez-Rivera et al., 2019). The HC θ phase coordination of mPFC cell activity has also been closely linked to learning and spatial processing (Hyman et al., 2005; Jones and Wilson, 2005; Siapas et al., 2005; Negrón-Oyarzo et al., 2018; Zielinski et al., 2019; Niedecker et al., 2021) and may direct attention to task-relevant representations required to solve spatial or working memory tasks (Hyman et al., 2010, 2011; O’Neill et al., 2013; Ito et al., 2015).

While much has been gleaned about mPFC-HC cell and field potential properties in relation to behavior, many details regarding mPFC-HC θ frequency coordination in relation to spatial learning remain unknown. Although slow frequency oscillations such as θ provide long-range windows of temporal organization (Siapas et al., 2005; Lee et al., 2012; 80–20 ms per cycle), it is not known whether there are meaningful differences in the degree of θ coordination between the mPFC and its monosynaptic connections to the ventral hippocampus (vHC; Jay and Witter, 1991; Jay et al., 1996; Hoover and Vertes, 2007; Liu and Carter, 2018; Anastasiades and Carter, 2021) and its indirect, polysynaptic connections to the dorsal hippocampus (dHC) via bidirectional cortical intermediaries and thalamic nuclei (Thierry et al., 2000; Ito et al., 2015; Eichenbaum, 2017; Dolleman-van der Weel et al., 2019; Anastasiades and Carter, 2021). Moreover, while levels of mPFC-HC phase coordination can correlate with or be predictive of spatial cognitive outcomes (Lee et al., 2012; Niedecker et al., 2021), and 8-Hz coordination has been shown to be necessary for learned avoidance (Padilla-Coreano et al., 2019), it is not known whether shifts to specific θ frequencies reflect changes in cognitive demand and circuit throughput during spatial avoidance in a similar manner as cortical processing of sensory stimuli (Tort et al., 2010; Schulz et al., 2015).

Different frequencies within the θ bandwidth have been characterized in rodents and ascribed different functions. Movement related type 1 θ is typically >7 Hz, where frequency range and amplitude increase with speed. This property has led to the theory that septo-hippocampal θ frequency translates movement displacement relative to coordinates in a cognitive spatial map, underpinning path integration (O’Keefe and Nadel, 1978; Buzsáki and Moser, 2013). In contrast, cholinergic dependent and alert immobility related type 2 θ, is typically <7 Hz and linked to arousal, anxiety or novelty at slow speeds or at rest (Vanderwolf, 1969; Kramis et al., 1975; O’Keefe and Nadel, 1978; Sainsbury et al., 1987; Kelemen et al., 2005; Jeewajee et al., 2008; Buzsáki and Moser, 2013). Yet beyond this distinction in hippocampal θ frequency, it remains unknown whether mPFC-HC θ subfrequencies have functional significance for spatial cognition (Niedecker et al., 2021) or vigilance (Kramis et al., 1975; Wells et al., 2013) during active avoidance.

We hypothesized that θ coherence between monosynaptically connected regions would be greater than polysynaptically connected regions, regardless of the phase of learning an active avoidance task on a rotating arena. In particular, we propose that indirect mPFC-dHC coherence would be statically lower than the directly connected mPFC-vHC. We further hypothesized that putative shifts in mPFC-HC circuit coherence frequency could indicate epochs of information organization and transfer within and between each region relative to avoidance, but primarily in the mPFC-vHC circuit.

To address this hypothesis, we bilaterally implanted EEG electrodes in the mPFC, vHC, and dHC and recorded local field potentials (LFPs) in each region during training in the spatial active avoidance task on a rotating arena. Contrary to our hypothesis, we found that during early training the dHC and mPFC showed higher levels of θ phase coordination than the monosynaptically connected vHC. After training, both hippocampal poles showed similar dynamic coherence patterns with the mPFC and there was likewise no indication of ipsilateral or contralateral projection effects. We also found a unique 8- to 9-Hz mPFC θ signal 3 s before avoidance and the multiplexing of coherence at θ subfrequencies that propagate throughout the mPFC-HC circuit. The findings further support a role for θ subfrequencies in the generation and transmission of qualitatively different types of information in the organization of spatial avoidance behavior (Adhikari et al., 2010; Padilla-Coreano et al., 2019).

## Materials and Methods

### Subjects

Male Sprague Dawley rats (Charles River; *n* = 5) were the subjects in this study. Rats were delivered at the age of postnatal day (P)50 and were singly housed thereafter. Rats were maintained on a 12/12 h light/dark cycle. Between P60 and P70, rats were implanted with a custom-made multisite implant, with EEG tetrodes in the mPFC, dHC, and vHC bilaterally.

### Implant

**Figure 1.**
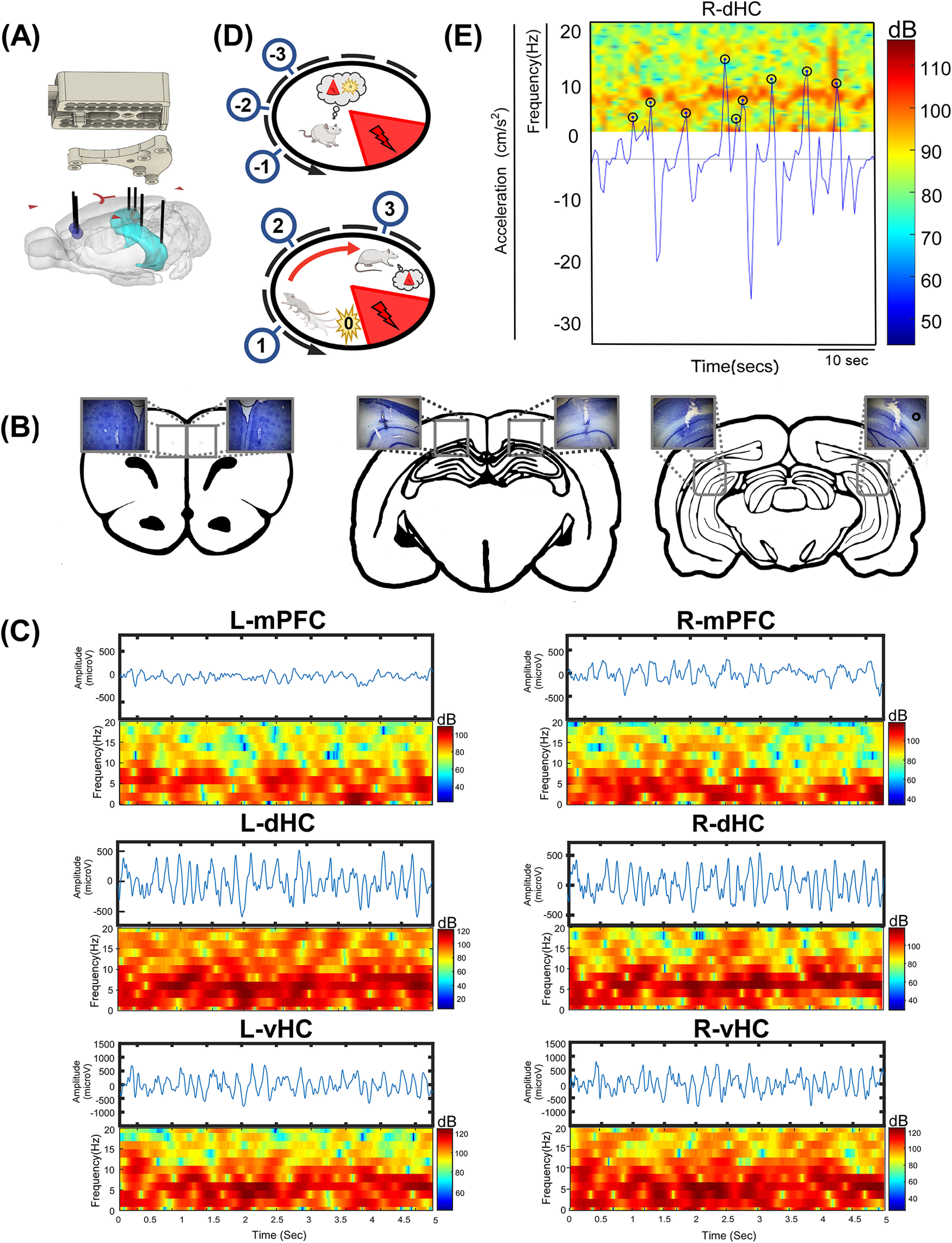
Experimental design. ***A***, Compact multisite implant design with electrode bundle track locations in bilateral hippocampus and mPFC. ***B***, Cresyl violet-stained brain tissue with representative EEG bundle tracks in each target region. ***C***, Representative 5-s filtered EEG trances in each region (0- to 55-Hz filter). Spectrograms of each trace (0–20 Hz). ***D***, Diagram of active avoidance on a rotating arena. Upon learning of the task, rats exhibit stereotyped avoidance behaviors, in which they passively approach the shock zone and eventually decide to run counter to the arena’s rotation and away from the shock zone (−3 to +3 s). ***E***, Representative spectrogram of an EEG channel in the right dorsal hippocampus for 50 s with an overlay of animal acceleration.

A custom 3D printed implant (Cellular and Molecular COBRE core facility) housing bundled EEG tetrodes (50 μm in diameter stainless steel electrodes; California Fine Wire) were cut to length so that implantation achieved a specified depth for the mPFC, dHC, and vHC bilaterally (Fig. 1*A–C*). Each EEG bundle was cut to length at an angle, producing a range of electrode contacts spanning ∼0.25 mm. Medial prefrontal electrodes were implanted in the prelimbic cortex (ML ±0.75 mm; AP +3.5 mm; DV −3.5 mm). Dorsal hippocampal tetrodes were implanted in the stratum lacunosum-moleculare (SLM)/stratum radiatum (SR) sublayers of CA1 (ML ±2.5 mm; AP −4.3 mm; DV −2.8 mm). Ventral HC tetrodes were similarly implanted in the SLM/SR sublayers (ML ±5.0 mm; AP −6.04 mm; DV −4.25 mm).

Electrodes were implanted ∼10 d before training. Rats were anesthetized with 4% inhaled isoflurane and placed in a stereotaxic frame. After the skull was exposed, four screws were inserted; two were placed anterior to bregma while the remaining two were placed above the cerebellum. Trephine holes for the electrodes were determined by coordinates relative to bregma. The right cerebellar skull screw was used for grounding while two wires placed over the left cerebellum were used as the reference electrodes. All implants were attached to the skull and skull screws via Palacos bone cement (Heraeus Medical). The incision sites were sutured and applied with topical antibiotics.

### Active avoidance

Rats were habituated to the active avoidance arena (no shocks) for 10 min on the stable arena. Subjects performed 8 active avoidance sessions each day for 2 d. Each session was 10 min in duration, with each session separated by ∼1 h. EEG recordings were performed throughout each session. Behavioral performance in the active avoidance task was determined by the number of shocks per session. Training sessions were characterized as “Early” when rats received >10 shocks per session and “Late,” when rats received <10 shocks per session. For each subject, a single Early and Late session was chosen for data analysis based on animal tracking and EEG quality.

During the active avoidance training sessions, rats were tethered to a recording cable. EEG signals were preamplified 1× at the headstage and passed through the cable to the signal amplifiers and computer interface (Neuralynx). The rat’s location in the arena was sampled at 60 Hz (Tracker, Biosignal) using a digital firewire camera which detected a light emitting diode (LED) placed near the animal’s head. Animal position and electrophysiology were synchronized offline using custom software. During active avoidance sessions, an additional LED was detected that was fixed to the rotating arena. The arena LED was then used as a reference for the animal LED, allowing for calculation of animal speed on the rotating platform. Instantaneous running speed was computed every 10 position samples (333 ms). Speed data were then linearly interpolated to fit the spectral data time range.

Custom code was used to analyze the behavioral data (Tracker, Biosignal). Sectors matching the shock zone were copied to the center of each quadrant and the proportion of the time in each center relative to the shock zone was measured [Target, Counterclockwise (CCW), Clockwise (CW), and Opposite (OPP)]. The number of shocks, entrances and the total path were also calculated.

### EEG acquisition

LFPs were sampled at 30.3 kHz and filtered at 1–9000 Hz. Offline subsampling was at 3000 Hz. LFPs were referenced against a 50-μm diameter stainless steel wire (California Fine Wire) placed on the brain surface over the cerebellum. LFPs were processed offline using custom software that used the MATLAB (v R2019A, MathWorks) signal processing toolbox “spectrogram’ function that returns the time-dependent Fourier transform (FFT) for a sequence using a sliding window (window = 1 s, overlap = 0.5 s for the analysis of the θ bandwidth; 5–12 Hz).

### EEG analysis

The purpose of this experiment was to evaluate cognitive demand, relative to movement during avoidance epochs, on the coordination between neural signals in both the dorsal and ventral hippocampus and the mPFC. Our primary measure of phase coordination of neural signals was the magnitude-squared coherence function (Kay, 1988; MathWorks *mscohere* function) using Welch’s overlapped averaged periodogram method (Welch, 1967; Rabiner and Gold, 1975) applied to LFP oscillations in each region. Coherence is a function of frequency with values between 0 and 1, indicating how well x corresponds to y at each frequency. The magnitude-squared coherence is a function of the power spectral densities, *P_xx_*(*f)* and *P_yy_*(*f)* , and the cross power spectral density, *P_xy_*(*f)* , of x and y:

$$Cxy(f)=\frac{|Pxy(f){|}^{2}}{Pxx\left(f\right)Pyy(f)}.$$

A value of 0 indicates that the two signals are perfectly uncorrelated, and a value of 1 indicates perfect correlation. Coherence is a bivariate measure, which means it considers only two signals simultaneously and is a nondirectional measure of signal coordination by frequency.

To quantify the effects of behavior and cognitive demand on signal coordination, we examined “avoidance arcs” during performance of the active avoidance task as in prior studies (Barry et al., 2020; Niedecker et al., 2021). During task performance, rats must remember to transition from a resting state, at least once every minute, avoid being rotated into the shock zone by running counter the direction of arena rotation. After initiation of avoidance, rats must then decide when to stop, lest they run into the opposite side of the shock zone. A cartoon depicting phases of rest, recall and increased sensorimotor processing demands during an avoidance arc are shown in Figure 1*D*. We organized the epochs into 3 s before to 3 s after the peak acceleration (0 s). Acceleration was calculated using finite differences in speed over time (MATLAB function *diff* for ΔSpeed./ΔTime). Peaks in the acceleration time series (MATLAB function *findpeaks*) were identified and each acceleration peak converted to a raster (Fig. 1*E*). The cross-correlation (MATLAB function *xcorr*) between this raster and the time-varying spectral power were then computed in each frequency band. Time series data relative to peak acceleration were then exported to process changes in coherence (see below) −3 to +3 s relative to peak acceleration.

Whole session θ coherence was averaged across subjects for both Early and Late training sessions. Coherence throughout the session was either averaged across the θ bandwidth (5–12 Hz) or binned by 0.49-Hz frequency intervals. We then divided samples of the Late active avoidance session into one second epochs relative to the animals’ peak acceleration; 3 s before and 3 s after the epoch in which the animal exhibited acceleration peaks during avoidance runs. During Late training sessions animals exhibit a stereotyped avoidance curve, remaining stationary until they are passively brought toward the invisible shock zone. Once the animals learn the shock zone location, rats will move toward the opposite side of the arena from the shock zone. This transition thus provides a useful signature for behaviorally relevant epochs for measuring coherence in relation to cognitive demand.

### Speed/θ properties

As in previous work (Mouchati et al., 2020), we analyzed the linear relationship between animal speed and either θ band properties and analyzed for putative differences between Early and Late active avoidance training sessions. Instantaneous running speed was computed every 10 position samples (333 ms). Speed data were then linearly interpolated to fit the spectral data time range. For each session, the Pearson correlation coefficients (*r*) were computed between speed and θ power or speed and θ frequency for all collected data points. The slope of speed-θ relationships was estimated by fitting a line in the data using a least squares linear regression (Polyfit.m function, MATLAB, MathWorks).

### Statistical analysis

Analysis results are presented as mean ± SE. The generalized linear model (GLM; SPSS) was used to evaluate behavioral metrics of active avoidance performance as a function of learning. The GLM is a conventional linear regression model that allows for a continuous response variable given a categorical predictor. The GLM was used to evaluate behavioral metrics such as number of shocks, entrances, total path, and quadrant dwell time.

The General Estimating Equations (GEE; SPSS 25.0), a class of regression marginal model, was used for repeated measure analysis of multivariable relationships between clustered signal property data or behavioral measures for individual animals sorted by experimental group. In each analysis, the most appropriate link function was used for the data distribution, i.e., γ or tweedie with log link models were used for non-normally distributed data. Details of relevant GEE outputs are available in extended data tables.

GEE was used to detect interactions between multiple variables pertaining to θ coherence. When comparing differences in overall θ coherence before and after rats acquired avoidance behavior (Early vs Late), training session interactions were tested relative to the degree of direct or indirect synaptic connectivity between the dHC, vHC, and mPFC (monosynaptic vs polysynaptic or ipsilateral vs contralateral). The three homotopic regional pairs (left-right mPFC, left-right dHC, left-right vHC) were then analyzed separately and examined for an interaction between training session and region. Each of these interaction tests were then performed with a third interaction variable, “frequency,” which binned θ coherence into 0.49-Hz frequency intervals.

Subsequent analyses then addressed θ coherence in the Late active avoidance session while subjects performed stereotyped avoidance arcs. GEE tested for interactions between the mono and polysynaptic variable for selected mPFC-HC comparisons and homotopic pairs by frequency and avoidance epoch.

Analyses were therefore conducted for the following: (1) coherence levels averaged across the 10-min session and θ bandwidth and examined for an interaction effect for training session and connectivity (Early vs Late × Mono vs Polysynaptic; Early vs Late × Ipsilateral vs Contralateral; Early vs Late × Homotopic pairs); (2) coherence levels averaged across the 10 min session and examined for an interaction effect for training session, connectivity and θ frequency (Early vs Late × Mono vs Polysynaptic × Frequency; Early vs Late × Ipsilateral vs Contralateral × Frequency; Early vs Late × Homotopic pairs × Frequency); (3) θ coherence levels in the Late training session at each avoidance epoch (−3 to +3 s from peak acceleration) averaged across θ bandwidth (mPFC-vHC vs mPFC-dHC × Epoch; Homotopic pairs × Epoch) or within θ bandwidth (mPFC-vHC vs mPFC-dHC × Epoch × Frequency; Homotopic pairs × Epoch × Frequency).

### Physiology of comparison regions

With simultaneous signal acquisition from bilateral mPFC, dHC, and vHC, we observed θ coherence dynamics across multiple pathways as subjects acquired and performed active avoidance behavior. Information transfer between brain region pairs was first categorized by the degree of synaptic connectivity, as it remains unclear whether this determines a ceiling for phase coherence. In this instance, the vHC has abundant monosynaptic afferents to the mPFC (Jay and Witter, 1991; Jay et al., 1996; Hoover and Vertes, 2007; Liu and Carter, 2018; Anastasiades and Carter, 2021) while the dHC interacts with the mPFC indirectly via bidirectional cortical intermediaries and thalamic nuclei (Thierry et al., 2000; Ito et al., 2015; Eichenbaum, 2017; Anastasiades and Carter, 2021). Similarly, the ipsilateral extent of the dorsal/ventral axis of the hippocampus is highly interconnected (Ishizuka et al., 1990; Lee et al., 2019) and their interdependence may functionally support spatial memory (Lee et al., 2019). In the rat, there are dorsal and ventral commissures, yet they are sparsely composed of reciprocally connected CA1 afferents. Instead, commissural fibers targeting CA1 largely originate from area CA3, with an additional association-commissural pathway in the dentate gyrus (Amaral et al., 2007; Andersen, 2007). In this experiment, the differences in monosynaptic (mPFC-vHC and dHC-vHC_IPSI_) and polysynaptic (mPFC-dHC and dHC-vHC_CONTRA_) θ coherence could therefore test whether the degree of connectivity would affect coherence levels as a function of learning during avoidance acquisition, or as hypothesized, that monosynaptic connections would always have a higher phase coherence, regardless of learning or cognitive demand.

We also analyzed coherence between left and right hemisphere homotopic pairs for each brain region (LmPFC-RmPFC, LdHC-RdHC, LvHC-RvHC). This was done to test for unique θ coherence signals in relation to avoidance, particularly the mPFC which has similar strength inter-hemispheric connections onto cortico-amygdalar and corticocortical neurons (Little and Carter, 2012; Lee et al., 2014; Anastasiades and Carter, 2021). This approach also allowed for comparison of unique signal frequencies (i.e., statistically narrow θ bandwidth subfrequencies) that occur within bistratified brain regions, and the degree to which these putative signals propagate between brain regions.

## Results

### Active avoidance acquisition

**Figure 2.**
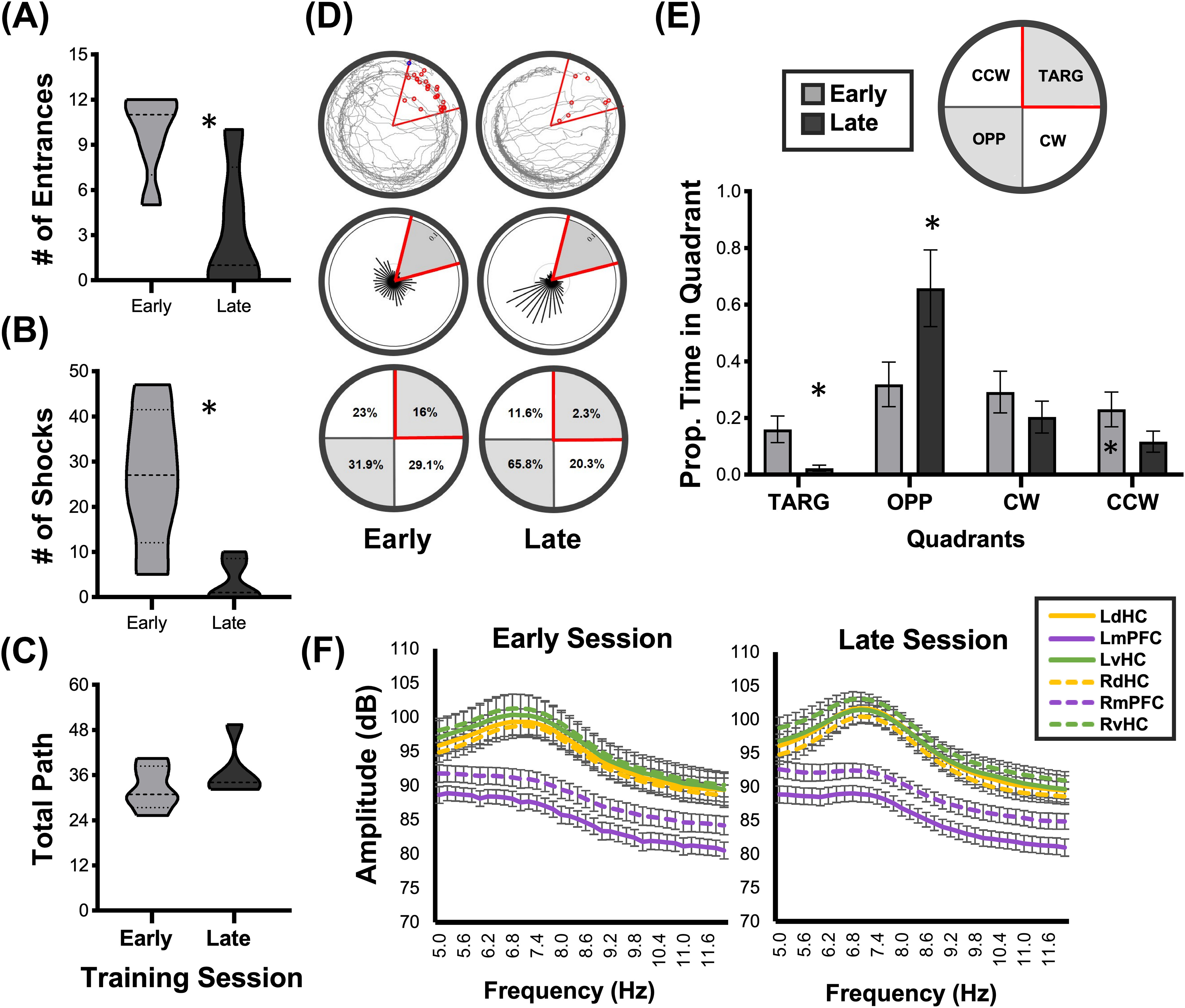
Active avoidance performance metric comparisons between Early and Late training sessions. ***A–C***, Truncated violin plots with median and upper/lower quartiles. ***A***, GLM found that rats received significantly fewer shocks in the Late training session compared with the Early training session (Early, 26.80 ± 2.315; Late, 4.80 ± 0.980; *p* < 0.00). ***B***, Subjects also had significantly fewer entrances into the shock-zone in the Late training sessions (Early, 9.80 ± 1.400; Late, 3.20 ± 0.800; *p* < 0.00). ***C***, GLM found no statistical mean difference between Early and Late training sessions with respect to total path (Early, 32.46 ± 2.378; Late, 36.76 ± 2.693; *p* = 0.230), which was an approximate measure of animal movement throughout the session. ***D***, Representative tracking traces (top) of an individual rat after Early (left) and Late (right) training sessions, with location density vectors (middle) to visualize the rat’s position probability. The proportion of dwell time (bottom) in each quadrant of the active avoidance arena was calculated for both Early and Late training sessions (TARG = shock zone quadrant). ***E***, Training session × Quadrant plot for the proportion of time in each quadrant zone. In the Late training sessions, rats demonstrated avoidance by decreasing their time in the shock zone (TARG) and increasing their time in the OPP quadrant. Asterisks above columns indicate significant difference (*p* < 0.005). ***F***, We found a significant main effect for training session × signal region × frequency interaction (*p* < 0.0001), which was largely driven by the smaller amplitude of all signals beyond the mid-θ peak of ∼ 7.4 Hz and the smaller amplitude of the mPFC. As peak hippocampal amplitude at ∼7.4 Hz is similar across learning conditions, dHC and vHC coherence differences as a function of learning cannot be accounted for by differences in signal amplitude.

Behavioral performance of the active avoidance task was primarily assessed by the number of shocks each rat (*N* = 5) received after the completion of a 10 min training session either Early or Late in training. The behavioral and electrophysiological data of one Early and Late training session from each subject was analyzed. Between Early and Late training phases, GLM analyses found a significant decrease in the number of shocks (Early = 26.80 ± 2.315; Late = 4.80 ± 0.980; Wald value = 60.20, *p* < 0.0001) and the number of shock zone entrances (Early = 9.80 ± 1.400; Late = 3.20 ± 0.800, *p* < 0.0001; Fig. 2*A*,*B*). However, there was no difference in total movement between Early and Late training sessions, as measured by total path distance (Early = 32.46 ± 2.378; Late = 36.76 ± 2.693, *p* = 0.230; Fig. 2*C*).

A further analysis revealed a dwell time interaction for Early and Late training sessions and arena quadrant: shock zone quadrant (TARG), as well as the quadrants OPP, CW, and CCW from the shock zone quadrant (Wald value = 57.42, *p* < 0.0001). Relative to the reference (Early CCW), dwell time was similar for all quadrants in the Early training session. In the Late training session, rats had significantly less dwell time in the shock zone quadrant (TARG) and significantly more dwell time in the OPP quadrant (Fig. 2*D*,*E*; Extended Data Table 2-1). This is consistent with the notion that the ideal avoidance strategy is to remain in the OPP quadrant from the shock zone (Patterson et al., 2017).

10.1523/ENEURO.0414-21.2022.tab2-1 Extended Data Table 2-1 Results of GLM Session × Percent time in Quadrant interactions. Percent time in CCW quadrant during Early training session as reference (0a). Download Table 2-1, DOCX file.

### Preacquisition versus postacquisition signal properties

As we used the MATLAB function *mscohere* to calculate coherence, which is a function of the power spectral densities, differences in signal amplitude in the ventral and dorsal hippocampus or between Early and Late training sessions could confound our results. We therefore used GEE to analyze for potential interactions between recording location, training session and frequency for signal amplitude. We found a significant main effect for training session × signal region × frequency interaction (Wald value = 2.5 × 10^7^, *p* < 0.0001; Fig. 2*F*; Extended Data Table 2-2), where the right vHC at the 7.4-Hz signal peak was used as a comparator. This significant interaction effect was largely driven by the smaller amplitude of all signals beyond the mid-θ peak of ∼7.4 Hz and the smaller amplitude of the mPFC in comparison to the HC (Extended Data Table 2-2), which has been shown previously (O’Neill et al., 2013). Importantly, the peak amplitude for all dHC and vHC signals at 7.4 Hz is similar across learning conditions (Extended Data Table 2-3). Therefore, putative coherence differences as a function of learning and synaptic connectivity between regions cannot be accounted for by differences in signal amplitude.

10.1523/ENEURO.0414-21.2022.tab2-2 Extended Data Table 2-2 Results of GEE Session × Region × Frequency interactions for signal amplitude. Peak signal at 7.4 Hz during the Late training session in the RvHC region was used as a reference (0a). Download Table 2-2, DOCX file.

10.1523/ENEURO.0414-21.2022.tab2-3 Extended Data Table 2-3 Results of GEE Session × Region × Frequency interactions for signal amplitude at peak 7.4-Hz signal only. Download Table 2-3, DOCX file.

### Preacquisition versus postacquisition coherence

Behavioral analyses suggest subjects acquired the avoidance response between the Early and Late training sessions. We hypothesized that levels of physiological connectivity between brain regions would supersede the influence of cognitive demand or learning, and the organization of avoidance behavior would therefore not coincide with detectable θ signal coordination changes between the mPFC and the dorsal or ventral hippocampus (dHC and vHC). This hypothesis was rejected as acquisition of active avoidance behavior led to significant coherence increases within and between the mPFC and hippocampal circuit. In addition, coherence between indirectly connected mPFC-dHC was always higher than the directly connected mPFC-vHC, particularly at faster frequencies.

**Figure 3.**
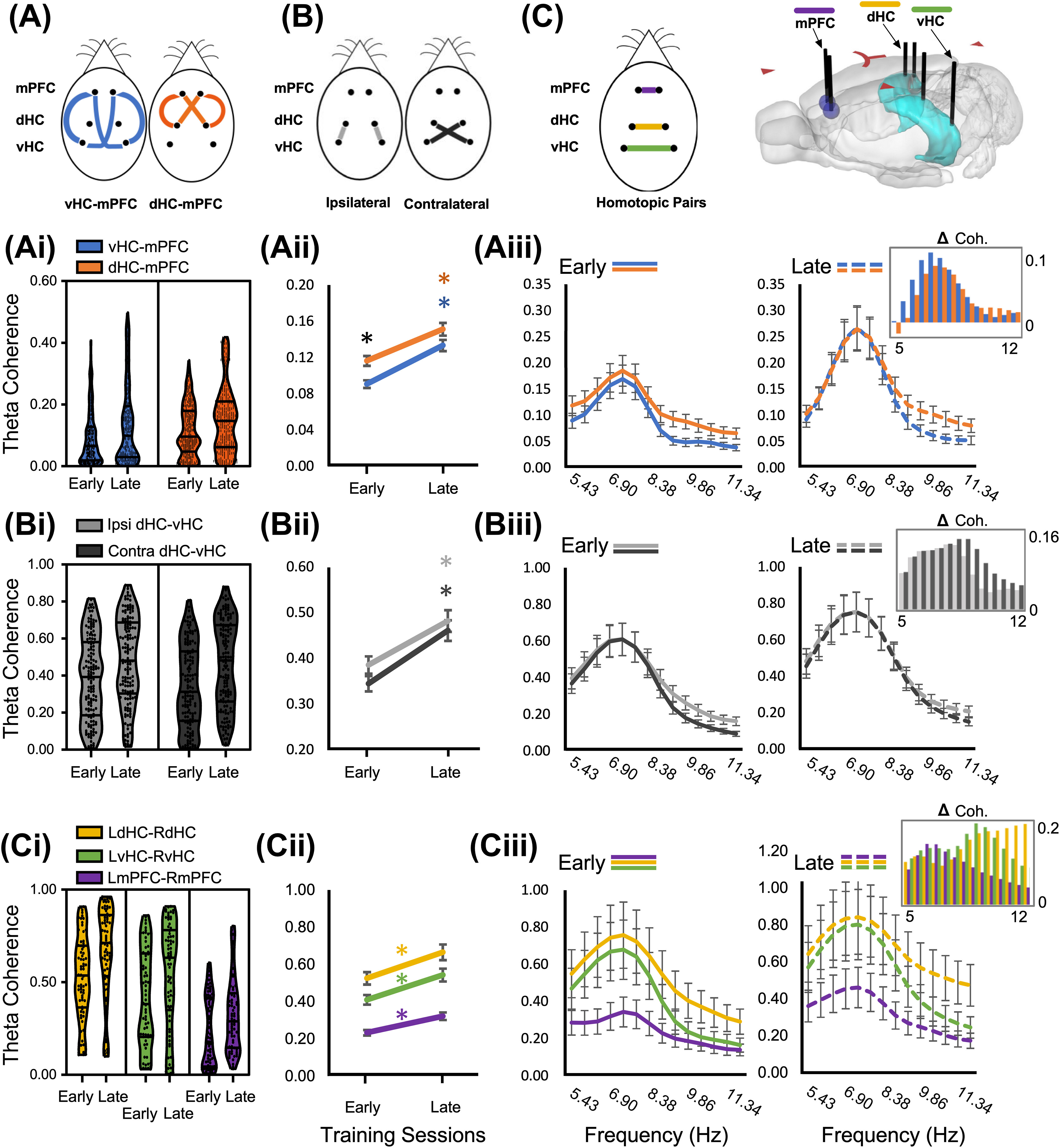
Whole session coherence within and across the θ frequency band (5–12 Hz) for comparison groups in Early and Late training sessions. ***A***, Diagram of comparison groups, ventral hippocampus to medial prefrontal cortex (vHC-mPFC) and dorsal hippocampus to medial prefrontal cortex (dHC-mPFC). ***B***, Diagram of comparison groups, ipsilateral dHC-vHC comparisons (ipsilateral) and contralateral dHC-vHC comparisons (contralateral). ***C***, Left, Diagram of homotopic comparison groups, mPFC, dHC, and vHC to the contralateral mPFC, dHC, and vHC, respectively (LmPFC-RmPFC, LdHC-RdHC, LvHC-RvHC). Right, Bilateral electrode configuration relative to each brain region. ***i***, Violin plots representing whole session θ coherence with individual coherence values at each frequency bin. ***Ai***, Ventral hippocampus to medial prefrontal cortex (vHC-mPFC) and dorsal hippocampus to medial prefrontal cortex (dHC-mPFC). ***Bi***, Ipsilateral dHC-vHC comparisons and contralateral dHC-vHC comparisons. ***Ci***, Homotopic comparison groups, mPFC, dHC, and vHC to the contralateral mPFC, dHC, and vHC, respectively (LmPFC-RmPFC, LdHC-RdHC, LvHC-RvHC). ***ii***, Session × Comparison group plots of θ coherence (5–12 Hz) across the frequency band. ***Aii***, In the Early training sessions, dHC-mPFC comparisons had greater θ coherence than vHC-mPFC comparisons. θ Coherence increased from Early to Late training sessions in both vHC-mPFC and dHC-mPFC comparisons. ***Bii***, θ Coherence increased between Early and Late sessions for both ipsilateral and contralateral comparisons, with no difference in θ coherence between each comparison within both sessions. ***Cii***, θ Coherence between LdHC-RdHC was greatest among comparison groups in both Early and Late training sessions. In each comparison group, θ coherence increased between Early and Late training sessions. ***iii***, Session × Comparison group × Frequency plots of coherence within the θ frequency band. All comparison groups of ***Aiii***, ***Biii***, ***Ciii*** exhibited a unimodal coherence distribution within the θ frequency band with a peak at 7.4 Hz. The increases in coherence among comparison groups (***Aiii***, ***Biii***) between Early and Late training sessions were largely accounted for within the bounds of this unimodal peak. ***Aiii***, In the Early training session, LdHC-RmPFC had greater coherence uniformly throughout the bandwidth with respect to vHC-mPFC coherence values. ***Biii***, Coherence values between ipsilateral and contralateral comparisons were similar in both Early and Late training sessions. ***Ciii***, As was observed in previous comparison groups, in both Early and Late sessions, homotopic comparison groups exhibited a unimodal coherence distribution within the θ frequency band with a peak at 7.4 Hz. However, increases in coherence were greater at higher θ frequencies among hippocampal homotopic comparisons while the prefrontal homotopic comparisons increased at lower frequencies.

We began our analysis by specifically addressing whether the degree of synaptic connectivity or phase of learning would differentially affect coherence levels in the mPFC-dHC versus the mPFC-vHC circuits (Fig. 3*A*). Violin plots of Early and Late mPFC-HC phase coherence across the θ frequency band are shown in Figure 3*Ai*. GEE found a significant training session × comparison group interaction between the mPFC-vHC and mPFC-dHC (Wald value = 56.86, *p* < 0.001; Fig. 3*Aii*). With Early mPFC-vHC as a reference, the mPFC-dHC had significantly greater θ coherence during both the Early (Wald value_EarlymPFC-dHC_ = 12.03, *p* = 0.001) and Late training sessions (Wald value_LatemPFC-dHC_ = 50.77, *p* < 0.001). θ Coherence between mPFC-vHC also increased from Early to Late (Wald value_mPFC-vHC_ = 28.86, *p* < 0.001; Extended Data Table 3-1). We then found a main effect for a session × synapse × frequency interaction, using the peak frequency of Late mPFC-vHC as a reference (Wald value = 55.86, *p* < 0.001; Fig. 3*Aiii*). While coherence peaks at 7.4 Hz were similar in the Early session mPFC-dHC and mPFC-vHC comparisons, mPFC-dHC had a uniformly greater coherence at all faster θ frequencies in comparison to mPFC-vHC (Extended Data Table 3-2). After the task was learned, Late coherence values in both comparison regions increased to identical peak frequencies (Fig. 3*Aiii*). However, between Early and Late sessions the increase in peak coherence was only significant in mPFC-vHC (Wald value = 4.20, *p* = 0.04) and not mPFC-dHC (Early vs Late, Wald value = 2.72, *p* = 0.099). Although the overall differences in Early session θ coherence between dHC and vHC were primarily driven by higher mPFC-dHC coherence at faster θ frequencies, the main change as a function of learning (Δ Coh) was greatest within the unimodal 7.4- to 8-Hz θ peak (Fig. 3*Aiii*, inset). Therefore, in contrast to the hypothesis, θ signal coordination between the polysynaptically connected mPFC-dHC is greater than the monosynaptic mPFC-vHC. This difference is more pronounced in early training and is driven by higher mPFC-dHC coherence at faster θ frequencies.

10.1523/ENEURO.0414-21.2022.tab3-1 Extended Data Table 3-1 Results of GEE Session × Comparison interactions for whole session θ coherence among dorsal hippocampus-medial prefrontal cortex (dHC-mPFC) and ventral hippocampus-medial prefrontal cortex (vHC-mPFC) regional comparisons in Early and Late training sessions. Ventral hippocampus-medial prefrontal cortex regional comparison in the Early training session as reference (0a). Download Table 3-1, DOCX file.

10.1523/ENEURO.0414-21.2022.tab3-2 Extended Data Table 3-2 Results of GEE Session × Comparison × Frequency interactions for whole session θ coherence among dorsal hippocampus-medial prefrontal cortex (dHC-mPFC) and ventral hippocampus-medial prefrontal cortex (vHC-mPFC) regional comparisons in Early and Late training sessions. Ventral hippocampus-medial prefrontal cortex regional comparison at 7.40 Hz in the Late training session as reference (0a). Download Table 3-2, DOCX file.

Previous studies have also found that ipsilateral communication on the septotemporal hippocampal axis was important for spatial processing (Lee et al., 2019), prompting us to compare ipsilateral and contralateral dHC-vHC coherence (Fig. 3*B*). GEE found a significant training session × synapse interaction (Wald value = 32.52, *p* < 0.001). Violin plots of Early and Late ipsilateral and contralateral dHC-vHC phase coherence across the θ frequency band are shown in Figure 3*Bi*. We used the Early ipsilateral coherence as a reference and discovered that both regional comparisons (dHC-vHC_IPSI_ and dHC-vHC_CONTRA_) once again had greater θ coherence during the Late session (Wald value_LateIPSI_ = 11.05, *p* = 0.001, Wald value_LateContra_ = 7.09, *p* = 0.008; Extended Data Table 3-3), but were not significantly different from each other in the Early session (Wald value_EarlyCONTRA_ = 2.78, *p* = 0.095; Fig. 3*Bii*). The ipsilateral and contralateral connections were therefore equal in coherence and had a similar coherence increase with task learning. We also found a significant session × synapse × frequency interaction (Wald value = 861.36, *p* < 0.001). With respect to the peak coherence at 7.4 Hz we found that both dHC-vHC_IPSI_ and dHC-vHC_CONTRA_ had significantly less coherence beyond 8.87 and 9.37 Hz in the Early and Late sessions, respectively (Fig. 3*Biii*; Extended Data Table 3-4). Ipsilateral versus contralateral coherence levels were similar and increased equally in both Early and Late sessions. Coherence again increased as a function of learning and the majority of this increase was accounted for within the ∼7.5-Hz unimodal peak (Fig. 3*Biii*, inset).

10.1523/ENEURO.0414-21.2022.tab3-3 Extended Data Table 3-3 Results of GEE Session × Synapses interactions for whole session θ coherence among ipsilateral and contralateral dorsal hippocampus-ventral hippocampus (dHC-vHC) regional comparisons in Early and Late training sessions. Ipsilateral dHC-vHC regional comparison in the Early training session as reference (0a). Download Table 3-3, DOCX file.

10.1523/ENEURO.0414-21.2022.tab3-4 Extended Data Table 3-4 Results of GEE Session × Synapses × Frequency interactions for whole session θ coherence among ipsilateral and contralateral dorsal hippocampus-ventral hippocampus (dHC-vHC) regional comparisons in Early and Late training sessions. Contralateral dHC-vHC regional comparison at 7.40 Hz in the Early training session as reference (0a). Download Table 3-4, DOCX file.

Finally, we analyzed homotopic bilateral pairs in each brain region of interest (Fig. 3*C*). This analysis examined θ coherence within each bilateral structure to find signals that might originate within regions of the mPFC-HC circuit, as the mPFC, dHC, and vHC may each be performing qualitatively different or cooperative spatial computation functions during avoidance (Moser and Moser, 1998; Lee et al., 2012; Cernotova et al., 2021a, b). The results of this analysis can then be compared with mPFC-dHC and mPFC-vHC results to examine the degree to which these signals propagate throughout the mPFC-HC circuit. GEE found a significant main effect in the training session x homotopic pair interaction (Wald value = 20.46, *p* < 0.001). Violin plots of Early and Late homotopic pairs from left and right dHC, vHC, and mPFC across the θ frequency band are shown in Figure 3*Ci*. Although mean θ coherence between pairs increased significantly between Early and Late training phases (Fig. 3*Cii*), there were striking regional differences within each session. θ Coherence for LmPFC-RmPFC was the lowest among homotopic pairs while LdHC-RdHC was higher than LvHC-RvHC. Relative to mid-range coherence levels, Late LvHC-RvHC was significantly different from all other comparisons except Early LdHC-RdHC (Extended Data Table 3-5). To further this point, we also made direct comparisons for each region and found a main effect of training phase (Wald value_LdHC-RdHC_ = 12.01, *p* = 0.001; Wald value_LvHC-RvHC_ = 8.09, *p* = 0.004; Wald value_LmPFC-RmPFC_ = 6.87, *p* = 0.009), where θ coherence increased significantly between Early and Late training sessions. We then performed a session × homotopic pair × frequency interaction with the peak frequency (7.4 Hz) of LmPFC-RmPFC in the Early session as a reference (Fig. 3*Ciii*; Extended Data Table 3-6). Whereas previous regional comparisons demonstrated the greatest Late phase training coherence increases within the bounds of the unimodal 7.4- to 8-Hz peak, LdHC-RdHC and LvHC-RvHC coherence increases were greatest at higher θ frequencies (Fig. 3*Ciii*, inset).

10.1523/ENEURO.0414-21.2022.tab3-5 Extended Data Table 3-5 Results of GEE Session × Comparison interactions for whole session θ coherence among homotopic regional comparisons in Early and Late training sessions. Ventral hippocampus homotopic regional comparison in the Late training session as reference (0a). Download Table 3-5, DOCX file.

10.1523/ENEURO.0414-21.2022.tab3-6 Extended Data Table 3-6 Results of GEE Session × Comparison × Frequency interactions for whole session θ coherence among homotopic regional comparisons in Early and Late training sessions. Medial prefrontal cortex homotopic regional comparison in the Early training session as reference (0a). Download Table 3-6, DOCX file.

Mean coherence is significantly higher in the dHC than the vHC or mPFC during both the Early and Late sessions and increases significantly in each homotopic pair as a function of learning. This result resembles previous work that showed coherence between hippocampi during active avoidance on the rotating arena was the most important determinant of cognitive outcome in a neonatal ventral hippocampal lesion (NVHL) of schizophrenia (Lee et al., 2012). Although peak coherence is at 7.4–8 Hz between all three homotopic pairs, the results indicate that higher mean LdHC-RdHC coherence across the session is because of more phase coordination at faster θ frequencies than in either the mPFC or vHC (Fig. 3*Ciii*). Yet, why was LmPFC-RmPFC coherence comparatively so much lower than intrahippocampal coherence? Since averaging over the whole session might diminish transient signals in relation to cognitive demand, we also conducted postacquisition coherence analyses in relation to avoidance epochs on a behaviorally relevant timescale with inherently different cognitive demands before, during, and after the avoidance runs (Barry et al., 2020).

### Preacquisition versus postacquisition effects were not influenced by speed

As changes in speed between Early and Late training sessions (i.e., more accurate avoidance behavior) could influence θ amplitude and thereby confound our acquisition effects for coherence, we analyzed speed and amplitude as a function of session. We found no significant main effect of speed between Early (mean = 4.85 ± 0.600 cm/s) and Late (mean = 6.25 ± 0.775 cm/s) training sessions (Wald value = 2.135, *p* = 0.144). Likewise, we analyzed the linear relationship between speed and θ amplitude and found no significant main effect for session for either the mPFC (mean_Early_ = 0.119 ± 0.0413; mean_Late_ = 0.0703 ± 0.0280, Wald Value = 1.196, *p* = 0.274), dHC (mean_Early_ = 0.302 ± 0.0529; mean_Late_ = 0.285 ± 0.0915, Wald Value = 0.023, *p* = 0.878), or vHC (mean_Early_ = 0.301 ± 0.0396; mean_Late_ = 0.250 ± 0.0812, Wald Value = 0.305, *p* = 0.581).

### Dynamic coherence on a behaviorally relevant timescale

**Figure 4.**
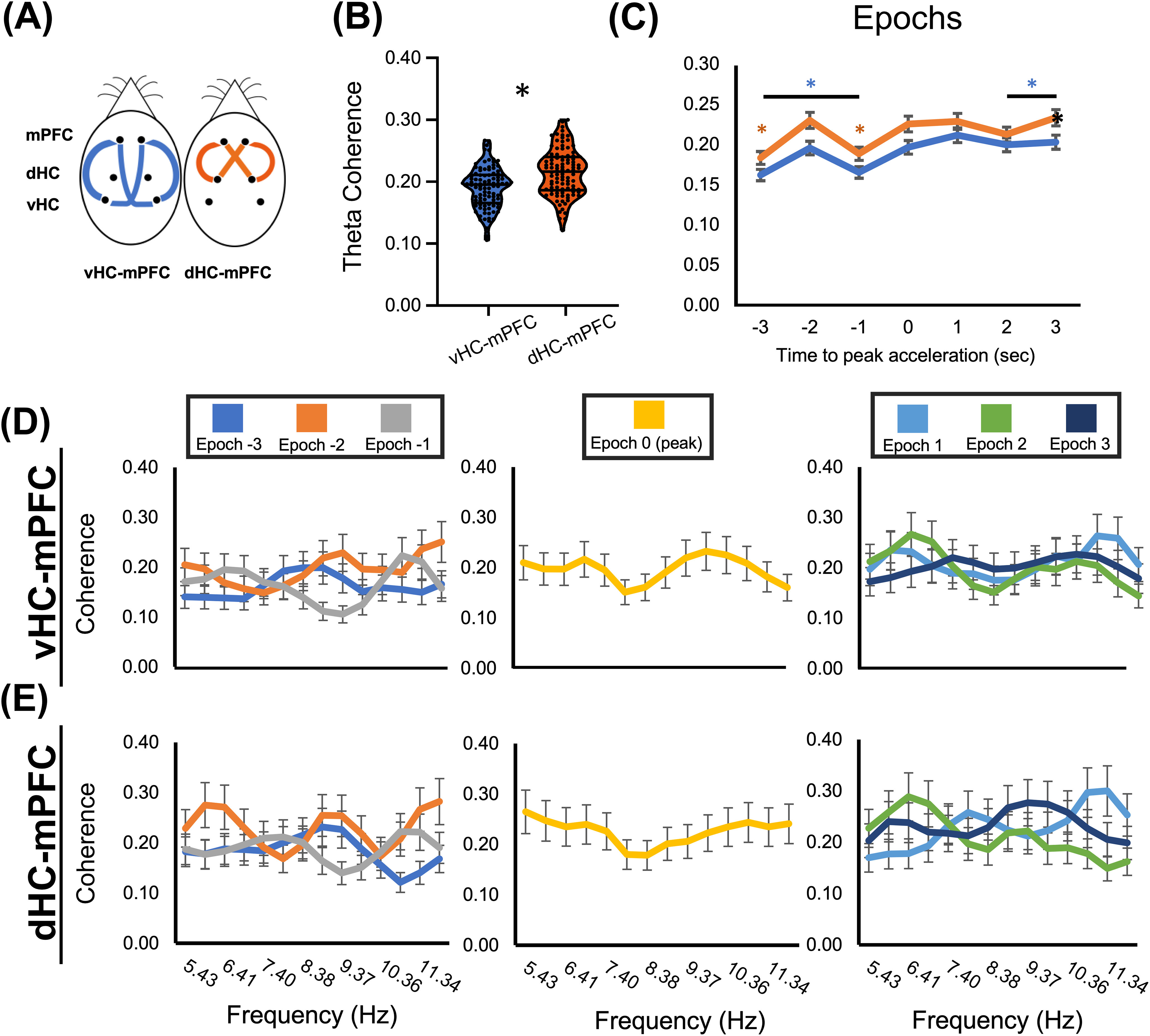
Dynamic changes in coherence within and across the θ frequency band during Late active avoidance: medial prefrontal cortex to hippocampus. ***A***, Comparison groups, mPFC-vHC and mPFC-dHC. ***B–E***, θ Coherence relative to peak acceleration; epochs −3 to −1 s before peak acceleration (left), at peak acceleration (middle), and +1 to +3 s after peak acceleration (right). ***B***, Violin plot with individual coherence values representing θ coherence across all epochs for vHC-mPFC and dHC-mPFC. θ Coherence for mPFC-dHC across epochs was significantly greater than mPFC-vHC. ***C***, Comparison × Epoch plots of θ coherence. Both mPFC-vHC and mPFC-dHC comparisons exhibited depressions in θ coherence at −3 and −1 s before peak acceleration at 0 s. ***D***, ***E***, Coherence within the θ band by epoch.

Whereas the survey of whole session changes in θ coherence from Early to Late training sessions indicate circuit communication as a function of spatial avoidance learning, it does not address moment-to-moment signal coordination dynamics in relation to transient cognitive demands. To study changes in signal coordination on a behaviorally relevant time scale during the postacquisition session, we binned regional coherence into 1-s time bins, ±3 s from peak acceleration (0 s) during the avoidance run. Using this approach, we first analyzed the overall θ coherence across all seven epochs for both mPFC-vHC and mPFC-dHC comparisons (Fig. 4*A*). As in whole session coherence analysis, mPFC-dHC dynamic θ exhibited a significant comparison region effect where mPFC-dHC exhibited greater coherence than mPFC-vHC across all avoidance epochs (Wald value = 26.04, *p* < 0.001). Violin plots for the coherence distribution across the θ band for each hippocampal pole and mPFC is shown in Figure 4*B*. θ Coherence was then analyzed in each epoch using the mPFC-dHC at epoch 3 s as a reference (Fig. 4*C*). GEE found a significant Epoch × Comparison Region interaction (Wald value = 93.41, *p* < 0.001) where mPFC-dHC exhibited significant depressions in θ coherence at epochs −3 and −1 s (epoch −3, Wald value = 15.54, *p* < 0.001; epoch −1, Wald value = 12.21, *p* < 0.001), while the mPFC-vHC had significantly less θ coherence in each epoch except for epoch 1 s (*p* > 0.05; Extended Data Table 4-1). Despite the differences in dHC and vHC dynamic coherence levels with mPFC, we note that the pattern in each avoidance epoch is very similar. In accordance with prior work, the most statistically relevant changes in coherence occur just before avoidance between −3 to −1 s (Niedecker et al., 2021).

10.1523/ENEURO.0414-21.2022.tab4-1 Extended Data Table 4-1 Results of GEE Comparison × Epoch interaction for time binned Late training session with ventral hippocampus-medial prefrontal cortex (vHC-mPFC) and dorsal hippocampus-medial prefrontal cortex (dHC-mPFC). Dorsal hippocampus-medial prefrontal cortex at epoch 3 as reference (0a). Download Table 4-1, DOCX file.

GEE also analyzed Epoch × Comparison Region × frequency interaction (Wald value = 265.47, *p* = 0.001) and found that although multiple coherence frequencies were active within the θ bandwidth between mPFC-dHC and mpFC-vHC (Fig. 4*D*,*E*), there was generally insufficient statistical variation to indicate any dominant or unique θ subfrequencies. However, both mPFC-dHC and mPFC-vHC exhibited multiple peaks at ∼ 6, 9, and 11 Hz, followed in epoch −1 by a decrease in coherence at ∼9 Hz (Fig. 4*D*,*E*; Extended Data Table 4-2). These multiple, co-existing frequencies in epoch −2 s underlie the significant increase in θ coherence at −2 s, while the decreased 9-Hz coherence at epoch −1 s likely underlies the drop in overall coherence before avoidance (Fig. 4*C*).

10.1523/ENEURO.0414-21.2022.tab4-2 Extended Data Table 4-2 Results of GEE Comparison × Epoch × Frequency interaction for time binned Late training session with ventral hippocampus-medial prefrontal cortex (vHC-mPFC) and dorsal hippocampus-medial prefrontal cortex (dHC-mPFC). Dorsal hippocampus-medial prefrontal cortex at epoch 3 s and 11.84 Hz as reference (0a) Download Table 4-2, DOCX file.

**Figure 5.**
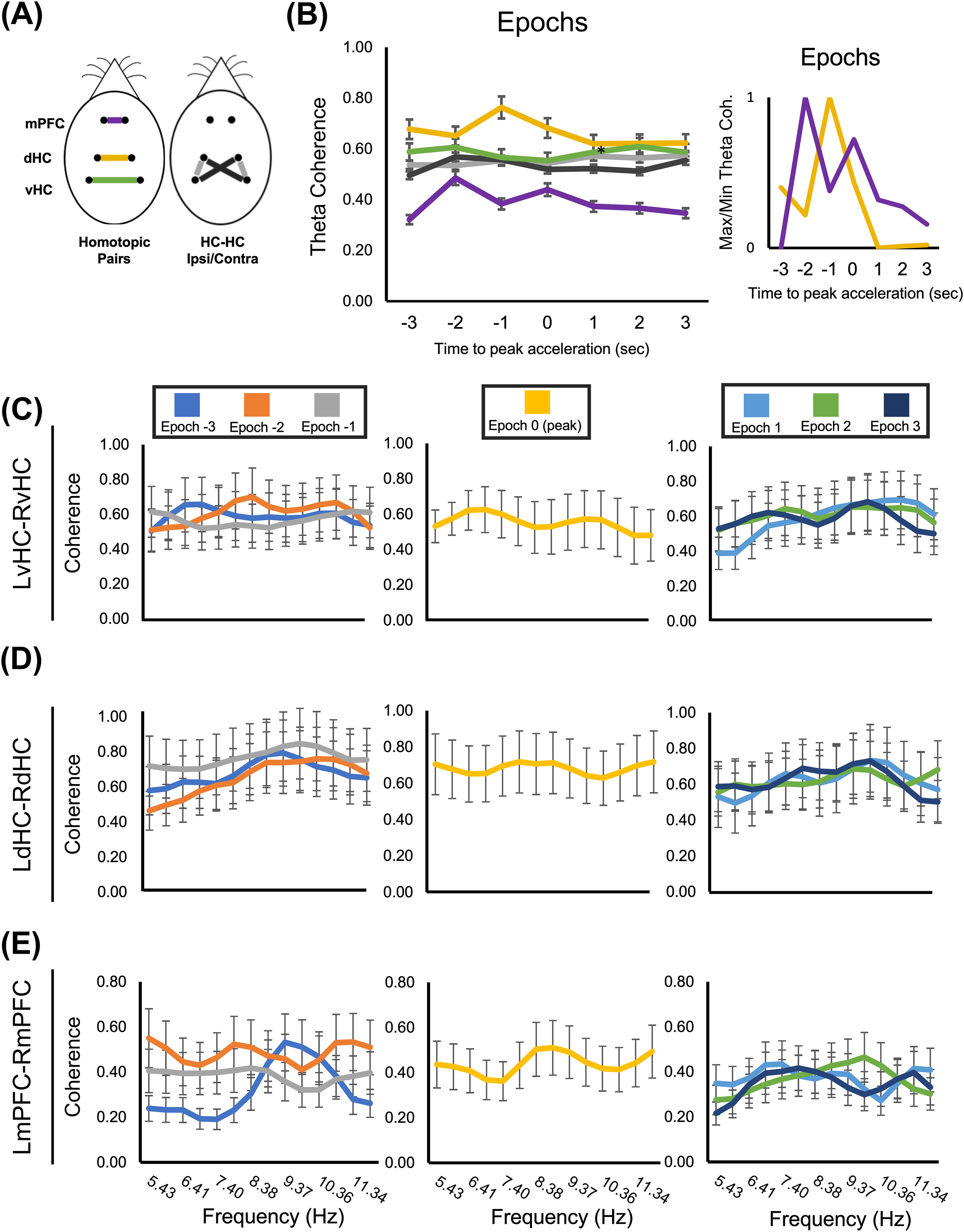
Dynamic changes in coherence within and across the θ frequency band during Late active avoidance: homotopic pairs. ***A***, Diagram of comparison groups, including homotopic pairs between the mPFC (purple), dHC (orange), and vHC (green), and ipsilateral versus contralateral dHC-vHC comparisons (gray and black, respectively). ***B***, θ Coherence relative to peak acceleration. vHC homotopic pairs and ipsilateral and contralateral dHC-vHC showed little variation in θ coherence between epochs. θ Coherence was greatest in the dHC homotopic group, which had a peak θ coherence at epoch −1 s. The mPFC homotopic group (orange) had two peaks, the first and largest at epoch −2 s, and then at epoch 0 s (peak acceleration). The order of these peaks was accentuated in the min/max normalization plot (right). ***C–E***, Coherence by frequency within the θ band for each homotopic pair. Coherence distributions for dHC and vHC homotopic pairs were statistically identical distributions, with no significant variation within the bandwidth. The mPFC homotopic group exhibited a statistically unique coherence signature at epoch −3, with a peak at 9.37 Hz.

Finally, we also analyzed θ coherence between epochs for left and right hemisphere homotopic pairs (mPFC, dHC, vHC) and ipsilateral versus contralateral dHC-vHC comparisons (Fig. 5*A*). Both ipsilateral and contralateral dHC-vHC and vHC-vHC regional comparison groups exhibited little statistical variation in θ coherence between epochs (Fig. 5*B*). Yet, there was a significant Epoch × Comparison Region main effect (Wald = 547.89, *p* < 0.001). With dHC-vHC_CONTRA_ at epoch 3 as a reference, neither dHC-vHC_CONTRA_, dHC-vHC_IPSI_, nor LvHC-RvHC exhibited epochs with significantly different θ coherence, with exception to epoch −3 dHC-vHC_CONTRA_ (Extended Data Table 5-1). Homotopic pair comparisons, on the other hand, revealed significant coherence dynamics as a function of avoidance epoch (Fig. 5*B*). For the dHC homotopic pair comparison, θ coherence was significantly greater in epochs −3–0, exhibiting a peak at epoch −1 s (Extended Data Table 5-1). The mPFC homotopic pair was significantly less coherent in each epoch but exhibited two coherence peaks at epoch −2 and epoch 0. The sequence of these peaks is demonstrated in the min/max coherence plot (Fig. 5*B*, right), displaying an interchange of peak θ coherence from the mPFC to dHC at epochs −2 to −1 s.

10.1523/ENEURO.0414-21.2022.tab5-1 Extended Data Table 5-1 Results of GEE Comparison × Epoch interaction for time binned Late training session with dorsal hippocampus, medial prefrontal cortex, and ventral hippocampus homotopics (dHC-dHC, mPFC-mPFC, vHC-vHC) and ipsilateral/contralateral dorsal hippocampus-ventral hippocampus (dHC-vHCIPSI, dHC-vHCCONTRA). Dorsal hippocampus-ventral hippocampus contralateral at epoch 3 s as reference (0a). Download Table 5-1, DOCX file.

Both homotopic dHC and vHC pairs did not display any notable dynamic coherence changes in frequency distribution within the θ bandwidth (Fig. 5*C*,*D*) as a function of avoidance epoch. However, we note that the highest dynamic coherence reported occurred at 9 Hz during the −1-s epoch between both dorsal hippocampi, explaining the LdHC-RdHC peak during this epoch (Fig. 5*B*). Yet, there was a significant Epoch × Comparison Region × frequency interaction (Wald value = 429.05, *p* < 0.001). In contrast to intrahippocampal signals, within bandwidth θ coherence for the mPFC homotopic pair revealed a coherence distribution at epoch −3 s, which was statistically distinct from subsequent epochs (Fig. 5*E*), characterized by a pronounced peak at ∼ 9 Hz and significantly lower coherence from 5 to 8 Hz (Extended Data Table 5-2). Importantly, this epoch was followed at −2 s by the multiple frequency state of 6-, 9-, and 11-Hz signals and at −1 s by a decrease in 9-Hz signal. We note that this same transition from “multiplexed” signal state at −2 s to a decrease in 9-Hz signal at −1 s, in both mPFC-dHC and mPFC-vHC coherence (Fig. 4*D*,*E*), was not present in either of the homotopic coherence pairs for vHC or dHC.

10.1523/ENEURO.0414-21.2022.tab5-2 Extended Data Table 5-2 Results of GEE Comparison × Epoch × Frequency interaction for time binned Late training session with dorsal hippocampus, medial prefrontal cortex, and ventral hippocampus homotopics (dHC-dHC, mPFC-mPFC, vHC-vHC). vHC-vHC during Epoch 3 s at 11.84 Hz as reference (0a). Download Table 5-2, DOCX file.

## Discussion

The hypothesis that coherence levels within and between circuits are determined by the level of direct synaptic connectivity, rather than phase of learning or cognitive demand, was rejected. Contrary to the hypothesis, coherence levels across θ bandwidth reliably increased as a function of learning in all comparison regions. Polysynaptically and monosynaptically connected regions were equally as coherent. This finding agrees with previous work that found increased θ coherence in cortico-striatal circuits as a consequence of learning an auditory discrimination task (Schulz et al., 2015).

Regarding our main question of mPFC coherence levels with the dHC versus vHC, the indirectly connected mPFC-dHC circuit was surprisingly more coherent than the directly connected mPFC-vHC. Detailed coherence frequency analysis revealed that this was because of a tendency for mPFC-dHC coordination to be more coherent at higher θ frequencies than mPFC-vHC. Equally as surprising was the result that, when averaged over long timescales, intra-mPFC coherence was significantly lower than intrahippocampal coherence. Analysis on a behaviorally relevant timescale revealed that intrahippocampal coherence is more persistently active and stable at mid to high θ frequencies while intra-mPFC coherence can be transiently high, particularly 3 s before avoidance. At −3 s, intra-mPFC coherence exhibited a clear 9-Hz signal, at −2 s, a “multiplexed” signal with 6-, 9-, and 11-Hz frequencies, and at −1 s, a suppressed 9-Hz signal. This pattern also exists in mPFC coherence with vHC and dHC, but not in intrahippocampal coherence. The data therefore suggest that in the seconds before avoidance the mPFC is transiently producing sub-θ signals that are coherent with both septotemporal hippocampal poles. Although mPFC-dHC coherence was higher than mPFC-vHC coherence at most epochs, the dynamics of the coherence frequency pattern across epochs in both hippocampal regions were similar.

Although our experiment was descriptive, it supports recent findings that suggest the necessity of mPFC-HC coordination in passive (Adhikari et al., 2010; Padilla-Coreano et al., 2016, 2019) and active spatial avoidance (Cernotova et al., 2021b) as well as the relevance of increased intrahippocampal coordination for successful spatial learning (Lee et al., 2012). Several earlier studies also investigated the necessity of the HC for active avoidance on the rotating arena by using tetrodotoxin to inactivate the HC. This may have also blocked fibers of passage and interfered with the direct mPFC-HC connection via the fimbria-fornix inactivation (Cimadevilla et al., 2001; Wesierska et al., 2005; Fenton et al., 2010). It is therefore possible that these studies also support the notion that coordination between the mPFC and HC is important for learning and recall during active avoidance on the rotating arena.

The results of another paper suggested that mPFC-HC coordination was also important for working memory. The approach of O’Neill et al. (2013) was similar to ours, where they looked at sampling and choice phases of a T-maze task rather than during phases of a spatial memory task. While the authors found that vHC was more active than dHC during working memory demands at the choice point, we found dHC to be more coordinated with mPFC than vHC during epochs of increased spatial memory demands during active avoidance. The high coherence between the left and right dHC, in comparison to other structures in the mPFC-HC circuit during active avoidance, has been shown previously (Lee et al., 2012). Yet this result is somewhat surprising given the high connectivity between the amygdala and the vHC (Swanson and Cowan, 1977), the role of the vHC in processing emotional behavior and stress (Henke, 1990), and the necessity of the amygdala for fear conditioning and avoidance (Vafaei et al., 2007). As the vHC has been shown to be less necessary than the dHC for spatial tasks (Moser and Moser, 1998), it may be that spatial memory demands during avoidance dictate high coherence between dorsal hippocampi and between the dHC and mPFC. However, the inactivation of both the mPFC and vHC, ipsilaterally or contralaterally, will interfere with memory retrieval during active avoidance (Cernotova et al., 2021b), but vHC inactivation can also decrease θ synchrony between mPFC and dHC (O’Neill et al., 2013). Taken together, these studies indicate that although the dHC and vHC have separate functions (Fanselow and Dong, 2010), they are both intricately connected to each other and signal coordination between hippocampal poles and the mPFC is necessary for memory. Ultimately, our results and those of O’Neil and colleagues support the idea of θ oscillations acting as a common traveling wave across the hippocampus and neocortex, coordinating disparate brain regions (Lubenov and Siapas, 2009; Muller et al., 2018; Hernández-Pérez et al., 2020).

The discovery of a 9-Hz intra-mPFC signal before avoidance, and a corresponding 8-Hz mPFC-dHC and mPFC-vHC coherence signal, is also supported by previous active avoidance studies. After early-life seizures, dynamic 8-Hz coherence at −3 s from peak acceleration was found to be a significant predictor of cognitive outcome in the active avoidance task (Niedecker et al., 2021). This suggests that this time period is both unique and reliable for the generation of a putative “avoidance signal.” The epoch at −3 s before avoidance has previously been shown to be associated with increased memory recollection of the shock zone location (Dvorak et al., 2018; Barry et al., 2020) while mPFC-CA1 θ synchrony at 8 Hz was found to be critical for the transmission of behaviorally relevant information during epochs of anxiety (Adhikari et al., 2010) or avoidance (Padilla-Coreano et al., 2016, 2019). Optogenetic inhibition of direct ventral hippocampal-mPFC projections reduces corresponding θ coordination and avoidance behavior (Padilla-Coreano et al., 2016), while sinusoidal optogenetic 8-Hz θ pacing of the mPFC increased avoidance behavior in comparison to 2-, 4-, or 20-Hz stimulation frequencies (Padilla-Coreano et al., 2019).

Several experiments point to a unique function for θ subfrequencies in mPFC-HC coordination during the generation of the avoidance response, yet the relevance and underlying mechanism for frequency specificity remains unclear. In our experiment, why signal coherence transitions to several θ frequencies between −3 and −2 s is equally enigmatic. However, the multiplexing of multiple slow frequencies in the ventral tegmental area, mPFC and HC during working memory has been shown previously (Fujisawa and Buzsáki, 2011). With regard to the 8- to 9-Hz mPFC-HC coherence signal, it has been suggested that this subfrequency is ideal for the temporal coordination of the mPFC-HC circuit (Adhikari et al., 2010; Spellman et al., 2015; Padilla-Coreano et al., 2019; Niedecker et al., 2021) as it facilitates the timing of presynaptic and postsynaptic action potentials and the optimal transfer of information between both brain regions. Optogenetic 8-Hz stimulation of the mPFC improves the temporal coordination of the mPFC-vHC circuit by increasing the probability of prefrontal neurons firing and synchronizing with their downstream vHC neuronal targets, promoting the subsequent organization and execution of the avoidance response (Padilla-Coreano et al., 2019). Yet, just as the efficacy of optogenetic stimulation in the septo-hippocampal circuit is affected by spatial cognitive demands (Mouchati et al., 2020), the efficacy of 8-Hz entrainment of the mPFC-vHC is dependent on the rat’s emotional state. The optogenetic 8-Hz mPFC stimulation preferentially enhanced synchrony of mPFC-vHC spiking activity and θ when the animals were in an anxious state on the open arms of an elevated plus maze (Padilla-Coreano et al., 2019). This is consistent with the hypothesis that the 8-Hz frequency creates an optimal window for mPFC responsiveness to vHC inputs. There may also be a role for serotonergic modulation in this interaction between behavioral state and circuit throughput during θ, as serotonin can gate vHC-mPFC neurotransmission during avoidance via presynaptic 5-HT_1B_ receptors (Kjaerby et al., 2016). Taken together, θ subfrequencies, in combination with specific neuromodulators, can significantly affect the efficacy and coordination of presynaptic and postsynaptic action potentials. This form of temporal coordination is critical for the transfer of situational or task-relevant information between “sender” and “receiver” components of a neural circuit (Fries, 2015; Barry et al., 2016; Niedecker et al., 2021).

The findings also support the notion that, as in sensory cortical processing, rhythms confined to a narrow frequency band may still have distinct signal properties and functions. Prior work has found that, during a 3-s stimulus sampling period, gustatory cortical LFPs can progress through a sequence of three distinct rhythms, all confined to the 7- to 12-Hz range (Tort et al., 2010). Changes in network rhythmicity during a task-related time period may ultimately reflect unfolding perceptual processes and the direction of information transfer in a given circuit.

To our knowledge, this experiment represents the first time that θ signal coordination between bilateral mPFC and the hippocampal poles has been studied during the acquisition of a spatial active avoidance task. The approach allowed for the analysis of synaptic connectivity in relation to dynamic coherence changes as a function of learning, and the degree to which unique θ frequency signals at behaviorally relevant timescales are coordinated both within and between the mPFC-HC circuit. The study revealed that the degree of circuit coordination is not predetermined by the level of synaptic connectivity but is heavily influenced by the level of cognitive demand and phase of learning.

The results reinforce the existence of θ frequency specificity in the transfer of relevant behavioral information between brain regions, emphasizing the need for understanding the cellular mechanisms that generate and govern these unique frequencies. Only in attaining these goals may we hope to effectively intervene in models of disease that attenuate learning and memory by recognizing and correcting neural signal discoordination underlying cognitive deficit.

## Synthesis

Reviewing Editor: Ifat Levy, Yale University School of Medicine Decisions are customarily a result of the Reviewing Editor and the peer reviewers coming together and discussing their recommendations until a consensus is reached. When revisions are invited, a fact-based synthesis statement explaining their decision and outlining what is needed to prepare a revision will be listed below. The following reviewer(s) agreed to reveal their identity: James Hyman.

The paper addresses an important topic: the theta synchronization between the mPFC and the hippocampus, which are critical for cognitive functions. The authors pose the hypothesis that the direct and indirect synaptic connectivity, rather than learning or cognitive demand, determine the theta coherence levels between these brain regions. To test this hypothesis, the authors recorded local field potential signals from mPFC, dHC, and vHC while rats were learning an active avoidance task on a rotating arena. The authors show that, contrary to the hypothesis, coherence levels increased as a function of learning. Moreover, the indirectly connected mPFC-dHC showed higher coherence during early stages of avoidance learning than the directly connected mPFC-vHC. Later in training, coherence first emerged bilaterally in the mPFC prior to movements and then was detectable between mPFC and HC as movements were initiated. These results suggest that the circuit coordination is not predetermined by the level of synaptic connectivity, but rather is heavily influenced by the level of cognitive demand and learning.

The paper addresses a question that has persisted in this area since the first publications showing theta band coordination between the mPFC and HC. While some studies have attempted to answer this question, it has largely been overlooked due to methodological challenges. The reported findings are very interesting and provide strong evidence for the role of theta sub frequencies in the 8-9 Hz in the mPFC in generating coherence with the hippocampus during the avoidance learning behavior.

There are, however, a few issues that the authors need to address:

- Theta synchrony between mPFC, dHC, and vHC in spatial working memory has been previously examined. A previous paper (O’Neill et al., 2013, J Neuro) has looked at the mPFC, dHC, and vHC theta coordination in mice performing the T-maze delayed non-match-to-sample test. While there are similar findings from the two studies, there is also a major difference. O’Neill et al. (2013) show that the hippocampal-prefrontal synchrony during spatial working memory relies primarily on the theta activity in the vHC while the authors here show that the mPFC synchrony in the spatial active avoidance task is coherent with the theta activity in the dHC. The authors should refer to the O’Neill et. al. 2013 paper and discuss the differences in findings. Having said that, the coherence shown in the current manuscript on behaviorally relevant timescales is novel and interesting.

- To get at causal direction, the authors should perform Granger causality or time-lagged correlations or another similar analysis.

- The authors present filtered EEG traces in Fig. 1C, which shows that the amplitudes of the signals in mPFC, dHC, and vHC are clearly different. The authors mention in the Methods that the ’mscohere’ function in Matlab was used to calculate the coherence. The ’mscohere’ is a function of the power spectral densities. The power spectral densities will be affected by the amplitudes of the signals. Can the stronger coherence between the mPFC-dHC be due to the stronger amplitudes of the dHC signals than the vHC signals (shown in Fig. 1C)? The authors need to show the power spectral densities from all regions (mPFC, dHC, vHC), and if there are spectral power differences across the regions, state how they normalized the differences before calculating the coherence between the regions.

- In Fig. 3A, monosynaptic is a combination of mPFC-vHC and ipsilateral dHC-vHC and polysynaptic is a combination of mPFC-dHC and contralateral dHC and vHC. However, this is a bit confusing, because the mPFC-vHC is also referred to as monosynaptic and the mPFC-dHC is also referred to as polysynaptic connections. If the hypothesis is to test whether the coherence is higher in the directly connected mPFC-vHC vs. indirectly connected mPFC-dHC, the main finding should be the current Fig. 4A-C, which should come first. The current Fig. 3A-C should be removed as it does not seem to contribute important findings but just create confusion. Rather, Fig. 3-4 can be combined, with the order as follow: Fig. 3A-C showing mPFC-vHC vs. mPFC-dHC, Fig. 3D-F showing ipsi vs contra, and Fig. 3G-I showing the homotopic pairs so that it will be easier to visualize all the comparisons in one figure.

- Figures should be more informative - they should be modified to present data points through swarmplots or violin or raincloud plots. Also, the use of gridlines is distracting, and graphs should have y-axis lines.

- Figure 7 is not clear and does not convey the message the authors ascribe to it. The authors should identify a within theta freq range of interest and then plot the data for the 3 coherence comparisons over time on a single plot. This will let the readers see the emergence of the 8-9Hz in mPFC and then the subsequent coherence between the mPFC and HC.

- Theta power could potentially account for the differences observed in coherence and this analysis should be in the Main text and not the Supplemental.
