# Supplementary material for: Dynamic θ Frequency Coordination within and between the Prefrontal Cortex-Hippocampus Circuit during Learning of a Spatial Avoidance Task
Source: eNeuro. 2022 Apr 21;9(2):ENEURO.0414-21.2022. doi: 10.1523/ENEURO.0414-21.2022 (PMC9034755; doi:10.1523/ENEURO.0414-21.2022)
Supplement: Extended Data Table 2-1 — Results of GLM Session × Percent time in Quadrant interactions. Percent time in CCW quadrant during Early training session as reference (0a). Download Table 2-1, DOCX file. [file enu-eN-NWR-0414-21-s03.docx]

Extended Table 2-1 Results of GLM Session × Percent Time in Quadrant interactions. Percent time in CCW quadrant during Early training session as reference (0a).

Session × Percent Time in Quadrant Wald Value Sig.

[Session=Late] * [Zone=pTimeTARG] 17.888 0.000 *

[Session=Late] * [Zone=pTimeOPP] 9.674 0.002 *

[Session=Late] * [Zone=pTimeCW] 0.105 0.746

[Session=Late] * [Zone=pTimeCCW] 2.702 0.100

[Session=Early] * [Zone=pTimeTARG] 0.848 0.357

[Session=Early] * [Zone=pTimeOPP] 0.800 0.371

[Session=Early] * [Zone=pTimeCW] 0.410 0.522

[Session=Early] * [Zone=pTimeCCW] 0a

Extended Table 2-2. Results of GEE Session × Region × Frequency interactions for signal amplitude. Peak signal at 7.4 Hz during the Late training session in the RvHC region was used as a reference (0a).

Session × Region × Frequency Wald Value Sig.

| [Session=Early] * [Region=LdHC] * [Frequency=5.0] | 6.579 | 0.010 | * |
| --- | --- | --- | --- |
| [Session=Early] * [Region=LdHC] * [Frequency=5.2] | 5.854 | 0.016 | * |
| [Session=Early] * [Region=LdHC] * [Frequency=5.4] | 5.076 | 0.024 | * |
| [Session=Early] * [Region=LdHC] * [Frequency=5.6] | 4.465 | 0.035 | * |
| [Session=Early] * [Region=LdHC] * [Frequency=5.8] | 3.755 | 0.053 |  |
| [Session=Early] * [Region=LdHC] * [Frequency=6.0] | 3.128 | 0.077 |  |
| [Session=Early] * [Region=LdHC] * [Frequency=6.2] | 2.459 | 0.117 |  |
| [Session=Early] * [Region=LdHC] * [Frequency=6.4] | 1.996 | 0.158 |  |
| [Session=Early] * [Region=LdHC] * [Frequency=6.6] | 1.696 | 0.193 |  |
| [Session=Early] * [Region=LdHC] * [Frequency=6.8] | 1.494 | 0.222 |  |
| [Session=Early] * [Region=LdHC] * [Frequency=7.0] | 1.520 | 0.218 |  |
| [Session=Early] * [Region=LdHC] * [Frequency=7.2] | 1.522 | 0.217 |  |
| [Session=Early] * [Region=LdHC] * [Frequency=7.4] | 1.744 | 0.187 |  |
| [Session=Early] * [Region=LdHC] * [Frequency=7.6] | 2.161 | 0.142 |  |
| [Session=Early] * [Region=LdHC] * [Frequency=7.8] | 2.824 | 0.093 |  |
| [Session=Early] * [Region=LdHC] * [Frequency=8.0] | 3.934 | 0.047 | * |
| [Session=Early] * [Region=LdHC] * [Frequency=8.2] | 5.141 | 0.023 | * |
| [Session=Early] * [Region=LdHC] * [Frequency=8.4] | 6.582 | 0.010 | * |
| [Session=Early] * [Region=LdHC] * [Frequency=8.6] | 8.331 | 0.004 | * |
| [Session=Early] * [Region=LdHC] * [Frequency=8.8] | 10.238 | 0.001 | * |
| [Session=Early] * [Region=LdHC] * [Frequency=9.0] | 12.114 | 0.001 | * |
| [Session=Early] * [Region=LdHC] * [Frequency=9.2] | 13.773 | 0.000 | * |
| [Session=Early] * [Region=LdHC] * [Frequency=9.4] | 15.396 | 0.000 | * |
| [Session=Early] * [Region=LdHC] * [Frequency=9.6] | 16.835 | 0.000 | * |
| [Session=Early] * [Region=LdHC] * [Frequency=9.8] | 18.336 | 0.000 | * |
| [Session=Early] * [Region=LdHC] * [Frequency=10.0] | 19.931 | 0.000 | * |
| [Session=Early] * [Region=LdHC] * [Frequency=10.2] | 20.995 | 0.000 | * |
| [Session=Early] * [Region=LdHC] * [Frequency=10.4] | 21.894 | 0.000 | * |
| [Session=Early] * [Region=LdHC] * [Frequency=10.6] | 22.956 | 0.000 | * |
| [Session=Early] * [Region=LdHC] * [Frequency=10.8] | 23.890 | 0.000 | * |
| [Session=Early] * [Region=LdHC] * [Frequency=11.0] | 25.090 | 0.000 | * |
| [Session=Early] * [Region=LdHC] * [Frequency=11.2] | 25.506 | 0.000 | * |
| [Session=Early] * [Region=LdHC] * [Frequency=11.4] | 26.227 | 0.000 | * |
| [Session=Early] * [Region=LdHC] * [Frequency=11.6] | 26.633 | 0.000 | * |
| [Session=Early] * [Region=LdHC] * [Frequency=11.8] | 26.869 | 0.000 | * |
| [Session=Early] * [Region=LdHC] * [Frequency=12.0] | 27.171 | 0.000 | * |
| [Session=Early] * [Region=LPFC] * [Frequency=5.0] | 30.526 | 0.000 | * |
| [Session=Early] * [Region=LPFC] * [Frequency=5.2] | 29.407 | 0.000 | * |
| [Session=Early] * [Region=LPFC] * [Frequency=5.4] | 29.836 | 0.000 | * |
| [Session=Early] * [Region=LPFC] * [Frequency=5.6] | 30.334 | 0.000 | * |
| [Session=Early] * [Region=LPFC] * [Frequency=5.8] | 30.825 | 0.000 | * |
| [Session=Early] * [Region=LPFC] * [Frequency=6.0] | 33.041 | 0.000 | * |
| [Session=Early] * [Region=LPFC] * [Frequency=6.2] | 31.609 | 0.000 | * |
| [Session=Early] * [Region=LPFC] * [Frequency=6.4] | 31.801 | 0.000 | * |
| [Session=Early] * [Region=LPFC] * [Frequency=6.6] | 32.327 | 0.000 | * |
| [Session=Early] * [Region=LPFC] * [Frequency=6.8] | 33.273 | 0.000 | * |
| [Session=Early] * [Region=LPFC] * [Frequency=7.0] | 35.887 | 0.000 | * |
| [Session=Early] * [Region=LPFC] * [Frequency=7.2] | 35.306 | 0.000 | * |
| [Session=Early] * [Region=LPFC] * [Frequency=7.4] | 36.573 | 0.000 | * |
| [Session=Early] * [Region=LPFC] * [Frequency=7.6] | 38.677 | 0.000 | * |
| [Session=Early] * [Region=LPFC] * [Frequency=7.8] | 41.326 | 0.000 | * |
| [Session=Early] * [Region=LPFC] * [Frequency=8.0] | 46.105 | 0.000 | * |
| [Session=Early] * [Region=LPFC] * [Frequency=8.2] | 47.100 | 0.000 | * |
| [Session=Early] * [Region=LPFC] * [Frequency=8.4] | 49.691 | 0.000 | * |
| [Session=Early] * [Region=LPFC] * [Frequency=8.6] | 53.039 | 0.000 | * |
| [Session=Early] * [Region=LPFC] * [Frequency=8.8] | 56.626 | 0.000 | * |
| [Session=Early] * [Region=LPFC] * [Frequency=9.0] | 61.590 | 0.000 | * |
| [Session=Early] * [Region=LPFC] * [Frequency=9.2] | 61.860 | 0.000 | * |
| [Session=Early] * [Region=LPFC] * [Frequency=9.4] | 64.126 | 0.000 | * |
| [Session=Early] * [Region=LPFC] * [Frequency=9.6] | 66.507 | 0.000 | * |
| [Session=Early] * [Region=LPFC] * [Frequency=9.8] | 68.785 | 0.000 | * |
| [Session=Early] * [Region=LPFC] * [Frequency=10.0] | 73.419 | 0.000 | * |
| [Session=Early] * [Region=LPFC] * [Frequency=10.2] | 72.430 | 0.000 | * |
| [Session=Early] * [Region=LPFC] * [Frequency=10.4] | 73.107 | 0.000 | * |
| [Session=Early] * [Region=LPFC] * [Frequency=10.6] | 74.193 | 0.000 | * |
| [Session=Early] * [Region=LPFC] * [Frequency=10.8] | 75.100 | 0.000 | * |
| [Session=Early] * [Region=LPFC] * [Frequency=11.0] | 78.804 | 0.000 | * |
| [Session=Early] * [Region=LPFC] * [Frequency=11.2] | 77.737 | 0.000 | * |
| [Session=Early] * [Region=LPFC] * [Frequency=11.4] | 78.799 | 0.000 | * |
| [Session=Early] * [Region=LPFC] * [Frequency=11.6] | 79.854 | 0.000 | * |
| [Session=Early] * [Region=LPFC] * [Frequency=11.8] | 80.799 | 0.000 | * |
| [Session=Early] * [Region=LPFC] * [Frequency=12.0] | 83.861 | 0.000 | * |
| [Session=Early] * [Region=LvHC] * [Frequency=5.0] | 4.497 | 0.034 | * |
| [Session=Early] * [Region=LvHC] * [Frequency=5.2] | 3.812 | 0.051 |  |
| [Session=Early] * [Region=LvHC] * [Frequency=5.4] | 3.217 | 0.073 |  |
| [Session=Early] * [Region=LvHC] * [Frequency=5.6] | 2.727 | 0.099 |  |
| [Session=Early] * [Region=LvHC] * [Frequency=5.8] | 2.242 | 0.134 |  |
| [Session=Early] * [Region=LvHC] * [Frequency=6.0] | 1.855 | 0.173 |  |
| [Session=Early] * [Region=LvHC] * [Frequency=6.2] | 1.325 | 0.250 |  |
| [Session=Early] * [Region=LvHC] * [Frequency=6.4] | 1.009 | 0.315 |  |
| [Session=Early] * [Region=LvHC] * [Frequency=6.6] | 0.822 | 0.365 |  |
| [Session=Early] * [Region=LvHC] * [Frequency=6.8] | 0.701 | 0.402 |  |
| [Session=Early] * [Region=LvHC] * [Frequency=7.0] | 0.746 | 0.388 |  |
| [Session=Early] * [Region=LvHC] * [Frequency=7.2] | 0.759 | 0.384 |  |
| [Session=Early] * [Region=LvHC] * [Frequency=7.4] | 0.943 | 0.331 |  |
| [Session=Early] * [Region=LvHC] * [Frequency=7.6] | 1.300 | 0.254 |  |
| [Session=Early] * [Region=LvHC] * [Frequency=7.8] | 1.863 | 0.172 |  |
| [Session=Early] * [Region=LvHC] * [Frequency=8.0] | 2.725 | 0.099 |  |
| [Session=Early] * [Region=LvHC] * [Frequency=8.2] | 3.620 | 0.057 |  |
| [Session=Early] * [Region=LvHC] * [Frequency=8.4] | 4.888 | 0.027 | * |
| [Session=Early] * [Region=LvHC] * [Frequency=8.6] | 6.469 | 0.011 | * |
| [Session=Early] * [Region=LvHC] * [Frequency=8.8] | 8.301 | 0.004 | * |
| [Session=Early] * [Region=LvHC] * [Frequency=9.0] | 10.426 | 0.001 | * |
| [Session=Early] * [Region=LvHC] * [Frequency=9.2] | 12.022 | 0.001 | * |
| [Session=Early] * [Region=LvHC] * [Frequency=9.4] | 13.444 | 0.000 | * |
| [Session=Early] * [Region=LvHC] * [Frequency=9.6] | 14.848 | 0.000 | * |
| [Session=Early] * [Region=LvHC] * [Frequency=9.8] | 16.050 | 0.000 | * |
| [Session=Early] * [Region=LvHC] * [Frequency=10.0] | 17.489 | 0.000 | * |
| [Session=Early] * [Region=LvHC] * [Frequency=10.2] | 18.274 | 0.000 | * |
| [Session=Early] * [Region=LvHC] * [Frequency=10.4] | 19.404 | 0.000 | * |
| [Session=Early] * [Region=LvHC] * [Frequency=10.6] | 20.590 | 0.000 | * |
| [Session=Early] * [Region=LvHC] * [Frequency=10.8] | 21.640 | 0.000 | * |
| [Session=Early] * [Region=LvHC] * [Frequency=11.0] | 23.043 | 0.000 | * |
| [Session=Early] * [Region=LvHC] * [Frequency=11.2] | 23.734 | 0.000 | * |
| [Session=Early] * [Region=LvHC] * [Frequency=11.4] | 24.202 | 0.000 | * |
| [Session=Early] * [Region=LvHC] * [Frequency=11.6] | 25.041 | 0.000 | * |
| [Session=Early] * [Region=LvHC] * [Frequency=11.8] | 25.834 | 0.000 | * |
| [Session=Early] * [Region=LvHC] * [Frequency=12.0] | 26.974 | 0.000 | * |
| [Session=Early] * [Region=RdHC] * [Frequency=5.0] | 8.777 | 0.003 | * |
| [Session=Early] * [Region=RdHC] * [Frequency=5.2] | 8.031 | 0.005 | * |
| [Session=Early] * [Region=RdHC] * [Frequency=5.4] | 7.106 | 0.008 | * |
| [Session=Early] * [Region=RdHC] * [Frequency=5.6] | 6.289 | 0.012 | * |
| [Session=Early] * [Region=RdHC] * [Frequency=5.8] | 5.348 | 0.021 | * |
| [Session=Early] * [Region=RdHC] * [Frequency=6.0] | 4.362 | 0.037 | * |
| [Session=Early] * [Region=RdHC] * [Frequency=6.2] | 3.554 | 0.059 |  |
| [Session=Early] * [Region=RdHC] * [Frequency=6.4] | 2.886 | 0.089 |  |
| [Session=Early] * [Region=RdHC] * [Frequency=6.6] | 2.478 | 0.115 |  |
| [Session=Early] * [Region=RdHC] * [Frequency=6.8] | 2.228 | 0.136 |  |
| [Session=Early] * [Region=RdHC] * [Frequency=7.0] | 2.096 | 0.148 |  |
| [Session=Early] * [Region=RdHC] * [Frequency=7.2] | 2.136 | 0.144 |  |
| [Session=Early] * [Region=RdHC] * [Frequency=7.4] | 2.382 | 0.123 |  |
| [Session=Early] * [Region=RdHC] * [Frequency=7.6] | 2.845 | 0.092 |  |
| [Session=Early] * [Region=RdHC] * [Frequency=7.8] | 3.670 | 0.055 |  |
| [Session=Early] * [Region=RdHC] * [Frequency=8.0] | 4.918 | 0.027 | * |
| [Session=Early] * [Region=RdHC] * [Frequency=8.2] | 6.315 | 0.012 | * |
| [Session=Early] * [Region=RdHC] * [Frequency=8.4] | 8.006 | 0.005 | * |
| [Session=Early] * [Region=RdHC] * [Frequency=8.6] | 9.945 | 0.002 | * |
| [Session=Early] * [Region=RdHC] * [Frequency=8.8] | 11.960 | 0.001 | * |
| [Session=Early] * [Region=RdHC] * [Frequency=9.0] | 14.098 | 0.000 | * |
| [Session=Early] * [Region=RdHC] * [Frequency=9.2] | 15.961 | 0.000 | * |
| [Session=Early] * [Region=RdHC] * [Frequency=9.4] | 17.773 | 0.000 | * |
| [Session=Early] * [Region=RdHC] * [Frequency=9.6] | 19.363 | 0.000 | * |
| [Session=Early] * [Region=RdHC] * [Frequency=9.8] | 20.807 | 0.000 | * |
| [Session=Early] * [Region=RdHC] * [Frequency=10.0] | 22.152 | 0.000 | * |
| [Session=Early] * [Region=RdHC] * [Frequency=10.2] | 23.366 | 0.000 | * |
| [Session=Early] * [Region=RdHC] * [Frequency=10.4] | 24.419 | 0.000 | * |
| [Session=Early] * [Region=RdHC] * [Frequency=10.6] | 25.298 | 0.000 | * |
| [Session=Early] * [Region=RdHC] * [Frequency=10.8] | 26.447 | 0.000 | * |
| [Session=Early] * [Region=RdHC] * [Frequency=11.0] | 27.534 | 0.000 | * |
| [Session=Early] * [Region=RdHC] * [Frequency=11.2] | 28.455 | 0.000 | * |
| [Session=Early] * [Region=RdHC] * [Frequency=11.4] | 29.132 | 0.000 | * |
| [Session=Early] * [Region=RdHC] * [Frequency=11.6] | 29.741 | 0.000 | * |
| [Session=Early] * [Region=RdHC] * [Frequency=11.8] | 30.294 | 0.000 | * |
| [Session=Early] * [Region=RdHC] * [Frequency=12.0] | 30.718 | 0.000 | * |
| [Session=Early] * [Region=RPFC] * [Frequency=5.0] | 17.765 | 0.000 | * |
| [Session=Early] * [Region=RPFC] * [Frequency=5.2] | 17.833 | 0.000 | * |
| [Session=Early] * [Region=RPFC] * [Frequency=5.4] | 17.998 | 0.000 | * |
| [Session=Early] * [Region=RPFC] * [Frequency=5.6] | 18.213 | 0.000 | * |
| [Session=Early] * [Region=RPFC] * [Frequency=5.8] | 18.587 | 0.000 | * |
| [Session=Early] * [Region=RPFC] * [Frequency=6.0] | 19.052 | 0.000 | * |
| [Session=Early] * [Region=RPFC] * [Frequency=6.2] | 18.858 | 0.000 | * |
| [Session=Early] * [Region=RPFC] * [Frequency=6.4] | 19.077 | 0.000 | * |
| [Session=Early] * [Region=RPFC] * [Frequency=6.6] | 19.416 | 0.000 | * |
| [Session=Early] * [Region=RPFC] * [Frequency=6.8] | 19.806 | 0.000 | * |
| [Session=Early] * [Region=RPFC] * [Frequency=7.0] | 20.586 | 0.000 | * |
| [Session=Early] * [Region=RPFC] * [Frequency=7.2] | 20.743 | 0.000 | * |
| [Session=Early] * [Region=RPFC] * [Frequency=7.4] | 21.589 | 0.000 | * |
| [Session=Early] * [Region=RPFC] * [Frequency=7.6] | 23.055 | 0.000 | * |
| [Session=Early] * [Region=RPFC] * [Frequency=7.8] | 24.687 | 0.000 | * |
| [Session=Early] * [Region=RPFC] * [Frequency=8.0] | 26.939 | 0.000 | * |
| [Session=Early] * [Region=RPFC] * [Frequency=8.2] | 28.491 | 0.000 | * |
| [Session=Early] * [Region=RPFC] * [Frequency=8.4] | 30.702 | 0.000 | * |
| [Session=Early] * [Region=RPFC] * [Frequency=8.6] | 33.439 | 0.000 | * |
| [Session=Early] * [Region=RPFC] * [Frequency=8.8] | 36.082 | 0.000 | * |
| [Session=Early] * [Region=RPFC] * [Frequency=9.0] | 38.904 | 0.000 | * |
| [Session=Early] * [Region=RPFC] * [Frequency=9.2] | 40.422 | 0.000 | * |
| [Session=Early] * [Region=RPFC] * [Frequency=9.4] | 41.883 | 0.000 | * |
| [Session=Early] * [Region=RPFC] * [Frequency=9.6] | 43.483 | 0.000 | * |
| [Session=Early] * [Region=RPFC] * [Frequency=9.8] | 45.032 | 0.000 | * |
| [Session=Early] * [Region=RPFC] * [Frequency=10.0] | 47.101 | 0.000 | * |
| [Session=Early] * [Region=RPFC] * [Frequency=10.2] | 47.873 | 0.000 | * |
| [Session=Early] * [Region=RPFC] * [Frequency=10.4] | 48.817 | 0.000 | * |
| [Session=Early] * [Region=RPFC] * [Frequency=10.6] | 49.924 | 0.000 | * |
| [Session=Early] * [Region=RPFC] * [Frequency=10.8] | 51.124 | 0.000 | * |
| [Session=Early] * [Region=RPFC] * [Frequency=11.0] | 52.945 | 0.000 | * |
| [Session=Early] * [Region=RPFC] * [Frequency=11.2] | 53.281 | 0.000 | * |
| [Session=Early] * [Region=RPFC] * [Frequency=11.4] | 53.661 | 0.000 | * |
| [Session=Early] * [Region=RPFC] * [Frequency=11.6] | 54.239 | 0.000 | * |
| [Session=Early] * [Region=RPFC] * [Frequency=11.8] | 54.882 | 0.000 | * |
| [Session=Early] * [Region=RPFC] * [Frequency=12.0] | 55.971 | 0.000 | * |
| [Session=Early] * [Region=RvHC] * [Frequency=5.0] | 2.891 | 0.089 |  |
| [Session=Early] * [Region=RvHC] * [Frequency=5.2] | 2.511 | 0.113 |  |
| [Session=Early] * [Region=RvHC] * [Frequency=5.4] | 2.160 | 0.142 |  |
| [Session=Early] * [Region=RvHC] * [Frequency=5.6] | 1.776 | 0.183 |  |
| [Session=Early] * [Region=RvHC] * [Frequency=5.8] | 1.359 | 0.244 |  |
| [Session=Early] * [Region=RvHC] * [Frequency=6.0] | 0.955 | 0.328 |  |
| [Session=Early] * [Region=RvHC] * [Frequency=6.2] | 0.615 | 0.433 |  |
| [Session=Early] * [Region=RvHC] * [Frequency=6.4] | 0.414 | 0.520 |  |
| [Session=Early] * [Region=RvHC] * [Frequency=6.6] | 0.288 | 0.592 |  |
| [Session=Early] * [Region=RvHC] * [Frequency=6.8] | 0.241 | 0.624 |  |
| [Session=Early] * [Region=RvHC] * [Frequency=7.0] | 0.246 | 0.620 |  |
| [Session=Early] * [Region=RvHC] * [Frequency=7.2] | 0.279 | 0.598 |  |
| [Session=Early] * [Region=RvHC] * [Frequency=7.4] | 0.396 | 0.529 |  |
| [Session=Early] * [Region=RvHC] * [Frequency=7.6] | 0.639 | 0.424 |  |
| [Session=Early] * [Region=RvHC] * [Frequency=7.8] | 1.059 | 0.303 |  |
| [Session=Early] * [Region=RvHC] * [Frequency=8.0] | 1.731 | 0.188 |  |
| [Session=Early] * [Region=RvHC] * [Frequency=8.2] | 2.652 | 0.103 |  |
| [Session=Early] * [Region=RvHC] * [Frequency=8.4] | 3.855 | 0.050 |  |
| [Session=Early] * [Region=RvHC] * [Frequency=8.6] | 5.276 | 0.022 | * |
| [Session=Early] * [Region=RvHC] * [Frequency=8.8] | 6.849 | 0.009 | * |
| [Session=Early] * [Region=RvHC] * [Frequency=9.0] | 8.659 | 0.003 | * |
| [Session=Early] * [Region=RvHC] * [Frequency=9.2] | 10.348 | 0.001 | * |
| [Session=Early] * [Region=RvHC] * [Frequency=9.4] | 11.941 | 0.001 | * |
| [Session=Early] * [Region=RvHC] * [Frequency=9.6] | 13.213 | 0.000 | * |
| [Session=Early] * [Region=RvHC] * [Frequency=9.8] | 14.533 | 0.000 | * |
| [Session=Early] * [Region=RvHC] * [Frequency=10.0] | 15.675 | 0.000 | * |
| [Session=Early] * [Region=RvHC] * [Frequency=10.2] | 16.678 | 0.000 | * |
| [Session=Early] * [Region=RvHC] * [Frequency=10.4] | 17.580 | 0.000 | * |
| [Session=Early] * [Region=RvHC] * [Frequency=10.6] | 18.638 | 0.000 | * |
| [Session=Early] * [Region=RvHC] * [Frequency=10.8] | 19.677 | 0.000 | * |
| [Session=Early] * [Region=RvHC] * [Frequency=11.0] | 20.804 | 0.000 | * |
| [Session=Early] * [Region=RvHC] * [Frequency=11.2] | 21.686 | 0.000 | * |
| [Session=Early] * [Region=RvHC] * [Frequency=11.4] | 22.369 | 0.000 | * |
| [Session=Early] * [Region=RvHC] * [Frequency=11.6] | 22.988 | 0.000 | * |
| [Session=Early] * [Region=RvHC] * [Frequency=11.8] | 23.642 | 0.000 | * |
| [Session=Early] * [Region=RvHC] * [Frequency=12.0] | 24.114 | 0.000 | * |
| [Session=Late ] * [Region=LdHC] * [Frequency=5.0] | 6.111 | 0.013 | * |
| [Session=Late ] * [Region=LdHC] * [Frequency=5.2] | 5.388 | 0.020 | * |
| [Session=Late ] * [Region=LdHC] * [Frequency=5.4] | 4.560 | 0.033 | * |
| [Session=Late ] * [Region=LdHC] * [Frequency=5.6] | 3.683 | 0.055 |  |
| [Session=Late ] * [Region=LdHC] * [Frequency=5.8] | 2.778 | 0.096 |  |
| [Session=Late ] * [Region=LdHC] * [Frequency=6.0] | 1.947 | 0.163 |  |
| [Session=Late ] * [Region=LdHC] * [Frequency=6.2] | 1.157 | 0.282 |  |
| [Session=Late ] * [Region=LdHC] * [Frequency=6.4] | 0.603 | 0.438 |  |
| [Session=Late ] * [Region=LdHC] * [Frequency=6.6] | 0.294 | 0.588 |  |
| [Session=Late ] * [Region=LdHC] * [Frequency=6.8] | 0.154 | 0.694 |  |
| [Session=Late ] * [Region=LdHC] * [Frequency=7.0] | 0.118 | 0.731 |  |
| [Session=Late ] * [Region=LdHC] * [Frequency=7.2] | 0.138 | 0.710 |  |
| [Session=Late ] * [Region=LdHC] * [Frequency=7.4] | 0.239 | 0.625 |  |
| [Session=Late ] * [Region=LdHC] * [Frequency=7.6] | 0.490 | 0.484 |  |
| [Session=Late ] * [Region=LdHC] * [Frequency=7.8] | 0.980 | 0.322 |  |
| [Session=Late ] * [Region=LdHC] * [Frequency=8.0] | 1.749 | 0.186 |  |
| [Session=Late ] * [Region=LdHC] * [Frequency=8.2] | 2.868 | 0.090 |  |
| [Session=Late ] * [Region=LdHC] * [Frequency=8.4] | 4.363 | 0.037 | * |
| [Session=Late ] * [Region=LdHC] * [Frequency=8.6] | 6.110 | 0.013 | * |
| [Session=Late ] * [Region=LdHC] * [Frequency=8.8] | 8.052 | 0.005 | * |
| [Session=Late ] * [Region=LdHC] * [Frequency=9.0] | 10.141 | 0.001 | * |
| [Session=Late ] * [Region=LdHC] * [Frequency=9.2] | 12.162 | 0.000 | * |
| [Session=Late ] * [Region=LdHC] * [Frequency=9.4] | 13.905 | 0.000 | * |
| [Session=Late ] * [Region=LdHC] * [Frequency=9.6] | 15.631 | 0.000 | * |
| [Session=Late ] * [Region=LdHC] * [Frequency=9.8] | 17.070 | 0.000 | * |
| [Session=Late ] * [Region=LdHC] * [Frequency=10.0] | 18.659 | 0.000 | * |
| [Session=Late ] * [Region=LdHC] * [Frequency=10.2] | 19.786 | 0.000 | * |
| [Session=Late ] * [Region=LdHC] * [Frequency=10.4] | 21.007 | 0.000 | * |
| [Session=Late ] * [Region=LdHC] * [Frequency=10.6] | 22.296 | 0.000 | * |
| [Session=Late ] * [Region=LdHC] * [Frequency=10.8] | 23.319 | 0.000 | * |
| [Session=Late ] * [Region=LdHC] * [Frequency=11.0] | 24.081 | 0.000 | * |
| [Session=Late ] * [Region=LdHC] * [Frequency=11.2] | 24.622 | 0.000 | * |
| [Session=Late ] * [Region=LdHC] * [Frequency=11.4] | 25.339 | 0.000 | * |
| [Session=Late ] * [Region=LdHC] * [Frequency=11.6] | 25.878 | 0.000 | * |
| [Session=Late ] * [Region=LdHC] * [Frequency=11.8] | 26.339 | 0.000 | * |
| [Session=Late ] * [Region=LdHC] * [Frequency=12.0] | 26.773 | 0.000 | * |
| [Session=Late ] * [Region=LPFC] * [Frequency=5.0] | 29.429 | 0.000 | * |
| [Session=Late ] * [Region=LPFC] * [Frequency=5.2] | 29.638 | 0.000 | * |
| [Session=Late ] * [Region=LPFC] * [Frequency=5.4] | 30.160 | 0.000 | * |
| [Session=Late ] * [Region=LPFC] * [Frequency=5.6] | 30.632 | 0.000 | * |
| [Session=Late ] * [Region=LPFC] * [Frequency=5.8] | 30.781 | 0.000 | * |
| [Session=Late ] * [Region=LPFC] * [Frequency=6.0] | 31.322 | 0.000 | * |
| [Session=Late ] * [Region=LPFC] * [Frequency=6.2] | 30.265 | 0.000 | * |
| [Session=Late ] * [Region=LPFC] * [Frequency=6.4] | 29.396 | 0.000 | * |
| [Session=Late ] * [Region=LPFC] * [Frequency=6.6] | 28.978 | 0.000 | * |
| [Session=Late ] * [Region=LPFC] * [Frequency=6.8] | 28.718 | 0.000 | * |
| [Session=Late ] * [Region=LPFC] * [Frequency=7.0] | 29.295 | 0.000 | * |
| [Session=Late ] * [Region=LPFC] * [Frequency=7.2] | 29.715 | 0.000 | * |
| [Session=Late ] * [Region=LPFC] * [Frequency=7.4] | 31.031 | 0.000 | * |
| [Session=Late ] * [Region=LPFC] * [Frequency=7.6] | 33.069 | 0.000 | * |
| [Session=Late ] * [Region=LPFC] * [Frequency=7.8] | 35.901 | 0.000 | * |
| [Session=Late ] * [Region=LPFC] * [Frequency=8.0] | 39.819 | 0.000 | * |
| [Session=Late ] * [Region=LPFC] * [Frequency=8.2] | 42.910 | 0.000 | * |
| [Session=Late ] * [Region=LPFC] * [Frequency=8.4] | 46.174 | 0.000 | * |
| [Session=Late ] * [Region=LPFC] * [Frequency=8.6] | 49.663 | 0.000 | * |
| [Session=Late ] * [Region=LPFC] * [Frequency=8.8] | 53.107 | 0.000 | * |
| [Session=Late ] * [Region=LPFC] * [Frequency=9.0] | 56.801 | 0.000 | * |
| [Session=Late ] * [Region=LPFC] * [Frequency=9.2] | 58.927 | 0.000 | * |
| [Session=Late ] * [Region=LPFC] * [Frequency=9.4] | 61.492 | 0.000 | * |
| [Session=Late ] * [Region=LPFC] * [Frequency=9.6] | 64.153 | 0.000 | * |
| [Session=Late ] * [Region=LPFC] * [Frequency=9.8] | 66.188 | 0.000 | * |
| [Session=Late ] * [Region=LPFC] * [Frequency=10.0] | 68.676 | 0.000 | * |
| [Session=Late ] * [Region=LPFC] * [Frequency=10.2] | 69.530 | 0.000 | * |
| [Session=Late ] * [Region=LPFC] * [Frequency=10.4] | 70.861 | 0.000 | * |
| [Session=Late ] * [Region=LPFC] * [Frequency=10.6] | 71.850 | 0.000 | * |
| [Session=Late ] * [Region=LPFC] * [Frequency=10.8] | 73.042 | 0.000 | * |
| [Session=Late ] * [Region=LPFC] * [Frequency=11.0] | 75.113 | 0.000 | * |
| [Session=Late ] * [Region=LPFC] * [Frequency=11.2] | 75.937 | 0.000 | * |
| [Session=Late ] * [Region=LPFC] * [Frequency=11.4] | 77.132 | 0.000 | * |
| [Session=Late ] * [Region=LPFC] * [Frequency=11.6] | 77.658 | 0.000 | * |
| [Session=Late ] * [Region=LPFC] * [Frequency=11.8] | 78.263 | 0.000 | * |
| [Session=Late ] * [Region=LPFC] * [Frequency=12.0] | 79.842 | 0.000 | * |
| [Session=Late ] * [Region=LvHC] * [Frequency=5.0] | 4.798 | 0.028 | * |
| [Session=Late ] * [Region=LvHC] * [Frequency=5.2] | 4.253 | 0.039 | * |
| [Session=Late ] * [Region=LvHC] * [Frequency=5.4] | 3.643 | 0.056 |  |
| [Session=Late ] * [Region=LvHC] * [Frequency=5.6] | 2.976 | 0.085 |  |
| [Session=Late ] * [Region=LvHC] * [Frequency=5.8] | 2.296 | 0.130 |  |
| [Session=Late ] * [Region=LvHC] * [Frequency=6.0] | 1.651 | 0.199 |  |
| [Session=Late ] * [Region=LvHC] * [Frequency=6.2] | 1.077 | 0.299 |  |
| [Session=Late ] * [Region=LvHC] * [Frequency=6.4] | 0.652 | 0.419 |  |
| [Session=Late ] * [Region=LvHC] * [Frequency=6.6] | 0.378 | 0.539 |  |
| [Session=Late ] * [Region=LvHC] * [Frequency=6.8] | 0.227 | 0.633 |  |
| [Session=Late ] * [Region=LvHC] * [Frequency=7.0] | 0.180 | 0.671 |  |
| [Session=Late ] * [Region=LvHC] * [Frequency=7.2] | 0.206 | 0.650 |  |
| [Session=Late ] * [Region=LvHC] * [Frequency=7.4] | 0.333 | 0.564 |  |
| [Session=Late ] * [Region=LvHC] * [Frequency=7.6] | 0.615 | 0.433 |  |
| [Session=Late ] * [Region=LvHC] * [Frequency=7.8] | 1.108 | 0.293 |  |
| [Session=Late ] * [Region=LvHC] * [Frequency=8.0] | 1.877 | 0.171 |  |
| [Session=Late ] * [Region=LvHC] * [Frequency=8.2] | 2.949 | 0.086 |  |
| [Session=Late ] * [Region=LvHC] * [Frequency=8.4] | 4.366 | 0.037 | * |
| [Session=Late ] * [Region=LvHC] * [Frequency=8.6] | 6.009 | 0.014 | * |
| [Session=Late ] * [Region=LvHC] * [Frequency=8.8] | 7.841 | 0.005 | * |
| [Session=Late ] * [Region=LvHC] * [Frequency=9.0] | 9.808 | 0.002 | * |
| [Session=Late ] * [Region=LvHC] * [Frequency=9.2] | 11.510 | 0.001 | * |
| [Session=Late ] * [Region=LvHC] * [Frequency=9.4] | 13.119 | 0.000 | * |
| [Session=Late ] * [Region=LvHC] * [Frequency=9.6] | 14.611 | 0.000 | * |
| [Session=Late ] * [Region=LvHC] * [Frequency=9.8] | 15.929 | 0.000 | * |
| [Session=Late ] * [Region=LvHC] * [Frequency=10.0] | 17.019 | 0.000 | * |
| [Session=Late ] * [Region=LvHC] * [Frequency=10.2] | 17.952 | 0.000 | * |
| [Session=Late ] * [Region=LvHC] * [Frequency=10.4] | 19.037 | 0.000 | * |
| [Session=Late ] * [Region=LvHC] * [Frequency=10.6] | 20.154 | 0.000 | * |
| [Session=Late ] * [Region=LvHC] * [Frequency=10.8] | 21.293 | 0.000 | * |
| [Session=Late ] * [Region=LvHC] * [Frequency=11.0] | 22.406 | 0.000 | * |
| [Session=Late ] * [Region=LvHC] * [Frequency=11.2] | 23.227 | 0.000 | * |
| [Session=Late ] * [Region=LvHC] * [Frequency=11.4] | 23.956 | 0.000 | * |
| [Session=Late ] * [Region=LvHC] * [Frequency=11.6] | 24.788 | 0.000 | * |
| [Session=Late ] * [Region=LvHC] * [Frequency=11.8] | 25.345 | 0.000 | * |
| [Session=Late ] * [Region=LvHC] * [Frequency=12.0] | 25.898 | 0.000 | * |
| [Session=Late ] * [Region=RdHC] * [Frequency=5.0] | 8.930 | 0.003 | * |
| [Session=Late ] * [Region=RdHC] * [Frequency=5.2] | 8.128 | 0.004 | * |
| [Session=Late ] * [Region=RdHC] * [Frequency=5.4] | 7.275 | 0.007 | * |
| [Session=Late ] * [Region=RdHC] * [Frequency=5.6] | 6.176 | 0.013 | * |
| [Session=Late ] * [Region=RdHC] * [Frequency=5.8] | 5.009 | 0.025 | * |
| [Session=Late ] * [Region=RdHC] * [Frequency=6.0] | 3.808 | 0.051 |  |
| [Session=Late ] * [Region=RdHC] * [Frequency=6.2] | 2.634 | 0.105 |  |
| [Session=Late ] * [Region=RdHC] * [Frequency=6.4] | 1.701 | 0.192 |  |
| [Session=Late ] * [Region=RdHC] * [Frequency=6.6] | 1.122 | 0.289 |  |
| [Session=Late ] * [Region=RdHC] * [Frequency=6.8] | 0.784 | 0.376 |  |
| [Session=Late ] * [Region=RdHC] * [Frequency=7.0] | 0.659 | 0.417 |  |
| [Session=Late ] * [Region=RdHC] * [Frequency=7.2] | 0.693 | 0.405 |  |
| [Session=Late ] * [Region=RdHC] * [Frequency=7.4] | 0.904 | 0.342 |  |
| [Session=Late ] * [Region=RdHC] * [Frequency=7.6] | 1.323 | 0.250 |  |
| [Session=Late ] * [Region=RdHC] * [Frequency=7.8] | 2.024 | 0.155 |  |
| [Session=Late ] * [Region=RdHC] * [Frequency=8.0] | 3.095 | 0.079 |  |
| [Session=Late ] * [Region=RdHC] * [Frequency=8.2] | 4.520 | 0.034 | * |
| [Session=Late ] * [Region=RdHC] * [Frequency=8.4] | 6.304 | 0.012 | * |
| [Session=Late ] * [Region=RdHC] * [Frequency=8.6] | 8.403 | 0.004 | * |
| [Session=Late ] * [Region=RdHC] * [Frequency=8.8] | 10.646 | 0.001 | * |
| [Session=Late ] * [Region=RdHC] * [Frequency=9.0] | 12.946 | 0.000 | * |
| [Session=Late ] * [Region=RdHC] * [Frequency=9.2] | 15.071 | 0.000 | * |
| [Session=Late ] * [Region=RdHC] * [Frequency=9.4] | 17.191 | 0.000 | * |
| [Session=Late ] * [Region=RdHC] * [Frequency=9.6] | 19.202 | 0.000 | * |
| [Session=Late ] * [Region=RdHC] * [Frequency=9.8] | 21.065 | 0.000 | * |
| [Session=Late ] * [Region=RdHC] * [Frequency=10.0] | 22.852 | 0.000 | * |
| [Session=Late ] * [Region=RdHC] * [Frequency=10.2] | 24.350 | 0.000 | * |
| [Session=Late ] * [Region=RdHC] * [Frequency=10.4] | 25.405 | 0.000 | * |
| [Session=Late ] * [Region=RdHC] * [Frequency=10.6] | 26.458 | 0.000 | * |
| [Session=Late ] * [Region=RdHC] * [Frequency=10.8] | 27.556 | 0.000 | * |
| [Session=Late ] * [Region=RdHC] * [Frequency=11.0] | 28.459 | 0.000 | * |
| [Session=Late ] * [Region=RdHC] * [Frequency=11.2] | 28.920 | 0.000 | * |
| [Session=Late ] * [Region=RdHC] * [Frequency=11.4] | 29.454 | 0.000 | * |
| [Session=Late ] * [Region=RdHC] * [Frequency=11.6] | 29.774 | 0.000 | * |
| [Session=Late ] * [Region=RdHC] * [Frequency=11.8] | 30.376 | 0.000 | * |
| [Session=Late ] * [Region=RdHC] * [Frequency=12.0] | 30.903 | 0.000 | * |
| [Session=Late ] * [Region=RPFC] * [Frequency=5.0] | 14.972 | 0.000 | * |
| [Session=Late ] * [Region=RPFC] * [Frequency=5.2] | 15.549 | 0.000 | * |
| [Session=Late ] * [Region=RPFC] * [Frequency=5.4] | 16.089 | 0.000 | * |
| [Session=Late ] * [Region=RPFC] * [Frequency=5.6] | 16.529 | 0.000 | * |
| [Session=Late ] * [Region=RPFC] * [Frequency=5.8] | 16.586 | 0.000 | * |
| [Session=Late ] * [Region=RPFC] * [Frequency=6.0] | 16.590 | 0.000 | * |
| [Session=Late ] * [Region=RPFC] * [Frequency=6.2] | 16.335 | 0.000 | * |
| [Session=Late ] * [Region=RPFC] * [Frequency=6.4] | 15.960 | 0.000 | * |
| [Session=Late ] * [Region=RPFC] * [Frequency=6.6] | 15.697 | 0.000 | * |
| [Session=Late ] * [Region=RPFC] * [Frequency=6.8] | 15.566 | 0.000 | * |
| [Session=Late ] * [Region=RPFC] * [Frequency=7.0] | 15.702 | 0.000 | * |
| [Session=Late ] * [Region=RPFC] * [Frequency=7.2] | 16.090 | 0.000 | * |
| [Session=Late ] * [Region=RPFC] * [Frequency=7.4] | 17.053 | 0.000 | * |
| [Session=Late ] * [Region=RPFC] * [Frequency=7.6] | 18.544 | 0.000 | * |
| [Session=Late ] * [Region=RPFC] * [Frequency=7.8] | 20.365 | 0.000 | * |
| [Session=Late ] * [Region=RPFC] * [Frequency=8.0] | 22.464 | 0.000 | * |
| [Session=Late ] * [Region=RPFC] * [Frequency=8.2] | 24.828 | 0.000 | * |
| [Session=Late ] * [Region=RPFC] * [Frequency=8.4] | 27.523 | 0.000 | * |
| [Session=Late ] * [Region=RPFC] * [Frequency=8.6] | 30.107 | 0.000 | * |
| [Session=Late ] * [Region=RPFC] * [Frequency=8.8] | 32.468 | 0.000 | * |
| [Session=Late ] * [Region=RPFC] * [Frequency=9.0] | 34.827 | 0.000 | * |
| [Session=Late ] * [Region=RPFC] * [Frequency=9.2] | 36.756 | 0.000 | * |
| [Session=Late ] * [Region=RPFC] * [Frequency=9.4] | 38.499 | 0.000 | * |
| [Session=Late ] * [Region=RPFC] * [Frequency=9.6] | 40.136 | 0.000 | * |
| [Session=Late ] * [Region=RPFC] * [Frequency=9.8] | 41.573 | 0.000 | * |
| [Session=Late ] * [Region=RPFC] * [Frequency=10.0] | 43.077 | 0.000 | * |
| [Session=Late ] * [Region=RPFC] * [Frequency=10.2] | 44.217 | 0.000 | * |
| [Session=Late ] * [Region=RPFC] * [Frequency=10.4] | 45.324 | 0.000 | * |
| [Session=Late ] * [Region=RPFC] * [Frequency=10.6] | 46.432 | 0.000 | * |
| [Session=Late ] * [Region=RPFC] * [Frequency=10.8] | 47.207 | 0.000 | * |
| [Session=Late ] * [Region=RPFC] * [Frequency=11.0] | 48.312 | 0.000 | * |
| [Session=Late ] * [Region=RPFC] * [Frequency=11.2] | 49.641 | 0.000 | * |
| [Session=Late ] * [Region=RPFC] * [Frequency=11.4] | 50.591 | 0.000 | * |
| [Session=Late ] * [Region=RPFC] * [Frequency=11.6] | 50.951 | 0.000 | * |
| [Session=Late ] * [Region=RPFC] * [Frequency=11.8] | 51.290 | 0.000 | * |
| [Session=Late ] * [Region=RPFC] * [Frequency=12.0] | 51.416 | 0.000 | * |
| [Session=Late ] * [Region=RvHC] * [Frequency=5.0] | 2.077 | 0.150 |  |
| [Session=Late ] * [Region=RvHC] * [Frequency=5.2] | 1.760 | 0.185 |  |
| [Session=Late ] * [Region=RvHC] * [Frequency=5.4] | 1.404 | 0.236 |  |
| [Session=Late ] * [Region=RvHC] * [Frequency=5.6] | 1.034 | 0.309 |  |
| [Session=Late ] * [Region=RvHC] * [Frequency=5.8] | 0.683 | 0.408 |  |
| [Session=Late ] * [Region=RvHC] * [Frequency=6.0] | 0.362 | 0.547 |  |
| [Session=Late ] * [Region=RvHC] * [Frequency=6.2] | 0.138 | 0.711 |  |
| [Session=Late ] * [Region=RvHC] * [Frequency=6.4] | 0.020 | 0.887 |  |
| [Session=Late ] * [Region=RvHC] * [Frequency=6.6] | 0.001 | 0.971 |  |
| [Session=Late ] * [Region=RvHC] * [Frequency=6.8] | 0.027 | 0.869 |  |
| [Session=Late ] * [Region=RvHC] * [Frequency=7.0] | 0.034 | 0.853 |  |
| [Session=Late ] * [Region=RvHC] * [Frequency=7.2] | 0.018 | 0.895 |  |
| [Session=Late ] * [Region=RvHC] * [Frequency=7.6] | 0.041 | 0.840 |  |
| [Session=Late ] * [Region=RvHC] * [Frequency=7.8] | 0.230 | 0.631 |  |
| [Session=Late ] * [Region=RvHC] * [Frequency=8.0] | 0.685 | 0.408 |  |
| [Session=Late ] * [Region=RvHC] * [Frequency=8.2] | 1.412 | 0.235 |  |
| [Session=Late ] * [Region=RvHC] * [Frequency=8.4] | 2.442 | 0.118 |  |
| [Session=Late ] * [Region=RvHC] * [Frequency=8.6] | 3.744 | 0.053 |  |
| [Session=Late ] * [Region=RvHC] * [Frequency=8.8] | 5.269 | 0.022 | * |
| [Session=Late ] * [Region=RvHC] * [Frequency=9.0] | 6.847 | 0.009 | * |
| [Session=Late ] * [Region=RvHC] * [Frequency=9.2] | 8.316 | 0.004 | * |
| [Session=Late ] * [Region=RvHC] * [Frequency=9.4] | 9.712 | 0.002 | * |
| [Session=Late ] * [Region=RvHC] * [Frequency=9.6] | 11.017 | 0.001 | * |
| [Session=Late ] * [Region=RvHC] * [Frequency=9.8] | 12.152 | 0.000 | * |
| [Session=Late ] * [Region=RvHC] * [Frequency=10.0] | 13.264 | 0.000 | * |
| [Session=Late ] * [Region=RvHC] * [Frequency=10.2] | 14.187 | 0.000 | * |
| [Session=Late ] * [Region=RvHC] * [Frequency=10.4] | 15.040 | 0.000 | * |
| [Session=Late ] * [Region=RvHC] * [Frequency=10.6] | 15.923 | 0.000 | * |
| [Session=Late ] * [Region=RvHC] * [Frequency=10.8] | 16.825 | 0.000 | * |
| [Session=Late ] * [Region=RvHC] * [Frequency=11.0] | 17.668 | 0.000 | * |
| [Session=Late ] * [Region=RvHC] * [Frequency=11.2] | 18.329 | 0.000 | * |
| [Session=Late ] * [Region=RvHC] * [Frequency=11.4] | 18.935 | 0.000 | * |
| [Session=Late ] * [Region=RvHC] * [Frequency=11.6] | 19.623 | 0.000 | * |
| [Session=Late ] * [Region=RvHC] * [Frequency=11.8] | 20.127 | 0.000 | * |
| [Session=Late ] * [Region=RvHC] * [Frequency=12.0] | 20.730 | 0.000 | * |
| [Session=Late] * [Region=RvHC] * [Frequency=7.4] | 0a |  |  |

Extended Table 2-3. Results of GEE Session × Region × Frequency interactions for signal amplitude at peak 7.4 Hz signal only.

Session × Region × Frequency Wald Value Sig.

| [Session=Early] * [Region=LdHC] * [Frequency=7.4] | 1.744 | 0.187 |  |
| --- | --- | --- | --- |
| [Session=Early] * [Region=LPFC] * [Frequency=7.4] | 36.573 | 0.000 | * |
| [Session=Early] * [Region=LvHC] * [Frequency=7.4] | 0.943 | 0.331 |  |
| [Session=Early] * [Region=RdHC] * [Frequency=7.4] | 2.382 | 0.123 |  |
| [Session=Early] * [Region=RPFC] * [Frequency=7.4] | 21.589 | 0.000 | * |
| [Session=Early] * [Region=RvHC] * [Frequency=7.4] | 0.396 | 0.529 |  |
| [Session=Late] * [Region=LdHC] * [Frequency=7.4] | 0.239 | 0.625 |  |
| [Session=Late] * [Region=LPFC] * [Frequency=7.4] | 31.031 | 0.000 | * |
| [Session=Late] * [Region=LvHC] * [Frequency=7.4] | 0.333 | 0.564 |  |
| [Session=Late] * [Region=RdHC] * [Frequency=7.4] | 0.904 | 0.342 |  |
| [Session=Late] * [Region=RPFC] * [Frequency=7.4] | 17.053 | 0.000 | * |
| [Session=Late] * [Region=RvHC] * [Frequency=7.4] | 0a |  |  |

Extended Table 3-1. Results of GEE Session × Comparison interactions for whole session theta coherence among dorsal hippocampus-medial prefrontal cortex (dHC-mPFC) and ventral hippocampus-medial prefrontal cortex (vHC-mPFC) regional comparisons in Early and Late training sessions. Ventral hippocampus-medial prefrontal cortex regional comparison in the Early training session as reference (0a).

Session × Comparison Wald value Sig.

[Session=Late] * [Comparison=dHC-mPFC] 50.767 0.000 *

[Session=Late] * [Comparison=vHC-mPFC] 28.858 0.000 *

[Session=Early] * [Comparison=dHC-mPFC] 12.031 0.001 *

[Session=Early] * [Comparison=vHC-mPFC] 0a

Extended Table 3-2. Results of GEE Session × Comparison × Frequency interactions for whole session theta coherence among dorsal hippocampus-medial prefrontal cortex (dHC-mPFC) and ventral hippocampus-medial prefrontal cortex (vHC-mPFC) regional comparisons in Early and Late training sessions. Ventral hippocampus-medial prefrontal cortex regional comparison at 7.40Hz in the Late training session as reference (0a).

Session × Comparison × Frequency Wald value Sig.

[Session=Early] * [Comparison=vHC-mPFC] * [Frequency=5.42] 23.853 0.000 *

[Session=Early] * [Comparison=vHC-mPFC] * [Frequency=5.92] 18.609 0.000 *

[Session=Early] * [Comparison=vHC-mPFC] * [Frequency=6.41] 10.394 0.001 *

[Session=Early] * [Comparison=vHC-mPFC] * [Frequency=6.90] 5.692 0.017 *

[Session=Early] * [Comparison=vHC-mPFC] * [Frequency=7.40] 4.198 0.040 *

[Session=Early] * [Comparison=vHC-mPFC] * [Frequency=7.89] 5.985 0.014 *

[Session=Early] * [Comparison=vHC-mPFC] * [Frequency=8.38] 14.555 0.000 *

[Session=Early] * [Comparison=vHC-mPFC] * [Frequency=8.88] 35.006 0.000 *

[Session=Early] * [Comparison=vHC-mPFC] * [Frequency=9.37] 53.891 0.000 *

[Session=Early] * [Comparison=vHC-mPFC] * [Frequency=9.86] 58.354 0.000 *

[Session=Early] * [Comparison=vHC-mPFC] * [Frequency=10.36] 56.718 0.000 *

[Session=Early] * [Comparison=vHC-mPFC] * [Frequency=10.85] 59.530 0.000 *

[Session=Early] * [Comparison=vHC-mPFC] * [Frequency=11.34] 68.426 0.000 *

[Session=Early] * [Comparison=vHC-mPFC] * [Frequency=11.84] 74.605 0.000 *

[Session=Early] * [Comparison=dHC-mPFC] * [Frequency=5.42] 13.263 0.000 *

[Session=Early] * [Comparison=dHC-mPFC] * [Frequency=5.92] 11.092 0.001 *

[Session=Early] * [Comparison=dHC-mPFC] * [Frequency=6.41] 6.992 0.008 *

[Session=Early] * [Comparison=dHC-mPFC] * [Frequency=6.90] 3.929 0.047 *

[Session=Early] * [Comparison=dHC-mPFC] * [Frequency=7.40] 2.715 0.099

[Session=Early] * [Comparison=dHC-mPFC] * [Frequency=7.89] 4.007 0.045 *

[Session=Early] * [Comparison=dHC-mPFC] * [Frequency=8.38] 9.618 0.002 *

[Session=Early] * [Comparison=dHC-mPFC] * [Frequency=8.88] 18.387 0.000 *

[Session=Early] * [Comparison=dHC-mPFC] * [Frequency=9.37] 22.353 0.000 *

[Session=Early] * [Comparison=dHC-mPFC] * [Frequency=9.86] 24.636 0.000 *

[Session=Early] * [Comparison=dHC-mPFC] * [Frequency=10.36] 28.802 0.000 *

[Session=Early] * [Comparison=dHC-mPFC] * [Frequency=10.85] 33.815 0.000 *

[Session=Early] * [Comparison=dHC-mPFC] * [Frequency=11.34] 37.884 0.000 *

[Session=Early] * [Comparison=dHC-mPFC] * [Frequency=11.84] 39.473 0.000 *

[Session=Late] * [Comparison=vHC-mPFC] * [Frequency=5.42] 22.912 0.000 *

[Session=Late] * [Comparison=vHC-mPFC] * [Frequency=5.92] 10.043 0.002 *

[Session=Late] * [Comparison=vHC-mPFC] * [Frequency=6.41] 2.326 0.127

[Session=Late] * [Comparison=vHC-mPFC] * [Frequency=6.90] 0.160 0.689

[Session=Late] * [Comparison=vHC-mPFC] * [Frequency=7.89] 0.149 0.700

[Session=Late] * [Comparison=vHC-mPFC] * [Frequency=8.38] 2.471 0.116

[Session=Late] * [Comparison=vHC-mPFC] * [Frequency=8.88] 11.313 0.001 *

[Session=Late] * [Comparison=vHC-mPFC] * [Frequency=9.37] 25.068 0.000 *

[Session=Late] * [Comparison=vHC-mPFC] * [Frequency=9.86] 35.382 0.000 *

[Session=Late] * [Comparison=vHC-mPFC] * [Frequency=10.36] 43.328 0.000 *

[Session=Late] * [Comparison=vHC-mPFC] * [Frequency=10.85] 49.674 0.000 *

[Session=Late] * [Comparison=vHC-mPFC] * [Frequency=11.34] 52.851 0.000 *

[Session=Late] * [Comparison=vHC-mPFC] * [Frequency=11.84] 53.467 0.000 *

[Session=Late] * [Comparison=dHC-mPFC] * [Frequency=5.42] 18.263 0.000 *

[Session=Late] * [Comparison=dHC-mPFC] * [Frequency=5.92] 9.685 0.002 *

[Session=Late] * [Comparison=dHC-mPFC] * [Frequency=6.41] 2.595 0.107

[Session=Late] * [Comparison=dHC-mPFC] * [Frequency=6.90] 0.228 0.633

[Session=Late] * [Comparison=dHC-mPFC] * [Frequency=7.40] 0.002 0.965

[Session=Late] * [Comparison=dHC-mPFC] * [Frequency=7.89] 0.108 0.742

[Session=Late] * [Comparison=dHC-mPFC] * [Frequency=8.38] 1.627 0.202

[Session=Late] * [Comparison=dHC-mPFC] * [Frequency=8.88] 6.763 0.009 *

[Session=Late] * [Comparison=dHC-mPFC] * [Frequency=9.37] 12.943 0.000 *

[Session=Late] * [Comparison=dHC-mPFC] * [Frequency=9.86] 16.226 0.000 *

[Session=Late] * [Comparison=dHC-mPFC] * [Frequency=10.36] 18.748 0.000 *

[Session=Late] * [Comparison=dHC-mPFC] * [Frequency=10.85] 22.336 0.000 *

[Session=Late] * [Comparison=dHC-mPFC] * [Frequency=11.34] 26.333 0.000 *

[Session=Late] * [Comparison=dHC-mPFC] * [Frequency=11.84] 29.017 0.000 *

[Session=Late] * [Comparison=vHC-mPFC] * [Frequency=7.40] 0a

Extended Table 3-3. Results of GEE Session × Synapses interactions for whole session theta coherence among ipsilateral and contralateral dorsal hippocampus-ventral hippocampus (dHC-vHC) regional comparisons in Early and Late training sessions. Ipsilateral dHC-vHC regional comparison in the Early training session as reference (0a).

Session × Synapses Wald Value Sig.

[Session=Late] * [Synapses=Contra] 7.093 0.008*

[Session=Late] * [Synapses=Ipsi] 11.048 0.001*

[Session=Early] * [Synapses=Contra] 2.783 0.095

[Session=Early] * [Synapses=Ipsi] 0a

Extended Table 3-4. Results of GEE Session × Synapses × Frequency interactions for whole session theta coherence among ipsilateral and contralateral dorsal hippocampus-ventral hippocampus (dHC-vHC) regional comparisons in Early and Late training sessions. Contralateral dHC-vHC regional comparison at 7.40Hz in the Early training session as reference (0a).

Session × Synapses × Frequency Wald Value Sig.

[Session=Early] * [Synapses=Ipsi] * [Frequency=5.42] 4.370 0.037 *

[Session=Early] * [Synapses=Ipsi] * [Frequency=5.92] 1.515 0.218

[Session=Early] * [Synapses=Ipsi] * [Frequency=6.41] 0.235 0.628

[Session=Early] * [Synapses=Ipsi] * [Frequency=6.90] 0.003 0.957

[Session=Early] * [Synapses=Ipsi] * [Frequency=7.40] 0.000 1.000

[Session=Early] * [Synapses=Ipsi] * [Frequency=7.89] 0.116 0.734

[Session=Early] * [Synapses=Ipsi] * [Frequency=8.38] 1.212 0.271

[Session=Early] * [Synapses=Ipsi] * [Frequency=8.88] 4.818 0.028 *

[Session=Early] * [Synapses=Ipsi] * [Frequency=9.37] 10.662 0.001 *

[Session=Early] * [Synapses=Ipsi] * [Frequency=9.86] 16.897 0.000 *

[Session=Early] * [Synapses=Ipsi] * [Frequency=10.36] 24.185 0.000 *

[Session=Early] * [Synapses=Ipsi] * [Frequency=10.85] 32.478 0.000 *

[Session=Early] * [Synapses=Ipsi] * [Frequency=11.34] 38.493 0.000 *

[Session=Early] * [Synapses=Ipsi] * [Frequency=11.84] 41.709 0.000 *

[Session=Early] * [Synapses=Contra] * [Frequency=5.42] 6.056 0.014 *

[Session=Early] * [Synapses=Contra] * [Frequency=5.92] 2.277 0.131

[Session=Early] * [Synapses=Contra] * [Frequency=6.41] 0.391 0.532

[Session=Early] * [Synapses=Contra] * [Frequency=6.90] 0.009 0.924

[Session=Early] * [Synapses=Contra] * [Frequency=7.89] 0.123 0.726

[Session=Early] * [Synapses=Contra] * [Frequency=8.38] 1.626 0.202

[Session=Early] * [Synapses=Contra] * [Frequency=8.88] 8.151 0.004 *

[Session=Early] * [Synapses=Contra] * [Frequency=9.37] 21.393 0.000 *

[Session=Early] * [Synapses=Contra] * [Frequency=9.86] 35.254 0.000 *

[Session=Early] * [Synapses=Contra] * [Frequency=10.36] 47.581 0.000 *

[Session=Early] * [Synapses=Contra] * [Frequency=10.85] 59.770 0.000 *

[Session=Early] * [Synapses=Contra] * [Frequency=11.34] 71.231 0.000 *

[Session=Early] * [Synapses=Contra] * [Frequency=11.84] 83.680 0.000 *

[Session=Late] * [Synapses=Ipsi] * [Frequency=5.42] 1.326 0.249

[Session=Late] * [Synapses=Ipsi] * [Frequency=5.91] 0.016 0.901

[Session=Late] * [Synapses=Ipsi] * [Frequency=6.41] 0.340 0.560

[Session=Late] * [Synapses=Ipsi] * [Frequency=6.90] 0.892 0.345

[Session=Late] * [Synapses=Ipsi] * [Frequency=7.40] 1.042 0.307

[Session=Late] * [Synapses=Ipsi] * [Frequency=7.89] 0.651 0.420

[Session=Late] * [Synapses=Ipsi] * [Frequency=8.38] 0.042 0.838

[Session=Late] * [Synapses=Ipsi] * [Frequency=8.88] 0.718 0.397

[Session=Late] * [Synapses=Ipsi] * [Frequency=9.37] 4.597 0.032 *

[Session=Late] * [Synapses=Ipsi] * [Frequency=9.86] 10.831 0.001 *

[Session=Late] * [Synapses=Ipsi] * [Frequency=10.35] 16.938 0.000 *

[Session=Late] * [Synapses=Ipsi] * [Frequency=10.85] 21.651 0.000 *

[Session=Late] * [Synapses=Ipsi] * [Frequency=11.34] 25.019 0.000 *

[Session=Late] * [Synapses=Ipsi] * [Frequency=11.84] 27.839 0.000 *

[Session=Late] * [Synapses=Contra] * [Frequency=5.42] 2.029 0.154

[Session=Late] * [Synapses=Contra] * [Frequency=5.92] 0.107 0.744

[Session=Late] * [Synapses=Contra] * [Frequency=6.41] 0.229 0.632

[Session=Late] * [Synapses=Contra] * [Frequency=6.90] 0.837 0.360

[Session=Late] * [Synapses=Contra] * [Frequency=7.40] 1.053 0.305

[Session=Late] * [Synapses=Contra] * [Frequency=7.89] 0.661 0.416

[Session=Late] * [Synapses=Contra] * [Frequency=8.38] 0.034 0.853

[Session=Late] * [Synapses=Contra] * [Frequency=8.88] 0.873 0.350

[Session=Late] * [Synapses=Contra] * [Frequency=9.37] 5.507 0.019 *

[Session=Late] * [Synapses=Contra] * [Frequency=9.86] 13.347 0.000 *

[Session=Late] * [Synapses=Contra] * [Frequency=10.36] 22.207 0.000 *

[Session=Late] * [Synapses=Contra] * [Frequency=10.85] 30.816 0.000 *

[Session=Late] * [Synapses=Contra] * [Frequency=11.34] 38.830 0.000 *

[Session=Late] * [Synapses=Contra] * [Frequency=11.84] 46.857 0.000 *

[Session=Early] * [Synapses=Contra] * [Frequency=7.40] 0a

Extended Table 3-5. Results of GEE Session × Comparison interactions for whole session theta coherence among homotopic regional comparisons in Early and Late training sessions. Ventral hippocampus homotopic regional comparison in the Late training session as reference (0a).

Session × Comparison Wald value Sig.

[Session=Early] * [Comparison=dHC-dHC] 0.111 0.739

[Session=Early] * [Comparison=mPFC-mPFC] 72.560 0.000 *

[Session=Early] * [Comparison=vHC-vHC] 8.047 0.005 *

[Session=Late] * [Comparison=dHC-dHC] 4.178 0.041 *

[Session=Late] * [Comparison=mPFC-mPFC] 27.797 0.000 *

[Session=Late] * [Comparison=vHC-vHC] 0a

Extended Table 3-6. Results of GEE Session × Comparison × Frequency interactions for whole session theta coherence among homotopic regional comparisons in Early and Late training sessions. Medial prefrontal cortex homotopic regional comparison in the Early training session as reference (0a).

Session × Comparison × Frequency Wald value Sig.

[Session=Early] * [Comparison=dHC-dHC] * [Frequency=5.42] 1.572 0.210

[Session=Early] * [Comparison=dHC-dHC] * [Frequency=5.92] 2.570 0.109

[Session=Early] * [Comparison=dHC-dHC] * [Frequency=6.41] 3.506 0.061

[Session=Early] * [Comparison=dHC-dHC] * [Frequency=6.90] 4.229 0.040 *

[Session=Early] * [Comparison=dHC-dHC] * [Frequency=7.40] 4.434 0.035 *

[Session=Early] * [Comparison=dHC-dHC] * [Frequency=7.89] 3.924 0.048 *

[Session=Early] * [Comparison=dHC-dHC] * [Frequency=8.38] 2.824 0.093

[Session=Early] * [Comparison=dHC-dHC] * [Frequency=8.88] 1.519 0.218

[Session=Early] * [Comparison=dHC-dHC] * [Frequency=9.37] 0.577 0.447

[Session=Early] * [Comparison=dHC-dHC] * [Frequency=9.86] 0.185 0.667

[Session=Early] * [Comparison=dHC-dHC] * [Frequency=10.36] 0.043 0.835

[Session=Early] * [Comparison=dHC-dHC] * [Frequency=10.85] 0.001 0.980

[Session=Early] * [Comparison=dHC-dHC] * [Frequency=11.34] 0.075 0.785

[Session=Early] * [Comparison=dHC-dHC] * [Frequency=11.84] 0.197 0.658

[Session=Early] * [Comparison=mPFC-mPFC] * [Frequency=5.42] 0.248 0.618

[Session=Early] * [Comparison=mPFC-mPFC] * [Frequency=5.92] 0.255 0.614

[Session=Early] * [Comparison=mPFC-mPFC] * [Frequency=6.41] 0.192 0.662

[Session=Early] * [Comparison=mPFC-mPFC] * [Frequency=6.90] 0.038 0.846

[Session=Early] * [Comparison=mPFC-mPFC] * [Frequency=7.89] 0.010 0.919

[Session=Early] * [Comparison=mPFC-mPFC] * [Frequency=8.38] 0.288 0.592

[Session=Early] * [Comparison=mPFC-mPFC] * [Frequency=8.88] 1.194 0.275

[Session=Early] * [Comparison=mPFC-mPFC] * [Frequency=9.37] 2.149 0.143

[Session=Early] * [Comparison=mPFC-mPFC] * [Frequency=9.86] 2.745 0.098

[Session=Early] * [Comparison=mPFC-mPFC] * [Frequency=10.36] 3.496 0.062

[Session=Early] * [Comparison=mPFC-mPFC] * [Frequency=10.85] 4.619 0.032 *

[Session=Early] * [Comparison=mPFC-mPFC] * [Frequency=11.34] 5.672 0.017 *

[Session=Early] * [Comparison=mPFC-mPFC] * [Frequency=11.84] 6.026 0.014 *

[Session=Early] * [Comparison=vHC-vHC] * [Frequency=5.42] 0.692 0.405

[Session=Early] * [Comparison=vHC-vHC] * [Frequency=5.92] 1.543 0.214

[Session=Early] * [Comparison=vHC-vHC] * [Frequency=6.41] 2.501 0.114

[Session=Early] * [Comparison=vHC-vHC] * [Frequency=6.90] 3.149 0.076

[Session=Early] * [Comparison=vHC-vHC] * [Frequency=7.40] 3.299 0.069

[Session=Early] * [Comparison=vHC-vHC] * [Frequency=7.89] 2.767 0.096

[Session=Early] * [Comparison=vHC-vHC] * [Frequency=8.38] 1.430 0.232

[Session=Early] * [Comparison=vHC-vHC] * [Frequency=8.88] 0.156 0.693

[Session=Early] * [Comparison=vHC-vHC] * [Frequency=9.37] 0.195 0.659

[Session=Early] * [Comparison=vHC-vHC] * [Frequency=9.86] 1.026 0.311

[Session=Early] * [Comparison=vHC-vHC] * [Frequency=10.36] 1.846 0.174

[Session=Early] * [Comparison=vHC-vHC] * [Frequency=10.85] 2.412 0.120

[Session=Early] * [Comparison=vHC-vHC] * [Frequency=11.34] 2.971 0.085

[Session=Early] * [Comparison=vHC-vHC] * [Frequency=11.84] 3.944 0.047

[Session=Late] * [Comparison=dHC-dHC] * [Frequency=5.425] 2.880 0.090

[Session=Late] * [Comparison=dHC-dHC] * [Frequency=5.92] 4.168 0.041 *

[Session=Late] * [Comparison=dHC-dHC] * [Frequency=6.41] 5.157 0.023 *

[Session=Late] * [Comparison=dHC-dHC] * [Frequency=6.90] 5.676 0.017 *

[Session=Late] * [Comparison=dHC-dHC] * [Frequency=7.40] 5.786 0.016 *

[Session=Late] * [Comparison=dHC-dHC] * [Frequency=7.89] 5.485 0.019 *

[Session=Late] * [Comparison=dHC-dHC] * [Frequency=8.38] 4.690 0.030 *

[Session=Late] * [Comparison=dHC-dHC] * [Frequency=8.88] 3.545 0.060

[Session=Late] * [Comparison=dHC-dHC] * [Frequency=9.37] 2.516 0.113

[Session=Late] * [Comparison=dHC-dHC] * [Frequency=9.86] 1.883 0.170

[Session=Late] * [Comparison=dHC-dHC] * [Frequency=10.36] 1.503 0.220

[Session=Late] * [Comparison=dHC-dHC] * [Frequency=10.85] 1.208 0.272

[Session=Late] * [Comparison=dHC-dHC] * [Frequency=11.34] 0.948 0.330

[Session=Late] * [Comparison=dHC-dHC] * [Frequency=11.84] 0.792 0.374

[Session=Late] * [Comparison=mPFC-mPFC] * [Frequency=5.42] 0.032 0.858

[Session=Late] * [Comparison=mPFC-mPFC] * [Frequency=5.92] 0.166 0.683

[Session=Late] * [Comparison=mPFC-mPFC] * [Frequency=6.41] 0.387 0.534

[Session=Late] * [Comparison=mPFC-mPFC] * [Frequency=6.90] 0.613 0.434

[Session=Late] * [Comparison=mPFC-mPFC] * [Frequency=7.40] 0.678 0.410

[Session=Late] * [Comparison=mPFC-mPFC] * [Frequency=7.89] 0.447 0.504

[Session=Late] * [Comparison=mPFC-mPFC] * [Frequency=8.38] 0.079 0.778

[Session=Late] * [Comparison=mPFC-mPFC] * [Frequency=8.88] 0.051 0.821

[Session=Late] * [Comparison=mPFC-mPFC] * [Frequency=9.37] 0.363 0.547

[Session=Late] * [Comparison=mPFC-mPFC] * [Frequency=9.86] 0.668 0.414

[Session=Late] * [Comparison=mPFC-mPFC] * [Frequency=10.36] 1.123 0.289

[Session=Late] * [Comparison=mPFC-mPFC] * [Frequency=10.85] 1.844 0.174

[Session=Late] * [Comparison=mPFC-mPFC] * [Frequency=11.34] 2.608 0.106

[Session=Late] * [Comparison=mPFC-mPFC] * [Frequency=11.84] 3.082 0.079

[Session=Late] * [Comparison=vHC-vHC] * [Frequency=5.42] 1.909 0.167

[Session=Late] * [Comparison=vHC-vHC] * [Frequency=5.92] 3.220 0.073

[Session=Late] * [Comparison=vHC-vHC] * [Frequency=6.41] 4.376 0.036 *

[Session=Late] * [Comparison=vHC-vHC] * [Frequency=6.90] 5.062 0.024 *

[Session=Late] * [Comparison=vHC-vHC] * [Frequency=7.40] 5.195 0.023 *

[Session=Late] * [Comparison=vHC-vHC] * [Frequency=7.89] 4.738 0.030 *

[Session=Late] * [Comparison=vHC-vHC] * [Frequency=8.38] 3.633 0.057

[Session=Late] * [Comparison=vHC-vHC] * [Frequency=8.88] 2.050 0.152

[Session=Late] * [Comparison=vHC-vHC] * [Frequency=9.37] 0.720 0.396

[Session=Late] * [Comparison=vHC-vHC] * [Frequency=9.86] 0.121 0.727

[Session=Late] * [Comparison=vHC-vHC] * [Frequency=10.36] 0.002 0.965

[Session=Late] * [Comparison=vHC-vHC] * [Frequency=10.85] 0.131 0.717

[Session=Late] * [Comparison=vHC-vHC] * [Frequency=11.34] 0.393 0.531

[Session=Late] * [Comparison=vHC-vHC] * [Frequency=11.84] 0.712 0.399

[Session=Early] * [Comparison=mPFC-mPFC] * [Frequency=7.40] 0a

Extended Table 4-1. Results of GEE Comparison × Epoch interaction for time binned Late training session with ventral hippocampus-medial prefrontal cortex (vHC-mPFC) and dorsal hippocampus-medial prefrontal cortex (dHC-mPFC). Dorsal hippocampus-medial prefrontal cortex at epoch 3 as reference (0a).

Comparison × Epoch Wald value Sig.

[Comparison=vHC-mPFC] * [Epoch=-3] 35.519 0.000 *

[Comparison=vHC-mPFC] * [Epoch=-2] 8.336 0.004 *

[Comparison=vHC-mPFC] * [Epoch=-1] 31.784 0.000 *

[Comparison=vHC-mPFC] * [Epoch=0] 7.860 0.005 *

[Comparison=vHC-mPFC] * [Epoch=1] 2.560 0.110

[Comparison=vHC-mPFC] * [Epoch=2] 6.518 0.011 *

[Comparison=vHC-mPFC] * [Epoch=3] 5.225 0.022 *

[Comparison=dHC-mPFC] * [Epoch=-3] 15.540 0.000 *

[Comparison=dHC-mPFC] * [Epoch=-2] 0.056 0.814

[Comparison=dHC-mPFC] * [Epoch=-1] 12.212 0.000 *

[Comparison=dHC-mPFC] * [Epoch=0] 0.312 0.576

[Comparison=dHC-mPFC] * [Epoch=1] 0.105 0.746

[Comparison=dHC-mPFC] * [Epoch=2] 2.345 0.126

[Comparison=dHC-mPFC] * [Epoch=3] 0a

Extended Table 4-2. Results of GEE Comparison × Epoch × Frequency interaction for time binned Late training session with ventral hippocampus-medial prefrontal cortex (vHC-mPFC) and dorsal hippocampus-medial prefrontal cortex (dHC-mPFC). Dorsal hippocampus-medial prefrontal cortex at epoch 3 and 11.84Hz as reference (0a).

Comparison × Epoch × Frequency Wald value Sig.

[Comparison=vHC-mPFC] * [Epoch=-3] * [Frequency=5.42] 2.284 0.131

[Comparison=vHC-mPFC] * [Epoch=-3] * [Frequency=5.92] 2.365 0.124

[Comparison=vHC-mPFC] * [Epoch=-3] * [Frequency=6.41] 2.493 0.114

[Comparison=vHC-mPFC] * [Epoch=-3] * [Frequency=6.90] 2.659 0.103

[Comparison=vHC-mPFC] * [Epoch=-3] * [Frequency=7.40] 0.716 0.397

[Comparison=vHC-mPFC] * [Epoch=-3] * [Frequency=7.89] 0.020 0.887

[Comparison=vHC-mPFC] * [Epoch=-3] * [Frequency=8.38] 0.000 0.997

[Comparison=vHC-mPFC] * [Epoch=-3] * [Frequency=8.88] 0.000 1.000

[Comparison=vHC-mPFC] * [Epoch=-3] * [Frequency=9.37] 0.234 0.629

[Comparison=vHC-mPFC] * [Epoch=-3] * [Frequency=9.86] 1.430 0.232

[Comparison=vHC-mPFC] * [Epoch=-3] * [Frequency=10.36] 0.960 0.327

[Comparison=vHC-mPFC] * [Epoch=-3] * [Frequency=10.85] 1.160 0.281

[Comparison=vHC-mPFC] * [Epoch=-3] * [Frequency=11.34] 1.543 0.214

[Comparison=vHC-mPFC] * [Epoch=-3] * [Frequency=11.84] 0.618 0.432

[Comparison=vHC-mPFC] * [Epoch=-2] * [Frequency=5.42] 0.017 0.896

[Comparison=vHC-mPFC] * [Epoch=-2] * [Frequency=5.92] 0.002 0.964

[Comparison=vHC-mPFC] * [Epoch=-2] * [Frequency=6.41] 0.506 0.477

[Comparison=vHC-mPFC] * [Epoch=-2] * [Frequency=6.90] 1.070 0.301

[Comparison=vHC-mPFC] * [Epoch=-2] * [Frequency=7.40] 1.603 0.206

[Comparison=vHC-mPFC] * [Epoch=-2] * [Frequency=7.89] 0.745 0.388

[Comparison=vHC-mPFC] * [Epoch=-2] * [Frequency=8.38] 0.125 0.724

[Comparison=vHC-mPFC] * [Epoch=-2] * [Frequency=8.88] 0.164 0.685

[Comparison=vHC-mPFC] * [Epoch=-2] * [Frequency=9.37] 0.378 0.539

[Comparison=vHC-mPFC] * [Epoch=-2] * [Frequency=9.86] 0.003 0.954

[Comparison=vHC-mPFC] * [Epoch=-2] * [Frequency=10.36] 0.008 0.927

[Comparison=vHC-mPFC] * [Epoch=-2] * [Frequency=10.85] 0.045 0.832

[Comparison=vHC-mPFC] * [Epoch=-2] * [Frequency=11.34] 0.562 0.454

[Comparison=vHC-mPFC] * [Epoch=-2] * [Frequency=11.83] 1.024 0.312

[Comparison=vHC-mPFC] * [Epoch=-1] * [Frequency=5.42] 0.424 0.515

[Comparison=vHC-mPFC] * [Epoch=-1] * [Frequency=5.92] 0.283 0.595

[Comparison=vHC-mPFC] * [Epoch=-1] * [Frequency=6.41] 0.007 0.935

[Comparison=vHC-mPFC] * [Epoch=-1] * [Frequency=6.90] 0.021 0.884

[Comparison=vHC-mPFC] * [Epoch=-1] * [Frequency=7.40] 0.468 0.494

[Comparison=vHC-mPFC] * [Epoch=-1] * [Frequency=7.89] 0.828 0.363

[Comparison=vHC-mPFC] * [Epoch=-1] * [Frequency=8.38] 2.392 0.122

[Comparison=vHC-mPFC] * [Epoch=-1] * [Frequency=8.88] 6.295 0.012 *

[Comparison=vHC-mPFC] * [Epoch=-1] * [Frequency=9.37] 7.579 0.006 *

[Comparison=vHC-mPFC] * [Epoch=-1] * [Frequency=9.86] 4.072 0.044 *

[Comparison=vHC-mPFC] * [Epoch=-1] * [Frequency=10.36] 0.347 0.556

[Comparison=vHC-mPFC] * [Epoch=-1] * [Frequency=10.85] 0.254 0.614

[Comparison=vHC-mPFC] * [Epoch=-1] * [Frequency=11.34] 0.063 0.802

[Comparison=vHC-mPFC] * [Epoch=-1] * [Frequency=11.84] 1.026 0.311

[Comparison=vHC-mPFC] * [Epoch=0] * [Frequency=5.42] 0.058 0.809

[Comparison=vHC-mPFC] * [Epoch=0] * [Frequency=5.92] 0.001 0.980

[Comparison=vHC-mPFC] * [Epoch=0] * [Frequency=6.41] 0.001 0.973

[Comparison=vHC-mPFC] * [Epoch=0] * [Frequency=6.90] 0.142 0.706

[Comparison=vHC-mPFC] * [Epoch=0] * [Frequency=7.40] 0.005 0.943

[Comparison=vHC-mPFC] * [Epoch=0] * [Frequency=7.89] 1.404 0.236

[Comparison=vHC-mPFC] * [Epoch=0] * [Frequency=8.38] 0.824 0.364

[Comparison=vHC-mPFC] * [Epoch=0] * [Frequency=8.88] 0.031 0.861

[Comparison=vHC-mPFC] * [Epoch=0] * [Frequency=9.37] 0.186 0.666

[Comparison=vHC-mPFC] * [Epoch=0] * [Frequency=9.86] 0.469 0.493

[Comparison=vHC-mPFC] * [Epoch=0] * [Frequency=10.36] 0.299 0.585

[Comparison=vHC-mPFC] * [Epoch=0] * [Frequency=10.85] 0.046 0.829

[Comparison=vHC-mPFC] * [Epoch=0] * [Frequency=11.34] 0.146 0.703

[Comparison=vHC-mPFC] * [Epoch=0] * [Frequency=11.84] 0.853 0.356

[Comparison=vHC-mPFC] * [Epoch=1] * [Frequency=5.42] 0.001 0.972

[Comparison=vHC-mPFC] * [Epoch=1] * [Frequency=5.92] 0.535 0.464

[Comparison=vHC-mPFC] * [Epoch=1] * [Frequency=6.41] 0.459 0.498

[Comparison=vHC-mPFC] * [Epoch=1] * [Frequency=6.90] 0.016 0.900

[Comparison=vHC-mPFC] * [Epoch=1] * [Frequency=7.40] 0.045 0.832

[Comparison=vHC-mPFC] * [Epoch=1] * [Frequency=7.89] 0.055 0.814

[Comparison=vHC-mPFC] * [Epoch=1] * [Frequency=8.38] 0.296 0.586

[Comparison=vHC-mPFC] * [Epoch=1] * [Frequency=8.88] 0.281 0.596

[Comparison=vHC-mPFC] * [Epoch=1] * [Frequency=9.37] 0.003 0.957

[Comparison=vHC-mPFC] * [Epoch=1] * [Frequency=9.86] 0.264 0.608

[Comparison=vHC-mPFC] * [Epoch=1] * [Frequency=10.36] 0.094 0.759

[Comparison=vHC-mPFC] * [Epoch=1] * [Frequency=10.85] 1.505 0.220

[Comparison=vHC-mPFC] * [Epoch=1] * [Frequency=11.34] 1.315 0.252

[Comparison=vHC-mPFC] * [Epoch=1] * [Frequency=11.84] 0.030 0.863

[Comparison=vHC-mPFC] * [Epoch=2] * [Frequency=5.42] 0.082 0.774

[Comparison=vHC-mPFC] * [Epoch=2] * [Frequency=5.92] 0.499 0.480

[Comparison=vHC-mPFC] * [Epoch=2] * [Frequency=6.41] 1.633 0.201

[Comparison=vHC-mPFC] * [Epoch=2] * [Frequency=6.90] 1.062 0.303

[Comparison=vHC-mPFC] * [Epoch=2] * [Frequency=7.40] 0.011 0.917

[Comparison=vHC-mPFC] * [Epoch=2] * [Frequency=7.89] 0.689 0.406

[Comparison=vHC-mPFC] * [Epoch=2] * [Frequency=8.38] 1.406 0.236

[Comparison=vHC-mPFC] * [Epoch=2] * [Frequency=8.88] 0.232 0.630

[Comparison=vHC-mPFC] * [Epoch=2] * [Frequency=9.37] 0.004 0.950

[Comparison=vHC-mPFC] * [Epoch=2] * [Frequency=9.86] 0.002 0.969

[Comparison=vHC-mPFC] * [Epoch=2] * [Frequency=10.36] 0.087 0.768

[Comparison=vHC-mPFC] * [Epoch=2] * [Frequency=10.85] 0.016 0.900

[Comparison=vHC-mPFC] * [Epoch=2] * [Frequency=11.34] 0.443 0.506

[Comparison=vHC-mPFC] * [Epoch=2] * [Frequency=11.84] 1.938 0.164

[Comparison=vHC-mPFC] * [Epoch=3] * [Frequency=5.42] 0.374 0.541

[Comparison=vHC-mPFC] * [Epoch=3] * [Frequency=5.92] 0.174 0.676

[Comparison=vHC-mPFC] * [Epoch=3] * [Frequency=6.41] 0.017 0.897

[Comparison=vHC-mPFC] * [Epoch=3] * [Frequency=6.90] 0.008 0.931

[Comparison=vHC-mPFC] * [Epoch=3] * [Frequency=7.40] 0.199 0.655

[Comparison=vHC-mPFC] * [Epoch=3] * [Frequency=7.89] 0.063 0.802

[Comparison=vHC-mPFC] * [Epoch=3] * [Frequency=8.38] 0.001 0.973

[Comparison=vHC-mPFC] * [Epoch=3] * [Frequency=8.88] 0.000 0.986

[Comparison=vHC-mPFC] * [Epoch=3] * [Frequency=9.37] 0.055 0.814

[Comparison=vHC-mPFC] * [Epoch=3] * [Frequency=9.86] 0.227 0.634

[Comparison=vHC-mPFC] * [Epoch=3] * [Frequency=10.36] 0.334 0.564

[Comparison=vHC-mPFC] * [Epoch=3] * [Frequency=10.85] 0.235 0.628

[Comparison=vHC-mPFC] * [Epoch=3] * [Frequency=11.34] 0.006 0.940

[Comparison=vHC-mPFC] * [Epoch=3] * [Frequency=11.84] 0.208 0.649

[Comparison=dHC-mPFC] * [Epoch=-3] * [Frequency=5.42] 0.141 0.707

[Comparison=dHC-mPFC] * [Epoch=-3] * [Frequency=5.92] 0.237 0.627

[Comparison=dHC-mPFC] * [Epoch=-3] * [Frequency=6.41] 0.059 0.808

[Comparison=dHC-mPFC] * [Epoch=-3] * [Frequency=6.90] 0.078 0.781

[Comparison=dHC-mPFC] * [Epoch=-3] * [Frequency=7.40] 0.114 0.735

[Comparison=dHC-mPFC] * [Epoch=-3] * [Frequency=7.89] 0.001 0.975

[Comparison=dHC-mPFC] * [Epoch=-3] * [Frequency=8.38] 0.118 0.732

[Comparison=dHC-mPFC] * [Epoch=-3] * [Frequency=8.88] 0.439 0.508

[Comparison=dHC-mPFC] * [Epoch=-3] * [Frequency=9.37] 0.323 0.570

[Comparison=dHC-mPFC] * [Epoch=-3] * [Frequency=9.86] 0.057 0.811

[Comparison=dHC-mPFC] * [Epoch=-3] * [Frequency=10.36] 1.150 0.283

[Comparison=dHC-mPFC] * [Epoch=-3] * [Frequency=10.85] 4.614 0.032 *

[Comparison=dHC-mPFC] * [Epoch=-3] * [Frequency=11.34] 2.293 0.130

[Comparison=dHC-mPFC] * [Epoch=-3] * [Frequency=11.84] 0.533 0.465

[Comparison=dHC-mPFC] * [Epoch=-2] * [Frequency=5.42] 0.378 0.539

[Comparison=dHC-mPFC] * [Epoch=-2] * [Frequency=5.92] 2.004 0.157

[Comparison=dHC-mPFC] * [Epoch=-2] * [Frequency=6.41] 1.826 0.177

[Comparison=dHC-mPFC] * [Epoch=-2] * [Frequency=6.90] 0.406 0.524

[Comparison=dHC-mPFC] * [Epoch=-2] * [Frequency=7.40] 0.030 0.862

[Comparison=dHC-mPFC] * [Epoch=-2] * [Frequency=7.89] 0.523 0.470

[Comparison=dHC-mPFC] * [Epoch=-2] * [Frequency=8.38] 0.003 0.956

[Comparison=dHC-mPFC] * [Epoch=-2] * [Frequency=8.88] 1.163 0.281

[Comparison=dHC-mPFC] * [Epoch=-2] * [Frequency=9.37] 1.136 0.287

[Comparison=dHC-mPFC] * [Epoch=-2] * [Frequency=9.86] 0.171 0.679

[Comparison=dHC-mPFC] * [Epoch=-2] * [Frequency=10.36] 0.341 0.560

[Comparison=dHC-mPFC] * [Epoch=-2] * [Frequency=10.85] 0.036 0.850

[Comparison=dHC-mPFC] * [Epoch=-2] * [Frequency=11.34] 1.627 0.202

[Comparison=dHC-mPFC] * [Epoch=-2] * [Frequency=11.84] 2.329 0.127

[Comparison=dHC-mPFC] * [Epoch=-1] * [Frequency=5.42] 0.066 0.797

[Comparison=dHC-mPFC] * [Epoch=-1] * [Frequency=5.92] 0.257 0.612

[Comparison=dHC-mPFC] * [Epoch=-1] * [Frequency=6.41] 0.125 0.724

[Comparison=dHC-mPFC] * [Epoch=-1] * [Frequency=6.90] 0.003 0.956

[Comparison=dHC-mPFC] * [Epoch=-1] * [Frequency=7.40] 0.050 0.824

[Comparison=dHC-mPFC] * [Epoch=-1] * [Frequency=7.89] 0.075 0.785

[Comparison=dHC-mPFC] * [Epoch=-1] * [Frequency=8.38] 0.002 0.964

[Comparison=dHC-mPFC] * [Epoch=-1] * [Frequency=8.88] 0.692 0.406

[Comparison=dHC-mPFC] * [Epoch=-1] * [Frequency=9.37] 2.336 0.126

[Comparison=dHC-mPFC] * [Epoch=-1] * [Frequency=9.86] 1.439 0.230

[Comparison=dHC-mPFC] * [Epoch=-1] * [Frequency=10.36] 0.153 0.696

[Comparison=dHC-mPFC] * [Epoch=-1] * [Frequency=10.85] 0.240 0.624

[Comparison=dHC-mPFC] * [Epoch=-1] * [Frequency=11.34] 0.221 0.638

[Comparison=dHC-mPFC] * [Epoch=-1] * [Frequency=11.84] 0.037 0.848

[Comparison=dHC-mPFC] * [Epoch=0] * [Frequency=5.42] 1.552 0.213

[Comparison=dHC-mPFC] * [Epoch=0] * [Frequency=5.92] 0.895 0.344

[Comparison=dHC-mPFC] * [Epoch=0] * [Frequency=6.41] 0.526 0.468

[Comparison=dHC-mPFC] * [Epoch=0] * [Frequency=6.90] 0.666 0.414

[Comparison=dHC-mPFC] * [Epoch=0] * [Frequency=7.40] 0.312 0.576

[Comparison=dHC-mPFC] * [Epoch=0] * [Frequency=7.89] 0.188 0.665

[Comparison=dHC-mPFC] * [Epoch=0] * [Frequency=8.38] 0.218 0.641

[Comparison=dHC-mPFC] * [Epoch=0] * [Frequency=8.88] 0.003 0.955

[Comparison=dHC-mPFC] * [Epoch=0] * [Frequency=9.37] 0.025 0.874

[Comparison=dHC-mPFC] * [Epoch=0] * [Frequency=9.86] 0.247 0.619

[Comparison=dHC-mPFC] * [Epoch=0] * [Frequency=10.36] 0.523 0.469

[Comparison=dHC-mPFC] * [Epoch=0] * [Frequency=10.85] 0.786 0.375

[Comparison=dHC-mPFC] * [Epoch=0] * [Frequency=11.34] 0.524 0.469

[Comparison=dHC-mPFC] * [Epoch=0] * [Frequency=11.84] 0.704 0.401

[Comparison=dHC-mPFC] * [Epoch=1] * [Frequency=5.42] 0.474 0.491

[Comparison=dHC-mPFC] * [Epoch=1] * [Frequency=5.92] 0.254 0.614

[Comparison=dHC-mPFC] * [Epoch=1] * [Frequency=6.41] 0.235 0.628

[Comparison=dHC-mPFC] * [Epoch=1] * [Frequency=6.90] 0.017 0.895

[Comparison=dHC-mPFC] * [Epoch=1] * [Frequency=7.40] 0.467 0.494

[Comparison=dHC-mPFC] * [Epoch=1] * [Frequency=7.89] 1.283 0.257

[Comparison=dHC-mPFC] * [Epoch=1] * [Frequency=8.38] 0.802 0.371

[Comparison=dHC-mPFC] * [Epoch=1] * [Frequency=8.88] 0.258 0.612

[Comparison=dHC-mPFC] * [Epoch=1] * [Frequency=9.37] 0.075 0.784

[Comparison=dHC-mPFC] * [Epoch=1] * [Frequency=9.86] 0.243 0.622

[Comparison=dHC-mPFC] * [Epoch=1] * [Frequency=10.36] 0.730 0.393

[Comparison=dHC-mPFC] * [Epoch=1] * [Frequency=10.85] 3.035 0.081

[Comparison=dHC-mPFC] * [Epoch=1] * [Frequency=11.34] 3.204 0.073

[Comparison=dHC-mPFC] * [Epoch=1] * [Frequency=11.84] 1.103 0.294

[Comparison=dHC-mPFC] * [Epoch=2] * [Frequency=5.42] 0.338 0.561

[Comparison=dHC-mPFC] * [Epoch=2] * [Frequency=5.92] 1.298 0.255

[Comparison=dHC-mPFC] * [Epoch=2] * [Frequency=6.41] 2.621 0.105

[Comparison=dHC-mPFC] * [Epoch=2] * [Frequency=6.90] 1.993 0.158

[Comparison=dHC-mPFC] * [Epoch=2] * [Frequency=7.40] 0.571 0.450

[Comparison=dHC-mPFC] * [Epoch=2] * [Frequency=7.89] 0.002 0.963

[Comparison=dHC-mPFC] * [Epoch=2] * [Frequency=8.38] 0.089 0.765

[Comparison=dHC-mPFC] * [Epoch=2] * [Frequency=8.88] 0.160 0.689

[Comparison=dHC-mPFC] * [Epoch=2] * [Frequency=9.37] 0.226 0.635

[Comparison=dHC-mPFC] * [Epoch=2] * [Frequency=9.86] 0.062 0.803

[Comparison=dHC-mPFC] * [Epoch=2] * [Frequency=10.36] 0.046 0.830

[Comparison=dHC-mPFC] * [Epoch=2] * [Frequency=10.85] 0.237 0.626

[Comparison=dHC-mPFC] * [Epoch=2] * [Frequency=11.34] 1.566 0.211

[Comparison=dHC-mPFC] * [Epoch=2] * [Frequency=11.84] 0.785 0.375

[Comparison=dHC-mPFC] * [Epoch=3] * [Frequency=5.42] 0.009 0.925

[Comparison=dHC-mPFC] * [Epoch=3] * [Frequency=5.92] 0.673 0.412

[Comparison=dHC-mPFC] * [Epoch=3] * [Frequency=6.41] 0.615 0.433

[Comparison=dHC-mPFC] * [Epoch=3] * [Frequency=6.90] 0.188 0.664

[Comparison=dHC-mPFC] * [Epoch=3] * [Frequency=7.40] 0.149 0.700

[Comparison=dHC-mPFC] * [Epoch=3] * [Frequency=7.89] 0.082 0.775

[Comparison=dHC-mPFC] * [Epoch=3] * [Frequency=8.38] 0.364 0.547

[Comparison=dHC-mPFC] * [Epoch=3] * [Frequency=8.88] 1.652 0.199

[Comparison=dHC-mPFC] * [Epoch=3] * [Frequency=9.37] 2.079 0.149

[Comparison=dHC-mPFC] * [Epoch=3] * [Frequency=9.86] 1.959 0.162

[Comparison=dHC-mPFC] * [Epoch=3] * [Frequency=10.36] 1.289 0.256

[Comparison=dHC-mPFC] * [Epoch=3] * [Frequency=10.85] 0.326 0.568

[Comparison=dHC-mPFC] * [Epoch=3] * [Frequency=11.34] 0.019 0.891

[Comparison=dHC-mPFC] * [Epoch=3] * [Frequency=11.84] 0a

Extended Table 5-1. Results of GEE Comparison × Epoch interaction for time binned Late training session with dorsal hippocampus, medial prefrontal cortex, and ventral hippocampus homotopics (dHC-dHC, mPFC-mPFC, vHC-vHC) and ipsilateral/contralateral dorsal hippocampus-ventral hippocampus (dHC-vHC_IPSI_, dHC-vHC_CONTRA_). Dorsal hippocampus-ventral hippocampus contralateral at epoch 3 as reference (0a).

Comparison × Epoch Wald value Sig.

[Comparison=dHC-dHC] * [Epoch=-3] 10.860 0.001 *

[Comparison=dHC-dHC] * [Epoch=-2] 6.985 0.008 *

[Comparison=dHC-dHC] * [Epoch=-1] 27.663 0.000 *

[Comparison=dHC-dHC] * [Epoch=0] 11.650 0.001 *

[Comparison=dHC-dHC] * [Epoch=1] 3.371 0.066

[Comparison=dHC-dHC] * [Epoch=2] 3.532 0.060

[Comparison=dHC-dHC] * [Epoch=3] 3.638 0.056

[Comparison=mPFC-mPFC] * [Epoch=-3] 80.885 0.000 *

[Comparison=mPFC-mPFC] * [Epoch=-2] 4.800 0.028 *

[Comparison=mPFC-mPFC] * [Epoch=-1] 36.900 0.000 *

[Comparison=mPFC-mPFC] * [Epoch=0] 14.657 0.000 *

[Comparison=mPFC-mPFC] * [Epoch=1] 42.328 0.000 *

[Comparison=mPFC-mPFC] * [Epoch=2] 46.676 0.000 *

[Comparison=mPFC-mPFC] * [Epoch=3] 59.693 0.000 *

[Comparison=vHC-vHC] * [Epoch=-3] 0.944 0.331

[Comparison=vHC-vHC] * [Epoch=-2] 2.131 0.144

[Comparison=vHC-vHC] * [Epoch=-1] 0.121 0.727

[Comparison=vHC-vHC] * [Epoch=0] 0.001 0.981

[Comparison=vHC-vHC] * [Epoch=1] 0.865 0.352

[Comparison=vHC-vHC] * [Epoch=2] 2.373 0.123

[Comparison=vHC-vHC] * [Epoch=3] 0.859 0.354

[Comparison=dHC-vHC Ipsi] * [Epoch=-3] 0.399 0.528

[Comparison=dHC-vHC Ipsi] * [Epoch=-2] 0.555 0.456

[Comparison=dHC-vHC Ipsi] * [Epoch=-1] 0.003 0.954

[Comparison=dHC-vHC Ipsi] * [Epoch=0] 0.182 0.670

[Comparison=dHC-vHC Ipsi] * [Epoch=1] 0.374 0.541

[Comparison=dHC-vHC Ipsi] * [Epoch=2] 0.113 0.736

[Comparison=dHC-vHC Ipsi] * [Epoch=3] 0.477 0.490

[Comparison=dHC-vHC Contra] * [Epoch=-3] 5.128 0.024 *

[Comparison=dHC-vHC Contra] * [Epoch=-2] 0.242 0.623

[Comparison=dHC-vHC Contra] * [Epoch=-1] 0.008 0.929

[Comparison=dHC-vHC Contra] * [Epoch=0] 1.776 0.183

[Comparison=dHC-vHC Contra] * [Epoch=1] 1.477 0.224

[Comparison=dHC-vHC Contra] * [Epoch=2] 2.585 0.108

[Comparison=dHC-vHC Contra] * [Epoch=3] 0a

Extended Table 5-2. Results of GEE Comparison × Epoch × Frequency interaction for time binned Late training session with dorsal hippocampus, medial prefrontal cortex, and ventral hippocampus homotopics (dHC-dHC, mPFC-mPFC, vHC-vHC). vHC-vHC during Epoch 3 at 11.84 Hz as reference.

Comparison × Epoch × Frequency Wald value Sig.

[Comparison=dHC-dHC] * [Epoch=-3] * [Frequency=5.42] 0.136 0.712

[Comparison=dHC-dHC] * [Epoch=-3] * [Frequency=5.92] 0.179 0.672

[Comparison=dHC-dHC] * [Epoch=-3] * [Frequency=6.41] 0.376 0.540

[Comparison=dHC-dHC] * [Epoch=-3] * [Frequency=6.90] 0.366 0.545

[Comparison=dHC-dHC] * [Epoch=-3] * [Frequency=7.40] 0.323 0.570

[Comparison=dHC-dHC] * [Epoch=-3] * [Frequency=7.89] 0.593 0.441

[Comparison=dHC-dHC] * [Epoch=-3] * [Frequency=8.38] 1.116 0.291

[Comparison=dHC-dHC] * [Epoch=-3] * [Frequency=8.88] 1.612 0.204

[Comparison=dHC-dHC] * [Epoch=-3] * [Frequency=9.37] 1.717 0.190

[Comparison=dHC-dHC] * [Epoch=-3] * [Frequency=9.86] 1.398 0.237

[Comparison=dHC-dHC] * [Epoch=-3] * [Frequency=10.36] 1.004 0.316

[Comparison=dHC-dHC] * [Epoch=-3] * [Frequency=10.85] 0.848 0.357

[Comparison=dHC-dHC] * [Epoch=-3] * [Frequency=11.34] 0.580 0.446

[Comparison=dHC-dHC] * [Epoch=-3] * [Frequency=11.84] 0.523 0.469

[Comparison=dHC-dHC] * [Epoch=-2] * [Frequency=5.42] 0.081 0.776

[Comparison=dHC-dHC] * [Epoch=-2] * [Frequency=5.92] 0.007 0.932

[Comparison=dHC-dHC] * [Epoch=-2] * [Frequency=6.41] 0.006 0.939

[Comparison=dHC-dHC] * [Epoch=-2] * [Frequency=6.90] 0.129 0.720

[Comparison=dHC-dHC] * [Epoch=-2] * [Frequency=7.40] 0.269 0.604

[Comparison=dHC-dHC] * [Epoch=-2] * [Frequency=7.89] 0.352 0.553

[Comparison=dHC-dHC] * [Epoch=-2] * [Frequency=8.38] 0.758 0.384

[Comparison=dHC-dHC] * [Epoch=-2] * [Frequency=8.88] 1.205 0.272

[Comparison=dHC-dHC] * [Epoch=-2] * [Frequency=9.37] 1.202 0.273

[Comparison=dHC-dHC] * [Epoch=-2] * [Frequency=9.86] 1.249 0.264

[Comparison=dHC-dHC] * [Epoch=-2] * [Frequency=10.36] 1.393 0.238

[Comparison=dHC-dHC] * [Epoch=-2] * [Frequency=10.85] 1.376 0.241

[Comparison=dHC-dHC] * [Epoch=-2] * [Frequency=11.34] 1.107 0.293

[Comparison=dHC-dHC] * [Epoch=-2] * [Frequency=11.84] 0.703 0.402

[Comparison=dHC-dHC] * [Epoch=-1] * [Frequency=5.42] 1.035 0.309

[Comparison=dHC-dHC] * [Epoch=-1] * [Frequency=5.92] 0.914 0.339

[Comparison=dHC-dHC] * [Epoch=-1] * [Frequency=6.41] 0.876 0.349

[Comparison=dHC-dHC] * [Epoch=-1] * [Frequency=6.90] 0.895 0.344

[Comparison=dHC-dHC] * [Epoch=-1] * [Frequency=7.40] 1.111 0.292

[Comparison=dHC-dHC] * [Epoch=-1] * [Frequency=7.89] 1.360 0.244

[Comparison=dHC-dHC] * [Epoch=-1] * [Frequency=8.38] 1.549 0.213

[Comparison=dHC-dHC] * [Epoch=-1] * [Frequency=8.88] 1.747 0.186

[Comparison=dHC-dHC] * [Epoch=-1] * [Frequency=9.37] 2.089 0.148

[Comparison=dHC-dHC] * [Epoch=-1] * [Frequency=9.86] 2.263 0.133

[Comparison=dHC-dHC] * [Epoch=-1] * [Frequency=10.36] 2.106 0.147

[Comparison=dHC-dHC] * [Epoch=-1] * [Frequency=10.85] 1.722 0.189

[Comparison=dHC-dHC] * [Epoch=-1] * [Frequency=11.34] 1.326 0.249

[Comparison=dHC-dHC] * [Epoch=-1] * [Frequency=11.84] 1.345 0.246

[Comparison=dHC-dHC] * [Epoch=0] * [Frequency=5.42] 0.923 0.337

[Comparison=dHC-dHC] * [Epoch=0] * [Frequency=5.92] 0.710 0.400

[Comparison=dHC-dHC] * [Epoch=0] * [Frequency=6.41] 0.528 0.467

[Comparison=dHC-dHC] * [Epoch=0] * [Frequency=6.90] 0.541 0.462

[Comparison=dHC-dHC] * [Epoch=0] * [Frequency=7.40] 0.847 0.358

[Comparison=dHC-dHC] * [Epoch=0] * [Frequency=7.89] 1.032 0.310

[Comparison=dHC-dHC] * [Epoch=0] * [Frequency=8.38] 0.936 0.333

[Comparison=dHC-dHC] * [Epoch=0] * [Frequency=8.88] 0.986 0.321

[Comparison=dHC-dHC] * [Epoch=0] * [Frequency=9.37] 0.743 0.389

[Comparison=dHC-dHC] * [Epoch=0] * [Frequency=9.86] 0.467 0.495

[Comparison=dHC-dHC] * [Epoch=0] * [Frequency=10.36] 0.384 0.535

[Comparison=dHC-dHC] * [Epoch=0] * [Frequency=10.85] 0.586 0.444

[Comparison=dHC-dHC] * [Epoch=0] * [Frequency=11.34] 0.865 0.352

[Comparison=dHC-dHC] * [Epoch=0] * [Frequency=11.84] 1.033 0.309

[Comparison=dHC-dHC] * [Epoch=1] * [Frequency=5.42] 0.013 0.909

[Comparison=dHC-dHC] * [Epoch=1] * [Frequency=5.92] 0.008 0.930

[Comparison=dHC-dHC] * [Epoch=1] * [Frequency=6.41] 0.019 0.891

[Comparison=dHC-dHC] * [Epoch=1] * [Frequency=6.90] 0.272 0.602

[Comparison=dHC-dHC] * [Epoch=1] * [Frequency=7.40] 0.574 0.449

[Comparison=dHC-dHC] * [Epoch=1] * [Frequency=7.89] 0.466 0.495

[Comparison=dHC-dHC] * [Epoch=1] * [Frequency=8.38] 0.267 0.605

[Comparison=dHC-dHC] * [Epoch=1] * [Frequency=8.88] 0.399 0.528

[Comparison=dHC-dHC] * [Epoch=1] * [Frequency=9.37] 0.792 0.373

[Comparison=dHC-dHC] * [Epoch=1] * [Frequency=9.86] 1.161 0.281

[Comparison=dHC-dHC] * [Epoch=1] * [Frequency=10.36] 1.059 0.303

[Comparison=dHC-dHC] * [Epoch=1] * [Frequency=10.85] 0.524 0.469

[Comparison=dHC-dHC] * [Epoch=1] * [Frequency=11.34] 0.254 0.615

[Comparison=dHC-dHC] * [Epoch=1] * [Frequency=11.84] 0.108 0.742

[Comparison=dHC-dHC] * [Epoch=2] * [Frequency=5.42] 0.064 0.801

[Comparison=dHC-dHC] * [Epoch=2] * [Frequency=5.92] 0.232 0.630

[Comparison=dHC-dHC] * [Epoch=2] * [Frequency=6.41] 0.169 0.681

[Comparison=dHC-dHC] * [Epoch=2] * [Frequency=6.90] 0.193 0.661

[Comparison=dHC-dHC] * [Epoch=2] * [Frequency=7.40] 0.246 0.620

[Comparison=dHC-dHC] * [Epoch=2] * [Frequency=7.89] 0.223 0.637

[Comparison=dHC-dHC] * [Epoch=2] * [Frequency=8.38] 0.303 0.582

[Comparison=dHC-dHC] * [Epoch=2] * [Frequency=8.88] 0.565 0.452

[Comparison=dHC-dHC] * [Epoch=2] * [Frequency=9.37] 0.771 0.380

[Comparison=dHC-dHC] * [Epoch=2] * [Frequency=9.86] 0.722 0.396

[Comparison=dHC-dHC] * [Epoch=2] * [Frequency=10.36] 0.385 0.535

[Comparison=dHC-dHC] * [Epoch=2] * [Frequency=10.85] 0.184 0.668

[Comparison=dHC-dHC] * [Epoch=2] * [Frequency=11.34] 0.387 0.534

[Comparison=dHC-dHC] * [Epoch=2] * [Frequency=11.84] 0.738 0.390

[Comparison=dHC-dHC] * [Epoch=3] * [Frequency=5.42] 0.174 0.677

[Comparison=dHC-dHC] * [Epoch=3] * [Frequency=5.92] 0.186 0.666

[Comparison=dHC-dHC] * [Epoch=3] * [Frequency=6.41] 0.110 0.740

[Comparison=dHC-dHC] * [Epoch=3] * [Frequency=6.90] 0.159 0.690

[Comparison=dHC-dHC] * [Epoch=3] * [Frequency=7.40] 0.418 0.518

[Comparison=dHC-dHC] * [Epoch=3] * [Frequency=7.89] 0.798 0.372

[Comparison=dHC-dHC] * [Epoch=3] * [Frequency=8.38] 0.675 0.411

[Comparison=dHC-dHC] * [Epoch=3] * [Frequency=8.88] 0.641 0.424

[Comparison=dHC-dHC] * [Epoch=3] * [Frequency=9.37] 1.001 0.317

[Comparison=dHC-dHC] * [Epoch=3] * [Frequency=9.86] 1.136 0.286

[Comparison=dHC-dHC] * [Epoch=3] * [Frequency=10.36] 0.664 0.415

[Comparison=dHC-dHC] * [Epoch=3] * [Frequency=10.85] 0.184 0.668

[Comparison=dHC-dHC] * [Epoch=3] * [Frequency=11.34] 0.000 1.000

[Comparison=dHC-dHC] * [Epoch=3] * [Frequency=11.84] 0.002 0.961

[Comparison=mPFC-mPFC] * [Epoch=-3] * [Frequency=5.42] 5.101 0.024 *

[Comparison=mPFC-mPFC] * [Epoch=-3] * [Frequency=5.92] 5.484 0.019 *

[Comparison=mPFC-mPFC] * [Epoch=-3] * [Frequency=6.41] 5.517 0.019 *

[Comparison=mPFC-mPFC] * [Epoch=-3] * [Frequency=6.90] 8.383 0.004 *

[Comparison=mPFC-mPFC] * [Epoch=-3] * [Frequency=7.40] 8.581 0.003 *

[Comparison=mPFC-mPFC] * [Epoch=-3] * [Frequency=7.89] 5.536 0.019 *

[Comparison=mPFC-mPFC] * [Epoch=-3] * [Frequency=8.38] 2.392 0.122

[Comparison=mPFC-mPFC] * [Epoch=-3] * [Frequency=8.88] 0.204 0.651

[Comparison=mPFC-mPFC] * [Epoch=-3] * [Frequency=9.37] 0.015 0.904

[Comparison=mPFC-mPFC] * [Epoch=-3] * [Frequency=9.86] 0.000 0.999

[Comparison=mPFC-mPFC] * [Epoch=-3] * [Frequency=10.36] 0.069 0.793

[Comparison=mPFC-mPFC] * [Epoch=-3] * [Frequency=10.85] 0.735 0.391

[Comparison=mPFC-mPFC] * [Epoch=-3] * [Frequency=11.34] 3.228 0.072

[Comparison=mPFC-mPFC] * [Epoch=-3] * [Frequency=11.84] 3.964 0.046

[Comparison=mPFC-mPFC] * [Epoch=-2] * [Frequency=5.42] 0.049 0.825

[Comparison=mPFC-mPFC] * [Epoch=-2] * [Frequency=5.92] 0.001 0.979

[Comparison=mPFC-mPFC] * [Epoch=-2] * [Frequency=6.41] 0.165 0.684

[Comparison=mPFC-mPFC] * [Epoch=-2] * [Frequency=6.90] 0.264 0.608

[Comparison=mPFC-mPFC] * [Epoch=-2] * [Frequency=7.40] 0.080 0.778

[Comparison=mPFC-mPFC] * [Epoch=-2] * [Frequency=7.89] 0.005 0.943

[Comparison=mPFC-mPFC] * [Epoch=-2] * [Frequency=8.38] 0.000 0.998

[Comparison=mPFC-mPFC] * [Epoch=-2] * [Frequency=8.88] 0.056 0.813

[Comparison=mPFC-mPFC] * [Epoch=-2] * [Frequency=9.37] 0.102 0.749

[Comparison=mPFC-mPFC] * [Epoch=-2] * [Frequency=9.86] 0.431 0.511

[Comparison=mPFC-mPFC] * [Epoch=-2] * [Frequency=10.36] 0.127 0.721

[Comparison=mPFC-mPFC] * [Epoch=-2] * [Frequency=10.85] 0.013 0.910

[Comparison=mPFC-mPFC] * [Epoch=-2] * [Frequency=11.34] 0.017 0.896

[Comparison=mPFC-mPFC] * [Epoch=-2] * [Frequency=11.84] 0.000 0.996

[Comparison=mPFC-mPFC] * [Epoch=-1] * [Frequency=5.42] 0.471 0.493

[Comparison=mPFC-mPFC] * [Epoch=-1] * [Frequency=5.92] 0.508 0.476

[Comparison=mPFC-mPFC] * [Epoch=-1] * [Frequency=6.41] 0.600 0.439

[Comparison=mPFC-mPFC] * [Epoch=-1] * [Frequency=6.90] 0.593 0.441

[Comparison=mPFC-mPFC] * [Epoch=-1] * [Frequency=7.40] 0.553 0.457

[Comparison=mPFC-mPFC] * [Epoch=-1] * [Frequency=7.89] 0.457 0.499

[Comparison=mPFC-mPFC] * [Epoch=-1] * [Frequency=8.38] 0.359 0.549

[Comparison=mPFC-mPFC] * [Epoch=-1] * [Frequency=8.88] 0.477 0.490

[Comparison=mPFC-mPFC] * [Epoch=-1] * [Frequency=9.37] 1.123 0.289

[Comparison=mPFC-mPFC] * [Epoch=-1] * [Frequency=9.86] 1.913 0.167

[Comparison=mPFC-mPFC] * [Epoch=-1] * [Frequency=10.36] 1.904 0.168

[Comparison=mPFC-mPFC] * [Epoch=-1] * [Frequency=10.85] 0.948 0.330

[Comparison=mPFC-mPFC] * [Epoch=-1] * [Frequency=11.34] 0.755 0.385

[Comparison=mPFC-mPFC] * [Epoch=-1] * [Frequency=11.84] 0.572 0.450

[Comparison=mPFC-mPFC] * [Epoch=0] * [Frequency=5.42] 0.220 0.639

[Comparison=mPFC-mPFC] * [Epoch=0] * [Frequency=5.92] 0.287 0.592

[Comparison=mPFC-mPFC] * [Epoch=0] * [Frequency=6.41] 0.442 0.506

[Comparison=mPFC-mPFC] * [Epoch=0] * [Frequency=6.90] 0.952 0.329

[Comparison=mPFC-mPFC] * [Epoch=0] * [Frequency=7.40] 1.044 0.307

[Comparison=mPFC-mPFC] * [Epoch=0] * [Frequency=7.89] 0.243 0.622

[Comparison=mPFC-mPFC] * [Epoch=0] * [Frequency=8.38] 0.002 0.967

[Comparison=mPFC-mPFC] * [Epoch=0] * [Frequency=8.88] 0.000 0.998

[Comparison=mPFC-mPFC] * [Epoch=0] * [Frequency=9.37] 0.014 0.907

[Comparison=mPFC-mPFC] * [Epoch=0] * [Frequency=9.86] 0.162 0.687

[Comparison=mPFC-mPFC] * [Epoch=0] * [Frequency=10.36] 0.355 0.551

[Comparison=mPFC-mPFC] * [Epoch=0] * [Frequency=10.85] 0.396 0.529

[Comparison=mPFC-mPFC] * [Epoch=0] * [Frequency=11.34] 0.180 0.671

[Comparison=mPFC-mPFC] * [Epoch=0] * [Frequency=11.84] 0.010 0.918

[Comparison=mPFC-mPFC] * [Epoch=1] * [Frequency=5.42] 1.288 0.256

[Comparison=mPFC-mPFC] * [Epoch=1] * [Frequency=5.92] 1.408 0.235

[Comparison=mPFC-mPFC] * [Epoch=1] * [Frequency=6.41] 0.891 0.345

[Comparison=mPFC-mPFC] * [Epoch=1] * [Frequency=6.90] 0.257 0.612

[Comparison=mPFC-mPFC] * [Epoch=1] * [Frequency=7.40] 0.230 0.631

[Comparison=mPFC-mPFC] * [Epoch=1] * [Frequency=7.89] 0.737 0.391

[Comparison=mPFC-mPFC] * [Epoch=1] * [Frequency=8.38] 0.941 0.332

[Comparison=mPFC-mPFC] * [Epoch=1] * [Frequency=8.88] 0.603 0.437

[Comparison=mPFC-mPFC] * [Epoch=1] * [Frequency=9.37] 0.660 0.417

[Comparison=mPFC-mPFC] * [Epoch=1] * [Frequency=9.86] 1.831 0.176

[Comparison=mPFC-mPFC] * [Epoch=1] * [Frequency=10.36] 3.509 0.061

[Comparison=mPFC-mPFC] * [Epoch=1] * [Frequency=10.85] 1.304 0.253

[Comparison=mPFC-mPFC] * [Epoch=1] * [Frequency=11.34] 0.388 0.534

[Comparison=mPFC-mPFC] * [Epoch=1] * [Frequency=11.84] 0.443 0.506

[Comparison=mPFC-mPFC] * [Epoch=2] * [Frequency=5.42] 3.376 0.066

[Comparison=mPFC-mPFC] * [Epoch=2] * [Frequency=5.92] 3.190 0.074

[Comparison=mPFC-mPFC] * [Epoch=2] * [Frequency=6.41] 2.067 0.151

[Comparison=mPFC-mPFC] * [Epoch=2] * [Frequency=6.90] 1.359 0.244

[Comparison=mPFC-mPFC] * [Epoch=2] * [Frequency=7.40] 0.957 0.328

[Comparison=mPFC-mPFC] * [Epoch=2] * [Frequency=7.89] 0.727 0.394

[Comparison=mPFC-mPFC] * [Epoch=2] * [Frequency=8.38] 0.539 0.463

[Comparison=mPFC-mPFC] * [Epoch=2] * [Frequency=8.88] 0.283 0.595

[Comparison=mPFC-mPFC] * [Epoch=2] * [Frequency=9.37] 0.191 0.662

[Comparison=mPFC-mPFC] * [Epoch=2] * [Frequency=9.86] 0.078 0.781

[Comparison=mPFC-mPFC] * [Epoch=2] * [Frequency=10.36] 0.273 0.601

[Comparison=mPFC-mPFC] * [Epoch=2] * [Frequency=10.85] 0.879 0.348

[Comparison=mPFC-mPFC] * [Epoch=2] * [Frequency=11.34] 1.836 0.175

[Comparison=mPFC-mPFC] * [Epoch=2] * [Frequency=11.84] 2.434 0.119

[Comparison=mPFC-mPFC] * [Epoch=3] * [Frequency=5.42] 6.572 0.010 *

[Comparison=mPFC-mPFC] * [Epoch=3] * [Frequency=5.92] 4.114 0.043 *

[Comparison=mPFC-mPFC] * [Epoch=3] * [Frequency=6.41] 1.418 0.234

[Comparison=mPFC-mPFC] * [Epoch=3] * [Frequency=6.90] 0.595 0.441

[Comparison=mPFC-mPFC] * [Epoch=3] * [Frequency=7.40] 0.510 0.475

[Comparison=mPFC-mPFC] * [Epoch=3] * [Frequency=7.89] 0.367 0.545

[Comparison=mPFC-mPFC] * [Epoch=3] * [Frequency=8.38] 0.516 0.473

[Comparison=mPFC-mPFC] * [Epoch=3] * [Frequency=8.88] 0.825 0.364

[Comparison=mPFC-mPFC] * [Epoch=3] * [Frequency=9.37] 1.702 0.192

[Comparison=mPFC-mPFC] * [Epoch=3] * [Frequency=9.86] 2.545 0.111

[Comparison=mPFC-mPFC] * [Epoch=3] * [Frequency=10.36] 1.837 0.175

[Comparison=mPFC-mPFC] * [Epoch=3] * [Frequency=10.85] 0.951 0.330

[Comparison=mPFC-mPFC] * [Epoch=3] * [Frequency=11.34] 0.561 0.454

[Comparison=mPFC-mPFC] * [Epoch=3] * [Frequency=11.84] 1.646 0.199

[Comparison=vHC-vHC] * [Epoch=-3] * [Frequency=5.42] 0.002 0.968

[Comparison=vHC-vHC] * [Epoch=-3] * [Frequency=5.92] 0.179 0.672

[Comparison=vHC-vHC] * [Epoch=-3] * [Frequency=6.41] 0.546 0.460

[Comparison=vHC-vHC] * [Epoch=-3] * [Frequency=6.90] 0.563 0.453

[Comparison=vHC-vHC] * [Epoch=-3] * [Frequency=7.40] 0.335 0.563

[Comparison=vHC-vHC] * [Epoch=-3] * [Frequency=7.89] 0.188 0.665

[Comparison=vHC-vHC] * [Epoch=-3] * [Frequency=8.38] 0.131 0.717

[Comparison=vHC-vHC] * [Epoch=-3] * [Frequency=8.88] 0.160 0.689

[Comparison=vHC-vHC] * [Epoch=-3] * [Frequency=9.37] 0.133 0.715

[Comparison=vHC-vHC] * [Epoch=-3] * [Frequency=9.86] 0.148 0.700

[Comparison=vHC-vHC] * [Epoch=-3] * [Frequency=10.36] 0.255 0.614

[Comparison=vHC-vHC] * [Epoch=-3] * [Frequency=10.85] 0.259 0.611

[Comparison=vHC-vHC] * [Epoch=-3] * [Frequency=11.34] 0.059 0.809

[Comparison=vHC-vHC] * [Epoch=-3] * [Frequency=11.84] 0.021 0.885

[Comparison=vHC-vHC] * [Epoch=-2] * [Frequency=5.42] 0.000 0.995

[Comparison=vHC-vHC] * [Epoch=-2] * [Frequency=5.92] 0.006 0.939

[Comparison=vHC-vHC] * [Epoch=-2] * [Frequency=6.41] 0.013 0.909

[Comparison=vHC-vHC] * [Epoch=-2] * [Frequency=6.90] 0.133 0.716

[Comparison=vHC-vHC] * [Epoch=-2] * [Frequency=7.40] 0.281 0.596

[Comparison=vHC-vHC] * [Epoch=-2] * [Frequency=7.89] 0.704 0.401

[Comparison=vHC-vHC] * [Epoch=-2] * [Frequency=8.38] 0.881 0.348

[Comparison=vHC-vHC] * [Epoch=-2] * [Frequency=8.88] 0.478 0.490

[Comparison=vHC-vHC] * [Epoch=-2] * [Frequency=9.37] 0.327 0.567

[Comparison=vHC-vHC] * [Epoch=-2] * [Frequency=9.86] 0.388 0.533

[Comparison=vHC-vHC] * [Epoch=-2] * [Frequency=10.36] 0.544 0.461

[Comparison=vHC-vHC] * [Epoch=-2] * [Frequency=10.85] 0.635 0.425

[Comparison=vHC-vHC] * [Epoch=-2] * [Frequency=11.34] 0.319 0.572

[Comparison=vHC-vHC] * [Epoch=-2] * [Frequency=11.84] 0.005 0.941

[Comparison=vHC-vHC] * [Epoch=-1] * [Frequency=5.42] 0.305 0.581

[Comparison=vHC-vHC] * [Epoch=-1] * [Frequency=5.92] 0.227 0.634

[Comparison=vHC-vHC] * [Epoch=-1] * [Frequency=6.41] 0.056 0.813

[Comparison=vHC-vHC] * [Epoch=-1] * [Frequency=6.90] 0.002 0.960

[Comparison=vHC-vHC] * [Epoch=-1] * [Frequency=7.40] 0.006 0.937

[Comparison=vHC-vHC] * [Epoch=-1] * [Frequency=7.89] 0.029 0.864

[Comparison=vHC-vHC] * [Epoch=-1] * [Frequency=8.38] 0.015 0.902

[Comparison=vHC-vHC] * [Epoch=-1] * [Frequency=8.88] 0.005 0.946

[Comparison=vHC-vHC] * [Epoch=-1] * [Frequency=9.37] 0.041 0.840

[Comparison=vHC-vHC] * [Epoch=-1] * [Frequency=9.86] 0.084 0.772

[Comparison=vHC-vHC] * [Epoch=-1] * [Frequency=10.36] 0.166 0.684

[Comparison=vHC-vHC] * [Epoch=-1] * [Frequency=10.85] 0.238 0.626

[Comparison=vHC-vHC] * [Epoch=-1] * [Frequency=11.34] 0.315 0.575

[Comparison=vHC-vHC] * [Epoch=-1] * [Frequency=11.84] 0.283 0.595

[Comparison=vHC-vHC] * [Epoch=0] * [Frequency=5.42] 0.015 0.903

[Comparison=vHC-vHC] * [Epoch=0] * [Frequency=5.92] 0.123 0.726

[Comparison=vHC-vHC] * [Epoch=0] * [Frequency=6.41] 0.345 0.557

[Comparison=vHC-vHC] * [Epoch=0] * [Frequency=6.90] 0.368 0.544

[Comparison=vHC-vHC] * [Epoch=0] * [Frequency=7.40] 0.233 0.630

[Comparison=vHC-vHC] * [Epoch=0] * [Frequency=7.89] 0.077 0.782

[Comparison=vHC-vHC] * [Epoch=0] * [Frequency=8.38] 0.007 0.932

[Comparison=vHC-vHC] * [Epoch=0] * [Frequency=8.88] 0.011 0.916

[Comparison=vHC-vHC] * [Epoch=0] * [Frequency=9.37] 0.061 0.804

[Comparison=vHC-vHC] * [Epoch=0] * [Frequency=9.86] 0.117 0.732

[Comparison=vHC-vHC] * [Epoch=0] * [Frequency=10.36] 0.102 0.750

[Comparison=vHC-vHC] * [Epoch=0] * [Frequency=10.85] 0.008 0.931

[Comparison=vHC-vHC] * [Epoch=0] * [Frequency=11.34] 0.035 0.851

[Comparison=vHC-vHC] * [Epoch=0] * [Frequency=11.84] 0.032 0.857

[Comparison=vHC-vHC] * [Epoch=1] * [Frequency=5.42] 0.661 0.416

[Comparison=vHC-vHC] * [Epoch=1] * [Frequency=5.92] 0.644 0.422

[Comparison=vHC-vHC] * [Epoch=1] * [Frequency=6.41] 0.061 0.804

[Comparison=vHC-vHC] * [Epoch=1] * [Frequency=6.90] 0.039 0.843

[Comparison=vHC-vHC] * [Epoch=1] * [Frequency=7.40] 0.085 0.771

[Comparison=vHC-vHC] * [Epoch=1] * [Frequency=7.89] 0.141 0.707

[Comparison=vHC-vHC] * [Epoch=1] * [Frequency=8.38] 0.308 0.579

[Comparison=vHC-vHC] * [Epoch=1] * [Frequency=8.88] 0.503 0.478

[Comparison=vHC-vHC] * [Epoch=1] * [Frequency=9.37] 0.622 0.430

[Comparison=vHC-vHC] * [Epoch=1] * [Frequency=9.86] 0.699 0.403

[Comparison=vHC-vHC] * [Epoch=1] * [Frequency=10.36] 0.805 0.370

[Comparison=vHC-vHC] * [Epoch=1] * [Frequency=10.85] 0.831 0.362

[Comparison=vHC-vHC] * [Epoch=1] * [Frequency=11.34] 0.701 0.403

[Comparison=vHC-vHC] * [Epoch=1] * [Frequency=11.84] 0.293 0.589

[Comparison=vHC-vHC] * [Epoch=2] * [Frequency=5.42] 0.004 0.951

[Comparison=vHC-vHC] * [Epoch=2] * [Frequency=5.92] 0.072 0.788

[Comparison=vHC-vHC] * [Epoch=2] * [Frequency=6.41] 0.133 0.715

[Comparison=vHC-vHC] * [Epoch=2] * [Frequency=6.90] 0.254 0.614

[Comparison=vHC-vHC] * [Epoch=2] * [Frequency=7.40] 0.472 0.492

[Comparison=vHC-vHC] * [Epoch=2] * [Frequency=7.89] 0.373 0.541

[Comparison=vHC-vHC] * [Epoch=2] * [Frequency=8.38] 0.166 0.683

[Comparison=vHC-vHC] * [Epoch=2] * [Frequency=8.88] 0.277 0.599

[Comparison=vHC-vHC] * [Epoch=2] * [Frequency=9.37] 0.530 0.466

[Comparison=vHC-vHC] * [Epoch=2] * [Frequency=9.86] 0.532 0.466

[Comparison=vHC-vHC] * [Epoch=2] * [Frequency=10.36] 0.478 0.489

[Comparison=vHC-vHC] * [Epoch=2] * [Frequency=10.85] 0.508 0.476

[Comparison=vHC-vHC] * [Epoch=2] * [Frequency=11.34] 0.422 0.516

[Comparison=vHC-vHC] * [Epoch=2] * [Frequency=11.84] 0.090 0.764

[Comparison=vHC-vHC] * [Epoch=3] * [Frequency=5.42] 0.011 0.917

[Comparison=vHC-vHC] * [Epoch=3] * [Frequency=5.92] 0.065 0.799

[Comparison=vHC-vHC] * [Epoch=3] * [Frequency=6.41] 0.227 0.634

[Comparison=vHC-vHC] * [Epoch=3] * [Frequency=6.90] 0.341 0.559

[Comparison=vHC-vHC] * [Epoch=3] * [Frequency=7.40] 0.265 0.607

[Comparison=vHC-vHC] * [Epoch=3] * [Frequency=7.89] 0.146 0.703

[Comparison=vHC-vHC] * [Epoch=3] * [Frequency=8.38] 0.049 0.825

[Comparison=vHC-vHC] * [Epoch=3] * [Frequency=8.88] 0.181 0.671

[Comparison=vHC-vHC] * [Epoch=3] * [Frequency=9.37] 0.577 0.448

[Comparison=vHC-vHC] * [Epoch=3] * [Frequency=9.86] 0.750 0.386

[Comparison=vHC-vHC] * [Epoch=3] * [Frequency=10.36] 0.493 0.482

[Comparison=vHC-vHC] * [Epoch=3] * [Frequency=10.85] 0.129 0.719

[Comparison=vHC-vHC] * [Epoch=3] * [Frequency=11.34] 0.001 0.980

[Comparison=vHC-vHC] * [Epoch=3] * [Frequency=11.84] 0a
